# Geographic Differences in Time to Culture Conversion in Liquid Media: Tuberculosis Trials Consortium Study 28. Culture Conversion Is Delayed in Africa

**DOI:** 10.1371/journal.pone.0018358

**Published:** 2011-04-11

**Authors:** William R. Mac Kenzie, Charles M. Heilig, Lorna Bozeman, John L. Johnson, Grace Muzanye, Denise Dunbar, Kenneth C. Jost, Lois Diem, Beverly Metchock, Kathleen Eisenach, Susan Dorman, Stefan Goldberg

**Affiliations:** 1 Tuberculosis Trials Consortium, Division of Tuberculosis Elimination, Centers for Disease Control, Atlanta, Georgia, United States of America; 2 Tuberculosis Research Unit, Case Western Reserve University School of Medicine, Cleveland, Ohio, United States of America; 3 Uganda-Case Western Reserve University Research Collaboration, Makerere University, Mulago Hospital, Kampala, Uganda, United States of America; 4 Mycobacteriology Mycology Laboratory, Texas Department of State Health Services, Austin, Texas, United States of America; 5 Laboratory Branch, Division of Tuberculosis Elimination, Centers for Disease Control, Atlanta, Georgia, United States of America; 6 University of Arkansas for Medical Sciences, Little Rock, Arkansas, United States of America; 7 Johns Hopkins University School of Medicine, Baltimore, Maryland, United States of America; McGill University, Canada

## Abstract

**Background:**

Tuberculosis Trials Consortium Study 28, was a double blind, randomized, placebo-controlled, phase 2 clinical trial examining smear positive pulmonary *Mycobacterium tuberculosis.* Over the course of intensive phase therapy, patients from African sites had substantially delayed and lower rates of culture conversion to negative in liquid media compared to non-African patients. We explored potential explanations of this finding.

**Methods:**

In TBTC Study 28, protocol-correct patients (n = 328) provided spot sputum specimens for *M. tuberculosis* culture in liquid media, at baseline and weeks 2, 4, 6 and 8 of study therapy. We compared sputum culture conversion for African and non-African patients stratified by four baseline measures of disease severity: AFB smear quantification, extent of disease on chest radiograph, cavity size and the number of days to detection of *M. tuberculosis* in liquid media using the Kaplan-Meier product-limit method. We evaluated specimen processing and culture procedures used at 29 study laboratories serving 27 sites.

**Results:**

African TB patients had more extensive disease at enrollment than non-African patients. However, African patients with the least disease by the 4 measures of disease severity had conversion rates on liquid media that were substantially lower than conversion rates in non-African patients with the greatest extent of disease. HIV infection, smoking and diabetes did not explain delayed conversion in Africa. Some inter-site variation in laboratory processing and culture procedures within accepted practice for clinical diagnostic laboratories was found.

**Conclusions:**

Compared with patients from non-African sites, African patients being treated for TB had delayed sputum culture conversion and lower sputum conversion rates in liquid media that were not explained by baseline severity of disease, HIV status, age, smoking, diabetes or race. Further investigation is warranted into whether modest variation in laboratory processes substantially influences the efficacy outcomes of phase 2 TB treatment trials or if other factors (e.g., nutrition, host response) are involved.

**Trial Registration:**

ClinicalTrials.gov NCT00144417

## Introduction

Two month culture conversion has been used as a measure of efficacy in the assessment of drugs used to treat TB in phase 2 trials [1]. The timing of culture conversion is influenced by bacillary quantitation on smear and extent of disease on chest radiograph (CXR), particularly cavitation [2], [3]. More recently, lower rates of culture conversion have been associated with diabetes [3], [4], [5], lack of directly observed therapy [6], HIV infection [6] and smoking [7], [8].

The TB Trials Consortium (TBTC) recently completed a double-blind, randomized, placebo-controlled, phase 2 trial examining the substitution of moxifloxacin for isoniazid (INH) during the first 2 months (intensive phase) of therapy of drug-susceptible, AFB smear-positive pulmonary tuberculosis (TB) [9]. The study was conducted at urban sites in Brazil, Canada, South Africa, Spain, Uganda and the United States. In this study, the proportion of patients with negative cultures at 8 weeks of therapy did not differ significantly between patients in the INH and moxifloxacin arms [9]. In multivariate analysis, positive sputum culture at 8-weeks was associated with enrollment in Africa, cavitation on baseline CXR, higher bacillary load on baseline smear and increasing age, but not HIV co-infection.

A difference in the rate of culture conversion between patients at African and non-African sites was also observed in TBTC Study 27, a phase 2 clinical trial in which moxifloxacin was substituted for ethambutol during the first 2 months of therapy of drug-susceptible smear positive TB [10].

In both TBTC Studies 27 and 28, the definition of sputum culture conversion was based upon the combined results of both liquid and solid media such that a positive result on either media type was considered positive. Since liquid media are more sensitive than solid media in detecting the growth of *M. tuberculosis*, culture status in Studies 27 and 28 was most influenced by the liquid media results.

We explored potential contributions of the following factors to explain lower culture conversion rates in Africa: 1) baseline severity of disease, 2) microbiological procedures, and 3) pharmacokinetic characteristics (published elsewhere, [11]).

## Methods

### Epidemiologic Investigation

This analysis included 328 protocol-correct participants in Study 28 [9]. Patients provided spot sputum specimens for AFB smear microscopy and culture, on both solid and liquid media, at baseline and weeks 2, 4, 6, and 8 of study therapy [9]. Thereafter spot sputa were obtained monthly until 2 consecutive specimens were culture-negative. Mycobacterial isolates from protocol-correct participants were confirmed to be *M. tuberculosis* complex and susceptible to isoniazid, fluoroquinolones, rifampin, and pyrazinamide. We defined culture conversion as the first negative sputum culture with at least one subsequent negative culture and no subsequent positive results.

We examined the probability of sputum culture conversion in liquid media using smoothed Kaplan-Meier plots, calculated at days 15, 29, 43, 57 after the start of therapy corresponding to weeks 2, 4, 6, 8, respectively. We investigated four stratified measures of disease extent at enrollment for African v. non-African patients. These stratified measures included: 1) Smear status normalized to the WHO standard (0–1+, 2+, 3+), 2) Radiographic extent of pulmonary involvement (<25%, 25%–50%, >50%) on baseline CXR, 3) aggregate cavity size on CXR (absent, <4 cm, ≥4 cm), and 4) the number of days-to-detection (DTD) of growth in liquid media for *M. tuberculosis* positive specimens (0–5 days, 6–9 days, 10+ days). The Wilcoxon test for paired samples was used to determine whether culture conversion curves were significantly different between African and non-African patients with equivalent measures of disease severity[12].

Information about smoking, diabetes, and self-designated race was obtained from participants through interview by study staff. HIV testing was performed on all study participants.

This work is a secondary analysis of data from TBTC Study 28 which was registered on ClinicalTrails.gov with the identifier NCT00144417. The protocol for this trial and supporting CONSORT checklist are available as supporting information; see Checklist S1 and Protocol S1. The protocol for TBTC Study 28 was approved by the CDC Human Research Protection Office and the 50 institutional review boards of the 27 sites enrolling patients. Patients enrolled in TBTC Study 28 provided written informed consent to participate in the study.

### Laboratory Methods and Investigation

Each enrolling site submitted sputum specimens to a local microbiology laboratory that participates in a quality assurance testing program for both smear and culture. Twenty-nine laboratories processed and cultured sputum for *M. tuberculosis* from TBTC Study 28 patients. Sputum smears were prepared from concentrated, decontaminated specimens and read using either the ATS/CDC[13] or WHO[14] smear grading systems and then harmonized to the WHO grading system for analysis. We reviewed laboratory practices and processes at these laboratories including media type used, decontamination procedures and inoculation volume. All laboratories used N-acetyl-L-cysteine (NALC)/sodium hydroxide (NaOH) for specimen decontamination.

The TBTC site in Uganda enrolled 55% of protocol-correct Study 28 patients of which many had delayed culture conversion so further investigation of the *M. tuberculosis* isolates from Uganda was undertaken. As a routine practice at this laboratory, all positive liquid culture tubes were subcultured on blood agar plates and stained using the Ziehl-Neelson method. Nucleic acid amplification of IS6110 was performed routinely on all AFB-positive culture tubes at enrollment to assess for *M. tuberculosis* complex. To assess for cross-contamination in the Ugandan laboratory and for potential re-infection with a different strain of *M. tuberculosis* a convenience sample of 21 pairs of baseline and 8-week isolates from Ugandan patients underwent spoligotyping[15], [16], MIRU[17] and/or RFLP[18] analysis at CDC.

## Results

Of the 328 protocol-correct patients in TBTC Study 28, 213 (65%) were enrolled in Africa, including 182 (55%) from Uganda and 31 (9%) from South Africa. Among the remainder, 19 (6%) were enrolled in Brazil, 12 (4%) in Spain, and 84 (26%) in North America.

**Figure 1:**
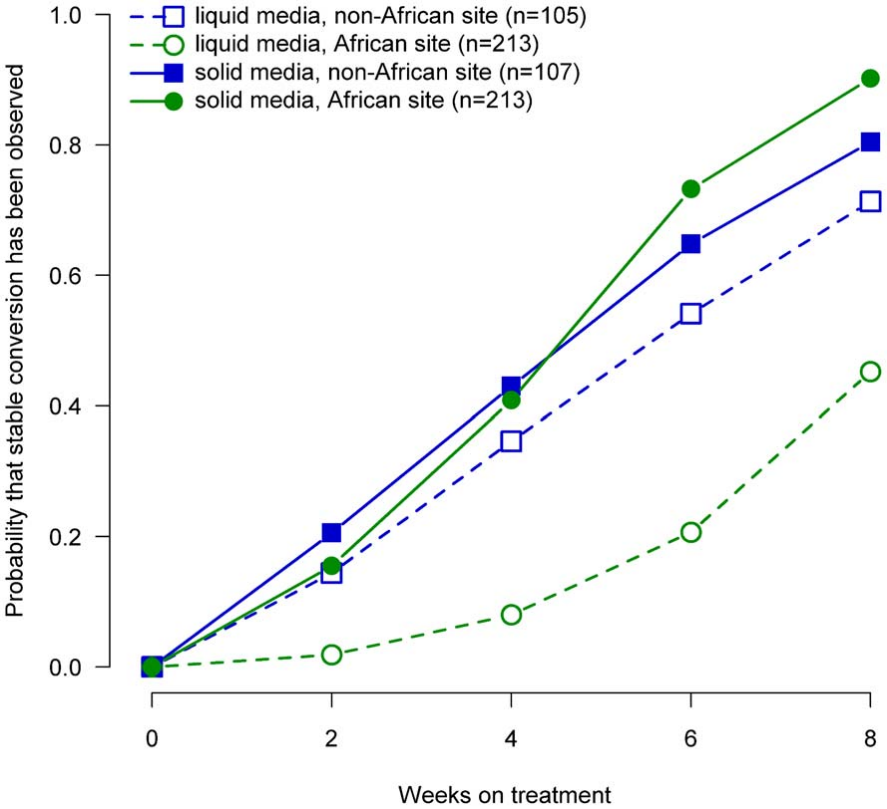
Probability of sputum culture conversion among TBTC Study 28 protocol-correct patients, by week of treatment, region (Africa vs. non-Africa) and culture media type (solid vs. liquid).

After 8 weeks of study therapy, sputa cultured in liquid media from African site patients were 29% less likely to have converted to negative than non-African patients (p<0.001; Wilcoxon test comparing the curves). Based on cultures on solid media, African site patients were 10% more likely to have converted than from non-African patients (p = 0.6; Wilcoxon test comparing the curves) (Figure 1). In Africa there was a 45% difference in the rate of conversion between solid and liquid media while there was only a 9% difference in non-African patients.

### Baseline Extent of Disease

At baseline African patients had a higher bacillary load based on sputum smear and a smaller number of days required for detection of *M. tuberculosis* growth in liquid media, compared to non-African patients (Table 1). Compared to non-African patients, a higher proportion of African patients had cavitary disease, and African patients had larger cavities and more lung fields involved by tuberculous lesions.

**Table 1:** Baseline measures of severity of disease, TBTC Study 28 protocol-correct patients, Africa vs. non-Africa.

**Smear Status** *	**0**–**1+**	**2+**	**3+**
Africa (N = 213)	24%	32%	44%
Non-Africa (N = 105)	51%	29%	20%
**Radiographic extent of involvement**	**<25%**	**25%**–**50%**	**>50%**
Africa (N = 213)	17%	50%	33%
Non-Africa (N = 105)	34%	37%	29%
**Cavitary size**	**Absent**	**<4 cm**	**≥4 cm**
Africa (N = 213)	23%	29%	48%
Non-Africa (n = 105)	32%	47%	21%
**Days to Detection** **	**10+**	**6**–**9**	**≤5**
Africa (N = 212)	26%	27%	47%
Non-Africa (N = 98)	41%	42%	17%

*using WHO criteria.

**in liquid media.

**Figure 2:**
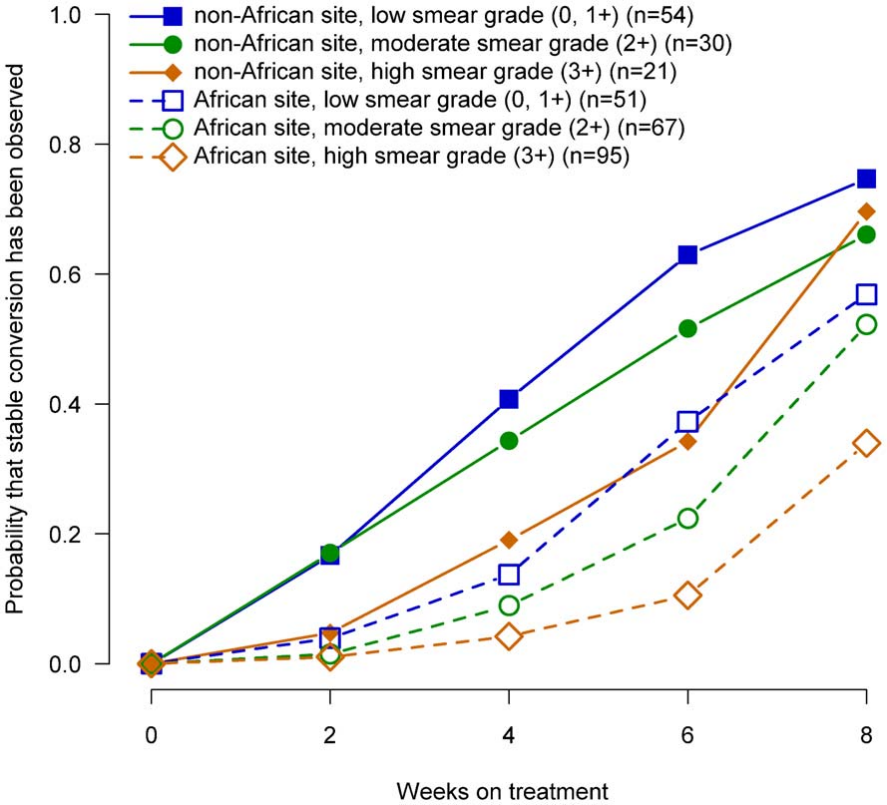
Probability of sputum culture conversion in liquid media by baseline sputum smear, African/non-African site and week of treatment, TBTC Study 28 protocol-correct patients.

**Figure 3:**
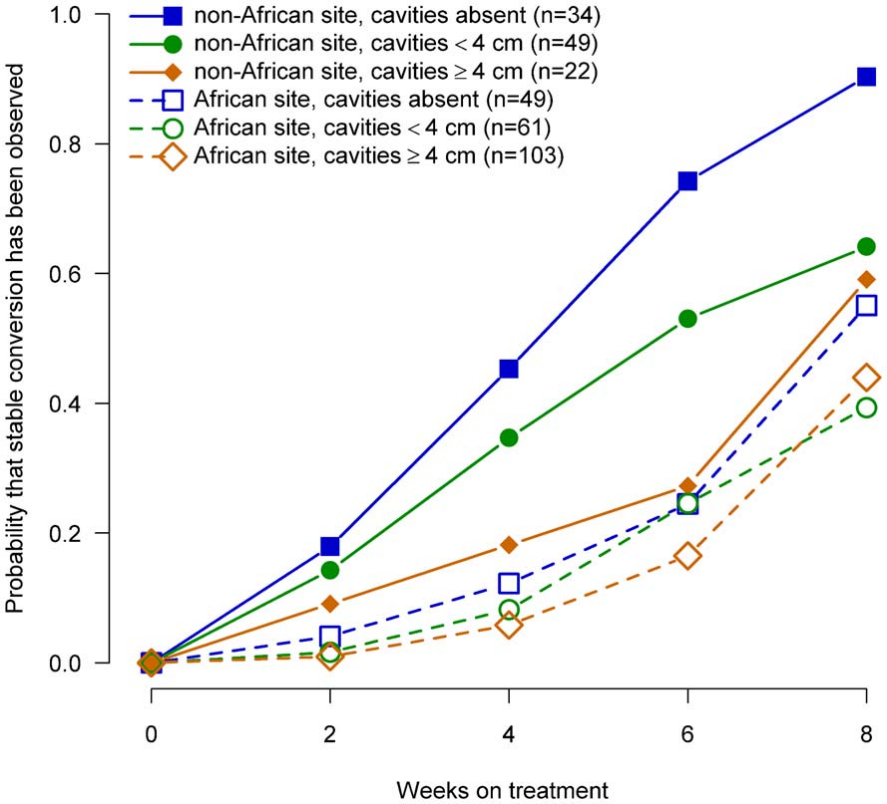
Probability of sputum culture conversion in liquid media by baseline aggregate cavity size, African/non-African site and week of treatment, TBTC Study 28 protocol-correct patients.

**Figure 4:**
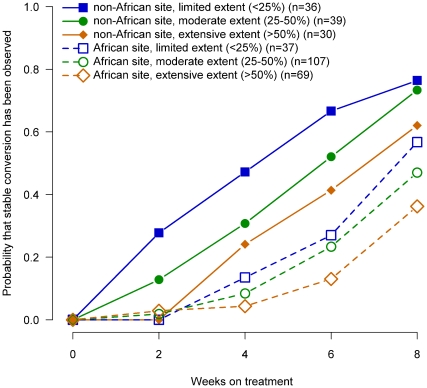
Probability of sputum culture conversion in liquid media by baseline radiographic extent of involvement, African/non-African site and week of treatment, TBTC Study 28 protocol-correct patients.

**Figure 5:**
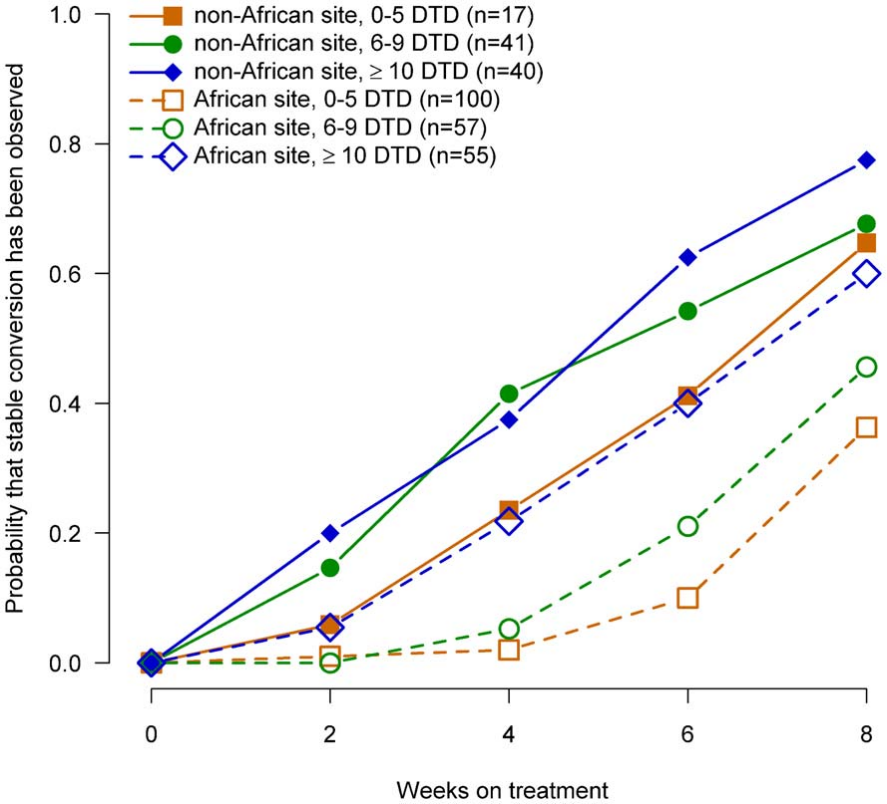
Probability of sputum culture conversion in liquid media by baseline number of days to detection (DTD) of growth in liquid media, African/non-African site and week of treatment, TBTC Study 28 protocol-correct patients.

To evaluate whether more severe disease in African patients explained the lower culture conversion rates in liquid media in African patients, we evaluated the probability of culture conversion by week of therapy for African and non-African patients stratified by severity of disease for baseline smear status (Figure 2), cavitary size (Figure 3), radiographic extent of disease (Figure 4) and the number of days to detect growth in liquid media (Figure 5).

Within each region (i.e., African, non-Africa) for each of these measures of extent of disease at enrollment, the relationship between severity and time to conversion was consistent and generally as expected. Patients with the most severe measures of disease had slower sputum culture conversion in liquid media compared to patients with moderate disease, who had slower conversion than patients with the least severe measures of disease.

However, for all four baseline measures of disease examined, when compared at equivalent levels of severity, African patients had substantially lower conversion rates in liquid media than non-African patients. In fact, African patients with the least severe measures of disease consistently had conversion rates similar to or lower than non-African patients with the most severe measure of disease. The curves for all 12 comparisons of the time to sputum culture conversion for equivalent measures of disease severity shown in figures 2, 3, 4, 5 were significantly different between African and non-African patients (See Table 2).

**Table 2:** Wilcoxon test P-values for the probability of sputum culture conversion in liquid media comparing African and non-African patients with equivalent baseline measures of disease severity during the first eight weeks of tuberculosis therapy as shown in figures 2, 3, 4, 5, TBTC Study 28.

	p-value
**Sputum Smear**	
low load (0, 1+)	0.001
moderate load (2+)	0.003
high load (3+)	<0.001
**Radiographic Extent of Involvement**	
limited extent (<25%)	0.001
moderate extent (25–50%)	<0.001
extensive (>50%)	0.001
**Aggregate Cavity Size**	
cavities absent	<0.001
cavities <4 cm	0.001
cavities >4 cm	0.045
**Number of Days to Detection in Liquid Media**	
10+ DTD	0.005
6–9 DTD	<0.001
0–5 DTD	0.010

**Figure 6:**
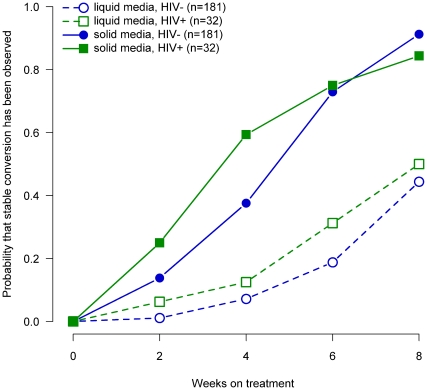
Probability of sputum culture conversion among African patients by HIV status by media type and week of treatment, TBTC Study 28 protocol-correct patients.

**Figure 7:**
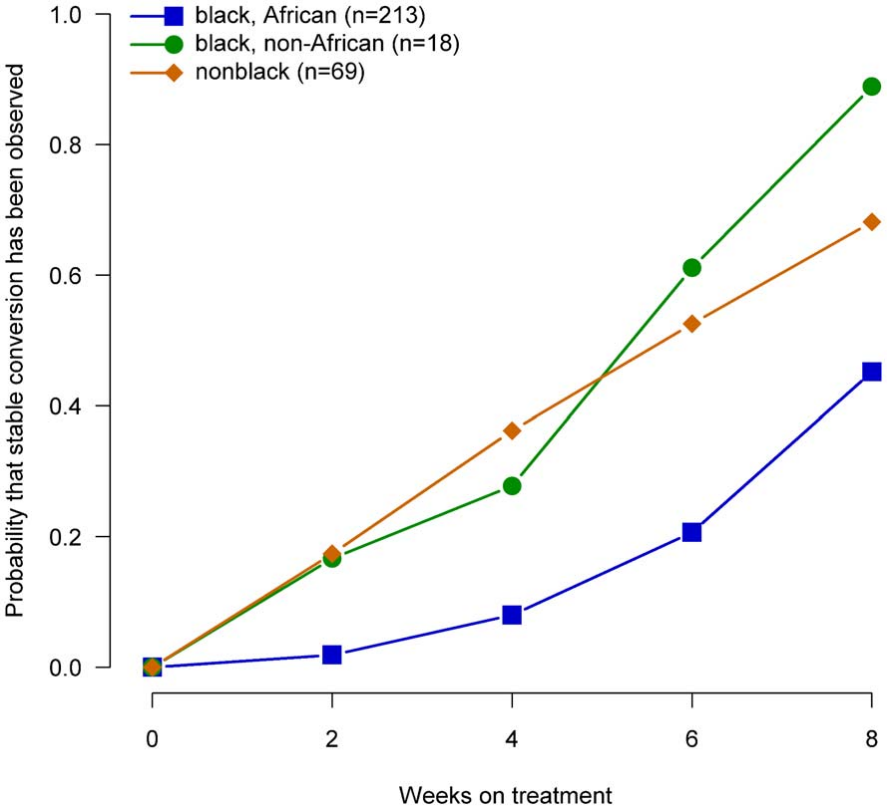
Probability of sputum culture conversion in liquid media by race (black and non-black), African/non-African site and week of treatment, TBTC Study 28 protocol-correct patients.

Compared with patients from non-African sites, those from African sites had lower prevalences of smoking (63% versus 27%, p>0.001) and of prior diagnosis of diabetes mellitus (17% versus 0%, p>0.001). African patients were significantly younger than non-African patients (29.4 years vs. 40.5 years, p<0.001). Of the 35 HIV-infected, protocol-correct patients, 32 were from African sites. Among African patients, HIV-positive patients had slightly higher conversion rates in liquid media compared to HIV-negative patients (Figure 6). Culture-conversion rates by race (black and non-black) were examined to determine if non-African patients of black race had lower conversion rates (Figure 7). At non-African sites the time to conversion in liquid media did not differ substantially between black (n = 26) and non-black patients (n = 80), but both were significantly lower than that in African patients (p<0.001, log-rank test). Therefore, smoking, diabetes, age, HIV status, and race did not explain lower sputum culture conversion rates in Africa.

### Laboratory Investigation

Among the 29 laboratories processing mycobacterial cultures for TBTC Study 28: 1) the final concentration of NaOH used for decontamination ranged from 1% to 3% (mean 1.6%, median 1.25%), Most of the non-African laboratories decontaminated with a final NaOH concentration of 2% while the two African laboratories processed specimens using a final NaOH concentration of 1% and 1.5%, 2) the median duration of decontamination was 15 minutes (range 15–20 min), 3) inoculation volume was 0.5 ml for liquid media for all but one site (See Table 3).

**Table 3:** Tuberculosis sputum specimen processing and culture practices from the highest enrolling sites of protocol-correct patients, TBTC Study 28.

Site	Number of Protocol-correct Patients Enrolled	Solid Media Type*	Liquid Media Type	Final Sodium Hydroxide Concentration used for specimen Decontamination	Decontamination Time (min)	Inoculation volume (ml) on liquid media
Uganda	182	7H10	MGIT (82%)BACTEC 460 (18%)***	1.5%	20	0.50
South Africa	31	7H11	MGIT	1.0%	15	0.50
						
Brazil	19	LJ	MGIT	2.0%	20	0.50
North Texas	13	LJ	MGIT	2.0%	15	0.50
South Texas	12	LJ	BACTEC 460 (75%)***MGIT (25%)	2.0%	15	0.50
Spain	12	LJ	MGIT (75%)MB/BacT (25%)	2.0%	15	0.50
Houston	8	LJ	MB/BacT	2.0%	15	0.50
Denver	7	7H11	MGIT	1.5%	15	0.50
New Jersey	7	7H11	MGIT	1.0%	20	0.50
Other non-African sites**	37	LJ or 7H11	MGIT (73%)BACTEC 460 (14%)***MB/BacT (13%)	1.0% –3.0%(median 1.0)	13–20(median 15)	0.50–0.70 (median 0.50

*If multiple solid media were inoculated at a site laboratory for analysis we used only one solid media result based upon media type. Solid media results were used in the following order of preference - LJ >7H11 >7H10.

**Represents 18 laboratories serving 15 study sites.

***Cultures in BACTEC 460 were read daily for the first 10 days and approximately weekly thereafter.

**Figure 8:**
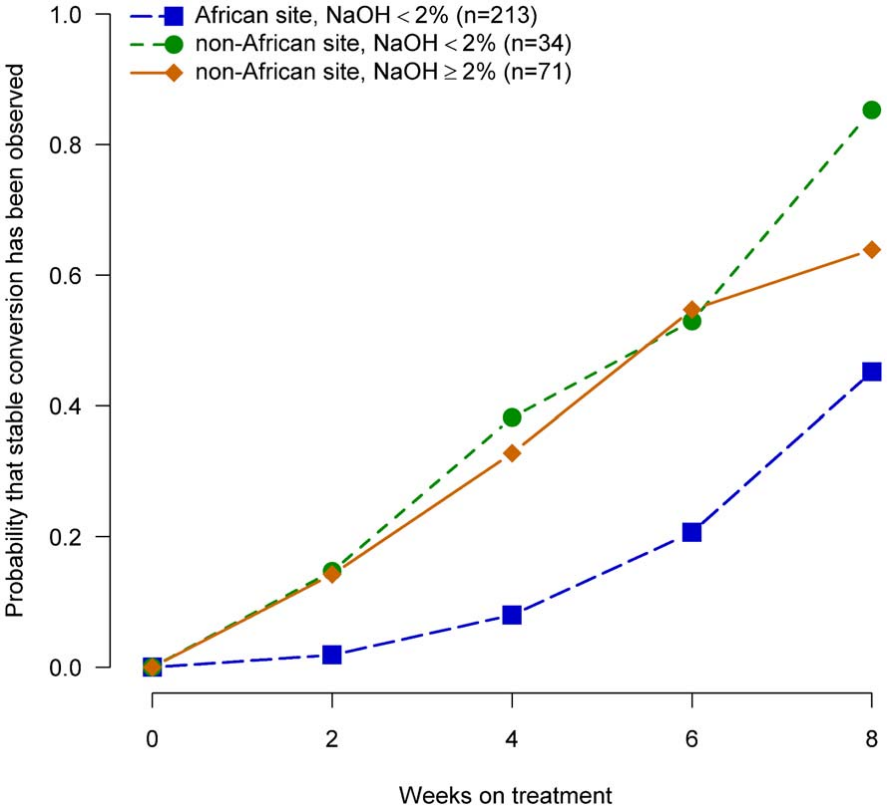
Probability of sputum culture conversion in liquid media by NaOH final concentration used for specimen decontamination, African/non-African site and week of treatment, TBTC Study 28 protocol-correct patients.

To assess whether differences in the final concentration of NaOH used in specimen decontamination might explain delayed conversion among African patients we examined the probability of culture conversion stratified by NaOH final concentration used in decontamination and geographic region of enrollment. We found that through week 6 of TB treatment non-African patients whose sputum specimens were decontaminated with NaOH concentrations <2.0% (range 1.0% –1.5%) had almost identical times to conversion as non-African patients with specimens decontaminated with NaOH concentrations ≥2.0% (range 2.0%–3.0%). However, at week 8 it was surprising to find that non-African patients whose sputum specimens were decontaminated with NaOH concentrations <2.0% had a higher rate of conversion than non-African patients with specimens decontaminated with NaOH concentrations ≥2.0% (Figure 8) suggesting that the NaOH concentration does not explain the observed delayed culture conversion for patients at African sites.

All isolates from Ugandan Study 28 protocol-correct patients were confirmed at enrollment and week-8 to be *M. tuberculosis* complex. All of the baseline and week 8 isolate pairs from 21 patients matched genotypically using RFLP, MIRU, and spoligotyping methods, suggesting that cross-contamination or re-infection did not explain the low culture negative conversion rate in liquid media among African patients.

## Discussion

Interest in geographic differences in response to TB chemotherapy dates back to 1956 when Fox et al. compared clinical, radiographic, and microbiologic outcomes in patients from Britain and Uganda [19]. In TBTC study 28 during the first 8 weeks of therapy we found later sputum culture conversion and lower rates of conversion in liquid media in African patients compared to non-African patients, despite African patients having comparatively higher conversion rates on solid media. African patients had more severe disease when their therapy was begun. However when we evaluated conversion rates stratified by varying levels of disease severity it became clear that our measures of baseline severity of disease did not explain the lower and delayed culture conversion in liquid media in African patients. In fact, African patients with the lowest severity of disease had conversion rates in liquid media that were similar to or lower than non-African patients with the highest severity of disease.

Some studies suggest that smoking [7], [8], diabetes [3], [4], [5], increasing age [20] and HIV positive status [6] are associated with delayed conversion. In Study 28, African patients had a lower prevalence of smoking and diabetes and were younger compared to non-African patients, so these conditions did not appear to contribute to the lower conversion rates in Africa. Patients with HIV infection actually had slightly higher conversion rates in liquid media compared to HIV-negative patients.

In Study 28 the administration of all doses of anti-TB drugs were directly observed. In an intensive pharmacokinetic sub-study performed on a convenience sample of 72 patients enrolled in TBTC Studies 27 and 28 (37 African and 35 non-African), the mean AUC_0-24_ for rifampin and moxifloxacin did not differ between African and non-African patients [11]. Isoniazid levels were not examined. We did not find a pharmacokinetic reason for lower conversion rates among Africans.

A panel of experienced mycobacteriologists, clinicians, and epidemiologists reviewed laboratory processes in the context of the site-specific conversion rates and the number of patients represented. They determined that the laboratories were operating within accepted guidelines for conducting mycobacterial cultures[21]. We found some variation in laboratory processes (e.g., decontamination procedures) that might have led to differences in the rates of culture conversion. In particular, the concentration of NaOH used during specimen decontamination was generally lower at African sites (albeit with longer time for decontamination). Laboratory studies done on sputum seeded with bacteria and *M. tuberculosis* H37Ra revealed that decontamination with NALC-NaOH with final concentrations of 1–2% NaOH and decontamination treatment times of up to 30 minutes did not affect the viability of *M. tuberculosis* when grown on solid media [22]. However a study of patient sputum specimens collected prior to initiation of TB therapy suggested that relatively small increases in the final NaOH concentration used in decontamination from 1% to 1.25% significantly decreased the recovery of *M. tuberculosis* on a solid media [23]. When we evaluated the effect of the NaOH concentration on liquid culture results after the initiation of TB therapy, stratified by geographic region, we found that the NaOH concentration did not explain delayed conversion in African patients compared to those at non-African sites.

At baseline, prior to the initiation of therapy, solid and liquid media performed equally well for isolating *M. tuberculosis* from the sputa of the smear-positive patients enrolled in Study 28. As therapy progressed, at both African and non-African sites, patients had lower conversion rates in liquid media compared to solid media. This is expected, as liquid media allow for growth of some *M. tuberculosis* organisms that are unable to grow on solid media[24]. Unexpectedly, the difference in conversion rates between solid and liquid media was much greater in Africa. This finding of large differences in the yield of solid and liquid media for *M. tuberculosis* for African patients receiving therapy is not unique to TBTC Study 28. A similar clinical trial conducted in South Africa by the OFLOTUB group found that at 8 weeks of TB therapy the proportion of sputum cultures that were negative on solid media (7H11) was twice that found on liquid media (MGIT)[25]. Joloba et. al. studied sputum culture conversion on both solid (7H10 selective) and liquid media (BACTEC 460) during the course of TB therapy for both HIV infected and uninfected populations. They also found that conversion in liquid media was lower and substantially delayed compared to that of solid media for both populations[26].

Regional differences in outcomes of TB therapy are not unique to TBTC studies. In a recent phase 3 trial conducted in Brazil, the Philippines and Uganda examining treatment shortening for HIV negative adults with non-cavitary TB and negative 2-month sputum culture on solid media, patients from Uganda were more likely to relapse than participants from Brazil or the Philippines[27]. This study was stopped early because patients in the 4-month treatment arm had significantly more relapse (13 relapses among 196 patients) than those in the 6-month treatment arm (3 of 198). Among the 16 relapses, 12 (75%) occurred in the Ugandan patients and 4 (25%) in Brazilian patients. Initial sputum smear grade and the number of lung zones involved by tuberculous lesions were greater among patients enrolled in Uganda. In the multivariate analysis, enrollment at the Uganda site was an independent risk factor for relapse after accounting for baseline sputum smear and radiographic extent of disease. Two-month sputum culture results in liquid media were not reported in that study.

Recent TB trials illustrate important advantages and disadvantages of different approaches to phase 2 clinical trials [9], [10], [25], [27], [28]. Multicenter international studies are likely to enroll more rapidly and to be more representative and easier to generalize, they also allow examination for regional differences in response to therapy. However, the potential influence that variability in laboratory or other procedures might have on the interpretation of clinical trial results must be considered. While laboratories engaged in clinical trials should meet recognized clinical diagnostic standards for culturing mycobacteria, the degree of heterogeneity of procedures allowed to meet these routine clinical diagnostic standards may not be sufficient for early phase controlled clinical trials. Further research is needed to determine whether and how modest differences in processing and culturing of sputa influence microbiological outcomes over the course of TB therapy in phase 2 studies. Such studies should focus on determining the principle processes that contribute to variability in microbiological outcomes measured during clinical trials. Laboratory processes that diminish the sensitivity or specificity of microbial biomarkers of treatment outcomes (e.g., 8-week culture conversion) will influence the ability of both single site and multi-site studies to determine the relative efficacy of drug regimens. Efforts to optimize the performance characteristics of microbiological tests in predicting treatment failure and relapse are needed if we hope to make good decisions regarding which drugs and regimens to move forward into phase 3 clinical trials. If, after investigation, variations in laboratory practices are not found to explain geographic difference in time to culture conversion, then alternative explanations (e.g., differences in socioeconomic/nutritional status, host immunity, or microbial pathogenesis) should be explored.

## Supporting Information

Checklist S1 (JPG)

Protocol S1 (DOC)
