# Supplementary figures and images for: Geographic Differences in Time to Culture Conversion in Liquid Media: Tuberculosis Trials Consortium Study 28. Culture Conversion Is Delayed in Africa
Source: PLoS One. 2011 Apr 11;6(4):e18358. doi: 10.1371/journal.pone.0018358 (PMC3073969; doi:10.1371/journal.pone.0018358)

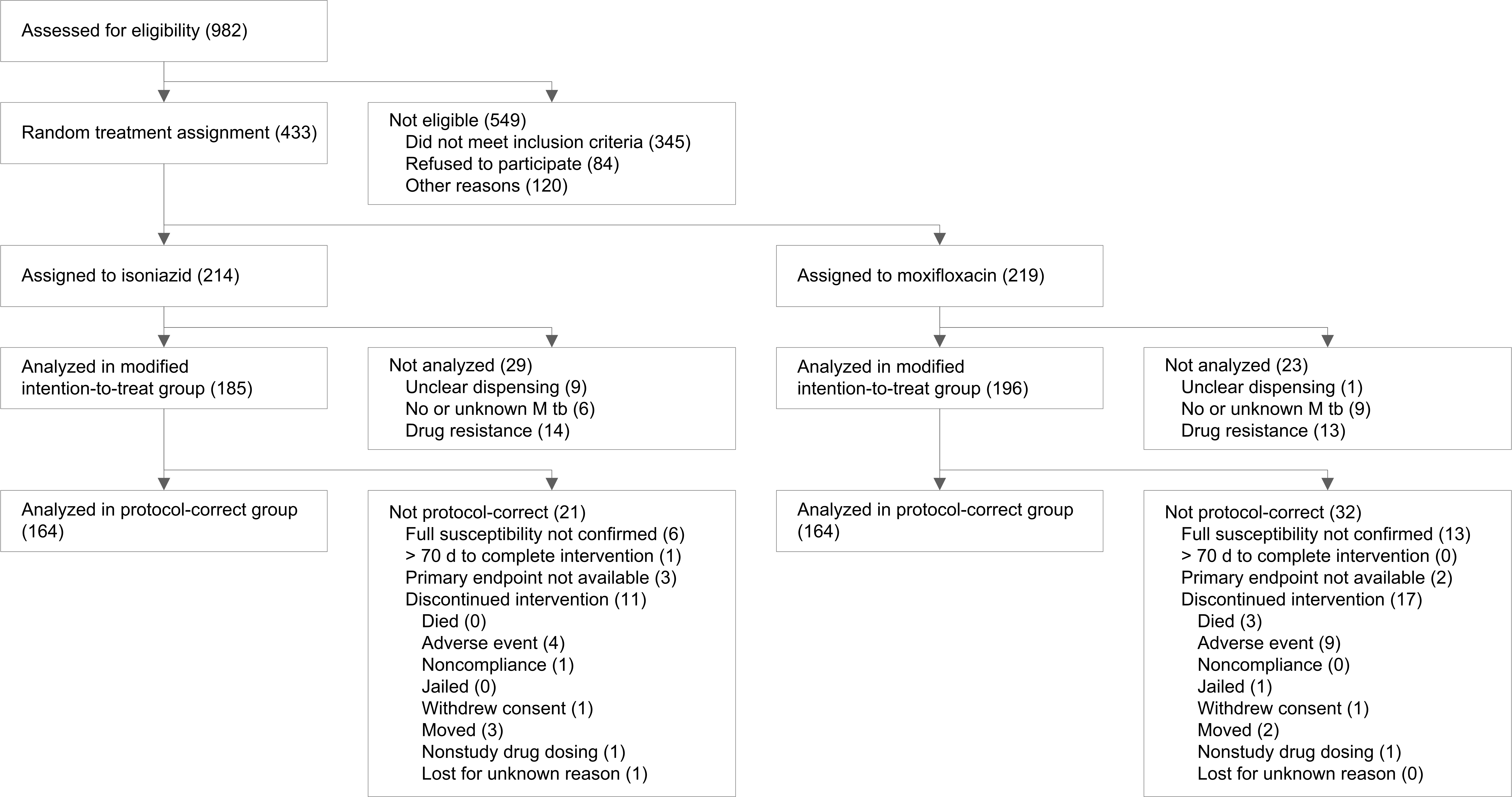

Supplement: Checklist S1 — (JPG) [file pone.0018358.s001.jpg]
