# Supplementary material for: Geographic Differences in Time to Culture Conversion in Liquid Media: Tuberculosis Trials Consortium Study 28. Culture Conversion Is Delayed in Africa
Source: PLoS One. 2011 Apr 11;6(4):e18358. doi: 10.1371/journal.pone.0018358 (PMC3073969; doi:10.1371/journal.pone.0018358)
Supplement: Protocol S1 — (DOC) [file pone.0018358.s002.doc]

# Evaluation of a Moxifloxacin-Based, Isoniazid-Sparing Regimen for Tuberculosis Treatment

#

# TBTC Study 28

Protocol chair: Richard E. Chaisson MD – Johns Hopkins University

Protocol Team

Susan E. Dorman, M.D. – Johns Hopkins University, Co-chair

John L. Johnson, M.D. – Case Western Reserve University, Co-chair

Eric L. Nuermberger, M.D. – Johns Hopkins University

John Bernardo, M.D. – Boston University School of Medicine

Dick Menzies, M.D. – McGill University

Elizabeth Guy, M.D. – Baylor College of Medicine

Nesri Padayatchi, M.D. – Natal University

Maria Corazon Leus, R.N. – University of Medicine and Dentistry of New Jersey

Gina Maltas, R.N. – Johns Hopkins University

Lorna Bozeman, MS – CDC

Nong Shang, Ph.D. – CDC

Priya Guyadeen - Westat

Jacques H. Grosset, M.D. – Johns Hopkins University

Shurjeel Choudhri, MD – Bayer Pharmaceuticals

Stefan Goldberg, MD – CDC, project officer

# TABLE OF CONTENTS

# PROTOCOL SYNOPSIS 4

1. **INTRODUCTION** 8
   1. Background and Rationale 8
   2. Study Drugs 19

# METHODOLOGY 28

- 1. Study Design – 4-drug phase 28
  2. Continuation Phase Therapy 29
  3. Primary Objectives 30
  4. Secondary Objectives 30
  5. Study Endpoints 30
  6. Randomization 31
  7. Sample Size Calculation 31
  8. Patient Selection 31
     1. Inclusion Criteria 32
     2. Exclusion Criteria 33

1. **STUDY PLAN** 34
   1. Study Procedures 34
      1. Screening Visit 34
      2. Randomization Visit 34
      3. Study visits – intensive phase of therapy 35
      4. Study visits – continuation phase of therapy 35
      5. Scheduled unblinding of study treatment regimens 36
      6. Compensation for study subjects…………………………….……..36
2. **CLINICAL MANAGEMENT ISSUES** 36
   1. Drug-Susceptibility Testing 36
   2. Management of patients with baseline drug-resistant isolates 37
   3. Study drug toxicity 38
   4. Adverse event reporting 39
   5. Management of adverse events 39
   6. Criteria for discontinuation of study drugs 40
   7. Criteria for temporary discontinuation of study therapy 41
   8. Criteria for permanent discontinuation of study therapy 41
   9. Unscheduled unblinding of study assignment 41
   10. Concomitant medications during study phase 42
   11. HIV management 43
   12. Management of patients with tuberculosis treatment failure………….44
3. **EVALUATION** 45
   1. Data analysis 45
4. **ADMINISTRATIVE ISSUES** 46
   1. Supply of study drugs 46
   2. Drug accountability and record keeping 46
5. **TBTC STUDY SITES** 46
6. **HUMAN SUBJECTS PROTECTION** 47
   1. Ethical issues in doing this trial in developing and developed countries 47
   2. Ethical issues involving persons who are incarcerated 47
   3. Institutional review board involvement 48
7. **REFERENCES** 49
8. **Appendix A** – Sample informed consent 58
9. **Appendix B** – Karnofsky performance scale 65
10. **Appendix C** – Clinically significant drug-drug interactions involving

rifamycins…………………………………………………………………….…66

14. **Appendix D –** Time events schedule 68

15. **Appendix E –** National Cancer Institute Common Toxicity Criteria…………..70

# PROTOCOL SYNOPSIS

The primary objective of this Phase 2 clinical trial is to compare the safety and antimicrobial activity of a moxifloxacin-containing regimen (moxifloxacin, rifampin, pyrazinamide, ethambutol [MRZE]) in which moxifloxacin has been substituted for isoniazid, to the standard control regimen (isoniazid, rifampin, pyrazinamide, ethambutol [HRZE]) in the first two months of treatment of sputum smear-positive pulmonary tuberculosis. The assessment of antimicrobial activity will be sputum culture-conversion. Higher rates of sputum culture conversion after 2 months of treatment with a moxifloxacin-containing regimen would support Phase 3 clinical trials of moxifloxacin in treatment regimens of less than the current 6 month standard regimens.

The specific aims of this study are to:

Primary To compare the culture-conversion rates at the end of the intensive phase of therapy of the moxifloxacin regimen vs. that of the isoniazid regimen

Secondary To compare the safety and tolerability of the moxifloxacin regimen to that of the isoniazid regimen

To determine the time to culture-conversion of the moxifloxacin regimen and the isoniazid regimen, using data from 2-, 4-, 6-, and 8-week cultures.

To compare the proportion of patients with any Grade 3 or 4 adverse reactions

To compare adverse events and 2-month culture conversion rate among HIV-infected patients vs. HIV-uninfected patients

To compare the rates of treatment failure of the moxifloxacin regimen to the isoniazid regimen

To determine whether there is delayed toxicity attributable to moxifloxacin (toxicity that becomes evident after the 2 months of moxifloxacin therapy)

Rationale – Current treatment of smear positive pulmonary tuberculosis requires a minimum of 6 months, a treatment duration that is challenging for patients and tuberculosis control programs. Therefore, a high priority in tuberculosis research is the identification of agents that can shorten treatment. Several fluoroquinolone antibiotics have potent activity against *Mycobacterium tuberculosis* *(M. tuberculosis)* in preclinical testing. Of the currently available fluoroquinolones, moxifloxacin has excellent activity *in vitro* and in animal models of tuberculosis, a favorable pharmacokinetic profile (serum half-life of 10-12 hours), lack of problematic drug-drug interactions, no need for dosage adjustment for renal and hepatic insufficiency, and an excellent safety profile. In addition, in the murine model of tuberculosis, the substitution of moxifloxacin for isoniazid resulted in significant reductions in the time to culture conversion and the time to sterilization when compared to the standard combination rifampin, isoniazid and pyrazinamide. However, moxifloxacin has not been fully evaluated in humans for tuberculosis treatment. There is a need to assess not only the anti-tuberculosis activity of moxifloxacin-containing regimens, but also the safety of more prolonged therapy with moxifloxacin.

Two-month culture conversion rates are a well-accepted surrogate marker for the sterilizing activity of anti-tuberculosis drugs. Rifampin and pyrazinamide, the key drugs in current 6-month regimens, markedly increase 2-month culture-conversion rates. Therefore, this study will use 2-month culture conversion rate as the measure of antimicrobial activity of moxifloxacin.

# Study Design

This study will be a multicenter, placebo-controlled, double-blind trial evaluating the effect of using moxifloxacin (M) in place of isoniazid (H), in combination with rifampin (R), pyrazinamide (Z) and ethambutol (E) on 2-month culture conversion rates among patients with sputum smear-positive pulmonary tuberculosis. The protocol will enroll HIV-infected and uninfected subjects at Tuberculosis Trials Consortium (TBTC) sites. Subjects who meet the inclusion criteria will be randomized to one of the following two arms:

Intensive Phase Study Regimens

| **Study Regimen** | **Intensive phase (first 8 weeks of anti-TB therapy)** | |
| --- | --- | --- |
| Control regimen | HRZE daily* (+ moxifloxacin placebo)† |  |
| Moxifloxacin regimen | MRZE daily* (+ isoniazid placebo) † |  |

H-isoniazid; R-rifampin; Z-pyrazinamide; E-ethambutol; M-moxifloxacin

* Daily therapy will be 5 days per week. However, at the discretion of the investigator, patients in either study arm may be given therapy 7 days per week during the first two weeks of the study (only 5 doses will be counted toward completion of the four-drug intensive phase of therapy).

† Vitamin B6 (50 mg) will be given with each dose of therapy

After completion of intensive phase therapy, patients in all study arms will then be treated for 18 weeks (30 weeks for those with cavitation on initial chest radiograph plus a positive sputum culture for *M. tuberculosis* at 2 months of therapy) with an ATS/IDSA/CDC-recommended continuation phase regimen.

The moxifloxacin dose will be the currently licensed dose of 400 mg. Because cavitation at baseline (time of diagnosis) is associated with a substantially decreased rate of 2-month culture conversion (67% vs. 85% in TBTC Study 22), randomization will be stratified by presence of cavitation. In addition, randomization will be stratified by geographic continent.

# Inclusion criteria

1. Suspected pulmonary tuberculosis with acid-fast bacilli in a stained sputum smear. Patients whose sputum cultures do not grow *M. tuberculosis*, and those having an *M. tuberculosis* isolate resistant to (one or more) isoniazid, rifampin, pyrazinamide, or fluoroquinolones will be discontinued from the study, but followed for 14 days to detect late toxicities from study therapy. Patients having extra-pulmonary manifestations of tuberculosis, in addition to smear-positive pulmonary disease, are eligible for enrollment.
2. Willingness to have HIV testing performed, if HIV serostatus is not known or if the last documented negative HIV test was more than 6 months prior to enrollment
3. 7 (seven) or fewer days of multidrug therapy for tuberculosis disease in the 6 months preceding enrollment
4. 7 (seven) or fewer days of treatment with a fluoroquinolone during the 3 months preceding enrollment
5. Age > 18 years
6. Karnofsky score of at least 60 (requires occasional assistance but is able to care for most of his/her needs)
7. Signed informed consent
8. Women with child bearing potential must agree to practice an adequate (barrier) method of birth control or to abstain from heterosexual intercourse during study therapy.
9. Laboratory parameters done at, or ≤ 14 days prior to, screening:

- Serum amino aspartate transferase (AST) activity ≤ 3 times the upper limit of normal
- Serum total bilirubin level ≤ 2.5 times the upper limit of normal
- Serum creatinine level ≤ 2 times the upper limit of normal
- Complete blood count with hemoglobin level of at least 7.0 g/dL
- Complete blood count with platelet count of at least 50,000/mm^3^
- Serum potassium ≥ 3.5 meq/L
- Negative pregnancy test (for women of childbearing potential)

### Exclusion criteria

1. Breast-feeding
2. Known intolerance to any of the study drugs
3. Known allergy to any fluoroquinolone antibiotic
4. Concomitant disorders or conditions for which moxifloxacin, isoniazid, rifampin, pyrazinamide, or ethambutol are contraindicated. These include severe hepatic damage, acute liver disease of any cause, and acute uncontrolled gout.
5. Current or planned therapy during the intensive phase of tuberculosis treatment using drugs having unacceptable interactions with rifampin (rifabutin can be substituted for rifampin during the continuation phase of therapy)
6. Current or planned antiretroviral therapy during the intensive phase of tuberculosis treatment
7. History of prolonged QT syndrome or current or planned therapy with quinidine, procainamide, amiodarone, sotalol, disopyramide, ziprasidone, or terfenadine during the intensive phase of tuberculosis treatment.
8. Pulmonary silicosis
9. Central nervous system TB

Primary endpoints

1. The proportion of patients having a negative sputum culture at 2 months of therapy
2. The proportion of patients who discontinue assigned study therapy for any reason during the first 2 months

Study Management

Patients will have study visits every two weeks during the first 2 months of therapy. At each study visit, there will be a clinical assessment for toxicity and a sputum specimen will be obtained for smear and culture (two sputum specimens will be obtained after 8-weeks). Mycobacterial cultures must include an approved broth culture system, supplemented by at least one solid medium. Patients who are unable to produce an expectorated sputum specimen at weeks 2, 4, 6, or 8 will have sputum induction.

Specific monitoring will be performed for retinal toxicity, which is a recognized side effect of ethambutol and has been observed in some animal species after prolonged dosing of moxifloxacin (though not reported in clinical trials or post-marketing surveillance of moxifloxacin). Monitoring for retinal toxicity will consist of visual acuity and color vision testing per standard site practice. Laboratory monitoring will consist of a CBC, serum AST, bilirubin, and creatinine, and will be obtained at 2, 4, 6, 8, and 12 weeks. Because significant cardiotoxicity has not been observed among the over 10 million patients treated with moxifloxacin after its approval and is not associated with the duration of treatment in animal models, electrocardiographic monitoring will not be done during this study.

## Duration of follow-up

The study will end upon completion of 18 weeks (or 30 weeks for individuals with cavitation on initial chest radiograph plus positive sputum culture at 2 months of treatment) of an ATS/IDSA/CDC continuation phase therapy regimen and a related study visit.

Sample Size and Power

The culture conversion rate at 8 weeks among patients with initial sputum smear

positivity treated with HRZE who enrolled into TBTC Study 22 was 73.6% (419/574).

We hypothesize that the effects of using a fluoroquinolone in place of isoniazid will

improve the potency of the intensive phase of therapy by approximately that of the

addition of pyrazinamide (mean of 12.7%, see Table 7). To detect an increase in

culture conversion from 75% to 88% with a two-sided test at the 0.05 level with 80%

power requires 154 patients per group. We will increase this by 25% to compensate for

missing cultures, drug resistance, etc. Therefore there will be 205 patients in each of

the two arms, 410 patients in the entire study. If, near the culmination of the intended enrollment of 410 patients to achieve 308 evaluable patients, it is determined that patient dropout, missing cultures, etc has exceeded the 25% estimate, then a proportionate number of additional patients will be recruited to retain statistical power.

1. **INTRODUCTION**
   1. Background and Rationale

**Tuberculosis as a global health problem**

Tuberculosis (TB) is one of the most important global health problems. According to recent estimates from the World Health Organization, 8 million new cases of TB and 1.9 million deaths from TB occur annually, making TB the second leading cause of death from an infectious pathogen, exceeded only by HIV/AIDS[1]. With the recent decline of TB rates in the United States, the goal of eliminating TB in the U.S. has become increasingly focused on developing better strategies to treat *M. tuberculosis* disease. More than 90% of all tuberculosis cases and 98% of all deaths due to tuberculosis occur in developing countries[2].  Up to 7% of all deaths and 26% of preventable deaths in developing countries are due to tuberculosis[3, 4].  Tuberculosis predominantly affects young adults in their most productive years of life and has substantial impact on economic development.

# The need for new drugs for treatment of tuberculosis

New tuberculosis drugs are needed for four reasons: 1) to shorten and/or simplify treatment of drug susceptible tuberculosis, 2) to improve the treatment of patients with multi-drug resistant tuberculosis (MDR TB), 3) to provide effective therapy for patients intolerant to the current first-line drugs, and 4) provide for short, effective treatment of patients with latent tuberculosis infection (LTBI)[5]. Although highly effective regimens have been developed for the treatment of patients with drug-susceptible tuberculosis, they must be administered for a minimum of six months to achieve optimal results[6]. Non-adherence with the relatively lengthy course of treatment remains a major problem. To address this, directly observed therapy (DOT) has been promulgated as a standard of care worldwide. However, the administrative burden of providing DOT for all patients is considerable. Thus, new drugs that would permit significant shortening of treatment are urgently needed, as are drugs that could enable effective treatment being given at widely-spaced intervals of one week or more.

Although rates of MDR TB are declining in the United States, this problem appears to be growing in a number of countries throughout the world. At present, successful treatment of MDR TB requires drugs that are less effective, more toxic, and more expensive than those used for standard treatment. Moreover, MDR TB treatment regimens often have to be given daily for 18-24 months. Although new drugs that are effective against MDR TB organisms would alone not solve the MDR TB problem, their judicious use would greatly improve the treatment for many patients.

Finally, the United States and several other low-incidence countries have embarked on plans to eliminate tuberculosis[7]. An important component of an elimination strategy is the identification and treatment of high-risk persons with LTBI. However, the most commonly used LTBI treatment regimen is isoniazid given for nine months, and adherence with this regimen is a major limitation. Although a shorter LTBI treatment regimen with rifampin and pyrazinamide appears to be effective[8], toxicity has recently been found to be unacceptably high[9]. Thus, new drugs to provide for safe and effective “short-course” LTBI treatment is a major need in countries like the U.S.

Fluoroquinolone antibiotics are active against most strains of *M. tuberculosis* (Table 1) and, despite the absence of efficacy data from controlled clinical trials, have gained general acceptance for the treatment of MDR TB. Recent experimental and clinical data suggests that these agents may be potent sterilizing agents that could shorten regimens for the treatment of active tuberculosis and be effective against LTBI. Thus, newer fluoroquinolones, such as moxifloxacin, have the potential to achieve all four objectives of a new tuberculosis drug.

**Table 1.** Fluoroquinolone (ciprofloxacin) resistance among *M. tuberculosis* isolates of patients enrolled in Tuberculosis Trials Consortium (TBTC) trials or isolates referred from local public health laboratories to the Mycobacteriology Laboratory at the Centers for Disease Control and Prevention, 1996-2000[10].

| *Pattern of resistance* | *Number (%)* | *Number having ciprofloxacin-resistance (%)* |
| --- | --- | --- |
| Susceptible to all first-line drugs | 1790 | 2 (0.1) |
| Resistant to at least one drug, but not isoniazid plus rifampin | 880 | 7 (0.8) |
| Resistant to isoniazid and rifampin | 603 | 25 (4.1) |
| Total | 3273 | 33 (1.0) |

**Preclinical studies of the activity of moxifloxacin against *Mycobacterium tuberculosis***

Moxifloxacin is a new fluoroquinolone antibiotic with excellent activity against *M. tuberculosis* both *in vitro* and in animal models. In a limited study using the agar proportion method on 7H10 medium, Woodcock et al. found that the minimal inhibitory concentrations (MIC) for moxifloxacin ranged from 0.12 to 0.5 µg/ml for four clinical isolates of *M. tuberculosis*, three of which were drug-resistant (to HS, HR, and S)[11]. Gillespie and Billington determined the MICs for 19 strains of *M. tuberculosis* to moxifloxacin, ciprofloxacin, levofloxacin, and sparfloxacin using the proportion method in 7H10 agar[12]. Moxifloxacin was the most active of the drugs tested (Table 2). Rodriguez and others conducted a similar evaluation against 55 clinical tuberculosis isolates from untreated patients with similar findings[13]. In another recent study from Italy, the activity of moxifloxacin was tested against 86 *M. tuberculosis* strains including 13 resistant and 4 MDR strains. All but two strains were susceptible to moxifloxacin at 0.5 µg/ml[14].

**Table 2.** Minimal inhibitory concentrations (MIC) (µg/ml) of several fluoroquinolones determined on 7H10 medium by the proportion method[12]

| *Drug* | *MIC range* | *MIC_50_* | *MIC_90_* |
| --- | --- | --- | --- |
| Moxifloxacin | 0.06->0.25 | 0.12 | 0.25 |
| Levofloxacin | 0.25-1.0 | 0.5 | >0.25 |
| Sparfloxacin | <0.06->0.25 | 0.12 | >0.25 |
| Ciprofloxacin | <0.25-1 | 0.5 | 0.5 |

Ji and others evaluated the *in* vitro and *in vivo* activities of moxifloxacin, clinafloxacin, and sparfloxacin[15]. The MIC_90_ determined on 7H11 medium by the proportion method of sparfloxacin and moxifloxacin were similar and slightly lower than that for clinafloxacin. In the experimental study, groups of 30 female Swiss mice each were infected by the intravenous route with 6.2 x 10^6^ colony forming units (CFU) of *M. tuberculosis* H37Rv strain and treated six times weekly for four weeks with various drug dosages beginning one day after infection. Moxifloxacin was the most active of the three fluoroquinolones tested, at the 100 mg/kg dosage comparable to that of isoniazid (Figure 1). This was the first study to demonstrate the significant activity of this drug in an animal model.

**Figure 1.** Activities of fluoroquinolones against M. tuberculosis in mice infected intravenously with *M. tuberculosis* H37Rv[15]

Isoniazid

Moxifloxacin

Sparfloxacin

Clinafloxacin

Miyazaki et al. evaluated moxifloxacin and isoniazid in 5 week old female Swiss mice given a non-lethal IV infection with the CDC1551 strain of *M. tuberculosis*, with groups of eight mice each treated six times weekly beginning one week after infection for 4 and 8 weeks (in the initial study) and beginning one day after infection for 3 and 7 weeks (in a second study)[16]. In the first experiment where moxifloxacin was dosed at 100 mg/kg, the drug had activity comparable to isoniazid, and there was a suggestion of an additive effect from the two drugs combined.

Because pharmacokinetic data suggest that a dose of 400mg/kg in the mouse more closely approximated the area under the curve (AUC)-time curve levels in man following the recommended dose of 400 mg, Yoshimatsu and colleagues assessed the bactericidal activity of the drug in doses up to 400 mg/kg given daily or weekly for 28 days to mice infected intravenously with 5 x 10^6^ CFU of *M. tuberculosis*. Dose-dependent reductions in CFU counts in splenic homogenates were seen for daily moxifloxacin therapy. The maximum bactericidal effect was seen with daily doses of 400 mg/kg, which resulted in a reduction in CFU counts of 1 log(10) greater than that with INH treatment, although the difference was not statistically significant. No significant activity was seen with once-weekly dosing[17].

In another published experimental study, the long-term activity of moxifloxacin in multi-drug therapy was assessed. In this study that was the third of a series of studies intended primarily to evaluate a variety of rifapentine-based regimens given once weekly, moxifloxacin (also dosed once weekly) had considerable activity, with the suggestion that the drug would be an excellent companion drug to rifapentine[18]. Following intravenous infection with 1.6x10^7^ CFU of H37Rv, groups of 40-60 mice (to provide for 20 mice in each treatment arm at each sacrifice period) were treated with one of four different rifapentine regimens each beginning after two weeks of daily treatment, and one fully intermittent regimen. Three control groups were included: untreated, rifapentine alone, and an active control of standard therapy. All regimens were given for 6 months, and mice were then followed for 3 months for relapse. The regimen of two weeks of daily isoniazid (H), rifampin (R), pyrazinamide (Z), and moxifloxacin (M) followed by 5.5 months of once weekly rifapentine (P) plus HM gave results comparable to those achieved with standard treatment (2HRZ/4HR) (Table 3).

**Table 3.** Activity in the mouse model of once-weekly regimens including rifapentine and moxifloxacin during and after 6 months of treatment[18]

| Regimen | CFU, 2 weeks | # culture-positive / total (%) | |
| --- | --- | --- | --- |
|  |  | 6 months | 9 months |
| 6P_1/7_ | ND | 7/10 (70%) * | ND |
| 2HRZ_5/7_ / 4HR_5/7_ | 5.60 + 0.34 | 0/20 | 1/16 (6.3%) |
| 0.5SHRZ_5/7_ / 5.5SHP_1/7_ | 4.94 + 0.32 | 2/16 (12.5%) | 11/18 (61.1%) |
| 0.5SHRZ_5/7_ / 5.5HP_1/7_ | ND | 0/16 | 10/18 (55.6%) |
| 0.5MHRZ_5/7_ / 5.5MHP_1/7_ | 5.30 + 0.45 | 0/19 | 2/13 (15.3%) |
| 0.5MHRZ_5/7_ / 5.5HP_1/7_ | ND | 0/18 | 7/14 (50%) |
| 6SHRZ_1/7_ | 5.56 + 0.25 | 1/19 | 7/12 (58.3%) |

Finally, Nuermberger et al. recently conducted a 6-month treatment study in the murine model designed to test the potential of moxifloxacin to improve on the sterilizing activity of first-line TB drugs[19]. The experimental conditions were intended to simulate the treatment of active human TB. Mice were infected via the aerosol route and treatment was started when the bacterial burden approached 10^8^ CFU in the lungs (similar to the population in a human tuberculous cavity). Mice were treated by gavage with the standard regimen (2 months HRZ + 4 months HR) or with test regimens in which moxifloxacin was added or substituted for each of the individual components. The activity of each regimen was determined by monthly lung CFU counts. To assess sterilization (or cure), mice from each group were held an additional 3 months after the end of treatment to determine the rate of relapse (positive lung culture). The results of lung CFU counts are shown in Figure 2.

The most dramatic improvement in sterilizing activity occurred with the substitution of moxifloxacin for isoniazid. Treatment with RMZ resulted in a 2.5-log greater reduction in CFU counts relative to RHZ at 2 months (p <0.001). After 3 months of RMZ/RM, only 2 of 5 mice had positive lung cultures, with only 1 CFU per mouse. In contrast, 6/6 mice given RHZ/RH were still culture-positive at 4 months. These data suggest that the combination RMZ has dramatically greater sterilizing activity than the standard regimen and may be able to shorten the duration of therapy for human TB by 2 months or more. The study did not, however, determine the shortest duration of therapy with this combination that results in acceptably low relapse rates.

**Figure 2.** Lung CFU counts during combination therapy with MXF-containing regimens. Doses (in mg/kg): R 10, H 25, Z 150, M 100.

A second 6-month treatment study designed to determine the shortest duration of therapy with RMZ to successfully prevent relapse is nearing completion. Mice were infected by the aerosol route and treatment was initiated 3 weeks after infection. Mice were assigned to receive either the standard regimen of 2RHZ+4RH or experimental regimens of 2RMZ+3RM, 1RMZ+4RM, or 5RMZ. After 3, 4, and 5 months of treatment, groups of 12 or more mice receiving each regimen were held without treatment for an additional three months and then euthanized to determine the culture-positive relapse rate (Table 4). Four months of therapy with any of the RMZ-based regimens resulted in complete sterilization (cure) of all mice, whereas 4 months of therapy with the standard RHZ-based regimen resulted in a relapse rate of 42%. The relapse rate remained above 6% after even 5 months of the RHZ-based regimen. These preliminary data extend previous observations and further confirm the exceptional sterilizing activity of RMZ-based regimens, suggesting that the use of RMZ-based regimens may permit shortening the duration of therapy by at least 2 months. Moreover, the data suggest that the use of pyrazinamide beyond the first month of therapy did not improve the rate of cure.

**Table 4.** Relapse rates for mice treated with RMZ-based regimens of different durations compared to rates for mice treated with the RHZ-based standard regimen

|  | **Proportion of mice relapsing by regimen and number of months of treatment received** | | |
| --- | --- | --- | --- |
| **Assigned Regimen** | **3 Months** | **4 Months** | **5 Months** |
| 2RHZ + 3 RH | 11/12 | 5/12 | 1/16 |
| 2RMZ + 3RM | 2/12* | 0/12* | 0/13 |
| 1RMZ + 4RM | 4/12** | 0/12* | 0/12 |
| 5RMZ | 4/12** | 0/12* | 0/12 |

*p<0.01 vs. standard regimen, **p<0.05 vs. standard regimen

One important caveat must be stated with the results of the preceding two studies. While the dosages of isoniazid, rifampin, and pyrazinamide were bioequipotent to those given in man, the dosage of moxifloxacin used in these experiments (100 mg/kg) has subsequently been determined to be less than equipotent. In fact, 200 mg/kg is the once daily dosage in the mouse that provides an AUC similar to that in humans after a 400 mg dose (Nuermberger et al, personal communication). Since moxifloxacin has dose-dependent activity over this dose range[17], the results described above were achieved with a moxifloxacin dosage likely to underestimate that drug’s activity in humans.

Taken together, these *in vitro* and animal model studies suggest that moxifloxacin may be a valuable drug for tuberculosis treatment. Sterilizing activity is the ability of a drug to prevent relapse of tuberculosis after treatment completion. At present, there are only two drugs with potent sterilizing activity – rifampin and pyrazinamide. The combination of these two drugs allows tuberculosis treatment to be shortened from 18 months to 6 months with relapse rates of 5% or less[6]. The animal model studies suggest that moxifloxacin may have potent sterilizing activity and the potential to further shorten the duration of therapy.

**Clinical experience with fluoroquinolones for tuberculosis treatment**

Several fluoroquinolones have been used in the treatment of MDR-TB. These studies suggested that fluoroquinolones have activity against *M. tuberculosis*, but the exact role of fluoroquinolones in treatment could not be determined from these non-randomized studies[20, 21]. Fluoroquinolones have early bactericidal activity[22, 23]. This activity is particularly notable because previous studies evaluated members of the class (ciprofloxacin, ofloxacin) having less *in vitro* activity than moxifloxacin. Studies to date indicate that the early bactericidal activity of moxifloxacin is not significantly different than that of isoniazid[24, 25]. The effect of levofloxacin on 2-month culture conversion rates was evaluated in a randomized trial among HIV-infected patients. Culture conversion was somewhat more rapid among patients randomized to receive levofloxacin, but this difference was not evident at 8 weeks of therapy and was not statistically significant[26]. However, culture conversion rates were unusually high in this trial (97% by 2 months), decreasing the ability to detect a difference due to levofloxacin. A similar trend was seen in a second, relatively small trial comparing ofloxacin vs. ethambutol (in combination with isoniazid and rifampin)[27].

The effects of fluoroquinolone-containing regimens on relapse rates have been evaluated in two clinical trials. An early randomized trial in which ciprofloxacin replaced pyrazinamide and ethambutol showed that the ciprofloxacin regimen was less active, primarily among patients with HIV co-infection[28]. However, as outlined above, ciprofloxacin is much less active against *M. tuberculosis* than newer fluoroquinolones, such as moxifloxacin. Therefore, this negative result probably reflects the use of a sub-optimal member of this class, not a demonstration that fluoroquinolones are ineffective in treatment of tuberculosis. Recently, regimens of 4 and 5 months duration including ofloxacin (400-600 mg) isoniazid, rifampin, and pyrazinamide appeared to be quite successful for smear-positive pulmonary tuberculosis, suggesting that newer fluoroquinolones may be potent sterilizing drugs (Table 5)[29]. However, interpretation of this clinical trial is hampered by the failure to include a standard 6-month control regimen and by the analytic techniques used. For example, the calculation of relapse rates eliminated 188 patients (36% of those enrolled) because of nonadherence, unfavorable results at the end of therapy, and other reasons.

**Table 5.** Results of a randomized trial of ofloxacin-containing regimens of less than 6 months duration for treatment of smear-positive pulmonary tuberculosis[29]

| *Regimen (n)* | *2-month culture conversion rate (%)* | *Relapse rate (%)* |
| --- | --- | --- |
| 3OHRZ_7/7_ (83) | 92-98% | 8 |
| 3OHRZ_7/7_ / 1HR_2/7_ (81) |  | 4 |
| 3OHRZ_7/7_ / 2HR_2/7_ (86) |  | 2 |
| 2OHRZ_7/7_ / 2HR_2/7_ (91) |  | 13 |

In summary, the existing clinical data are consistent with the *in vitro* and animal model studies. Early fluoroquinolones, such as ciprofloxacin, have limited activity in tuberculosis treatment, but newer fluoroquinolones may have potent sterilizing activity. However, the activity of newer fluoroquinolones has not yet been fully evaluated in a properly controlled clinical trial.

A notable feature of all of the human clinical studies of fluoroquinolones has been the tolerability and safety of these drugs. Adverse events were comparable in the fluoroquinolone-containing and control arms in the randomized trials (Table 6)[26, 30]. Although adverse events are common among patients being treated for MDR-TB, these are seldom thought due to fluoroquinolones, even when used daily for as long as 18 months[20, 31]. Valerio et al found that the combination of moxifloxacin, isoniazid, and rifampin given for 6 months was well-tolerated[32].

**Table 6.** Percentage of patients who developed adverse events during the first 2 months of therapy [26]

| *Adverse events (AE)* | *HRZE*  *(n = 87)* | *HRZE+ Levo*  *(n = 87)* |
| --- | --- | --- |
| Any reportable AE | 17.2 | 17.2 |
| Any AE probably related to study drug | 5.8 | 4.6 |
| Any drug permanently discontinued | 11.5 | 8.1 |
| Hepatotoxicity | 5.8 | 1.2 |
| Skin rash with fever | 2.3 | 2.3 |
| Hematological toxicity | 2.3 | 3.5 |
| Renal / metabolic toxicity | 1.2 | 3.5 |
| Nausea / vomiting | 0 | 1.2 |

Use of 2-month sputum culture conversion as a surrogate for sterilizing activity

An appropriate surrogate marker of the sterilizing activity of antituberculosis drugs is the rate of conversion of sputum culture to negative among patients with pulmonary tuberculosis. For example, in two randomized trials, the addition of rifampin to regimens based on isoniazid and streptomycin (with or without pyrazinamide) increased 2-month culture conversion rates by 20%. The clinical correlate of this marked increase in culture conversion rate is the ability to decrease the duration of therapy from 18 months to 9 months. Similarly, the addition of pyrazinamide to regimens including isoniazid and rifampin increased 2-month culture conversion rates by an average of 12.7% (Table 7). The clinical correlate of this evidence of pyrazinamide’s sterilizing activity was the ability to decrease therapy from 9 months to 6 months.

**Table 7.** Effect of the addition of pyrazinamide on two-month culture conversion rate in randomized trials

| *Regimen*  *(reference)* | *N* | *2-month sputum culture*  *conversion rate* | *Difference* |
| --- | --- | --- | --- |
| SH [33] | 112 | 49% |  |
| SHZ | 153 | 66% | 17% |
|  |  |  |  |
| SHR [34] | 171 | 70% |  |
| SHRZ | 338 | 82% | 12% |
|  |  |  |  |
| SHR [35] | 159 | 75% |  |
| SHRZ | 156 | 87% | 12% |
|  |  |  |  |
| SHR [36] | 143 | 88% |  |
| SHRZ | 174 | 95% | 7% |
| SHRE | 168 | 81% | 14% |
|  |  |  |  |
| S_3_H_3_R_3_Z_3_ [37] | 151 | 90% |  |
| S_3_H_3_R_3_E_3_ | 166 | 76% | 14% |
|  |  |  |  |
| Average difference |  |  | 12.7% |

S – streptomycin, H – isoniazid, R – rifampin, Z – pyrazinamide, E – ethambutol

In addition to these examples of the correlations between higher 2-month culture conversion and the ability to shorten therapy, there are examples of the converse – lower 2-month culture conversion being associated with higher rates of relapse. The substitution of ethambutol for pyrazinamide decreased 2-month culture conversion rate by 14% and was associated with a higher relapse rate (8% vs. 2%). Similarly, the use of ciprofloxacin in place of pyrazinamide resulted in a decrease in 2-month culture conversion rate and a higher relapse rate[23, 28]. Therefore, 2-month culture sputum conversion rate is an accepted measure to evaluate the potential sterilizing activity of a new anti-tuberculosis drug. Demonstration of an increased 2-month culture conversion rate with moxifloxacin would be a key step in proceeding to phase 3 trials of tuberculosis treatment regimens shorter than 6 months, given less frequently (once-weekly) or both. Any of these changes would represent substantial advances in tuberculosis treatment.

Directly-observed therapy is recommended for patients with pulmonary tuberculosis. Use of directly observed therapy results in higher rates of treatment completion[38], decreased emergence of drug-resistance[39], and decreased overall tuberculosis case rates[40, 41].

**Factors other than therapeutic regimen known to affect 2-month culture-conversion**

##### Cavitation

Cavitation on chest radiograph is associated with a much lower rate of 2-month culture-conversion than is the case for patients with non-cavitary infiltrates (Table 8). This is true whether or not the initial sputum specimens were smear-positive or smear-negative for acid-fast bacilli. Therefore, randomization will be stratified by presence or absence of cavitation on the initial chest radiograph.

# Table 8. The effect of cavitation on 2-month culture conversion in TBTC Study 22 (TBTC, unpublished data)

*2-month culture conversion rate*

|  | *Cavitation* | *No cavitation* |
| --- | --- | --- |
| All HIV-negative patients | 70% (315/450) | 90.4% (356/394) |
| Only patients with initial smear-positivity | 66.6% (241/362) | 84.5% (169/200) |

##### HIV infection

A substantial proportion of patients enrolling in this trial will have HIV infection. Given the tremendous numbers of patients with HIV-related tuberculosis in the world, it is critical that any new treatment regimen be evaluated among HIV-infected persons. Although HIV infection was associated with a higher rate of 2-month culture conversion in TBTC Study 22[42], this difference did not retain significance after adjusting for the presence of cavitation, and other studies have not found a consistent association between HIV infection and 2-month culture-conversion rate (Table 9).

# Table 9. The effect of HIV infection on 2-month sputum culture conversion

*2-month culture conversion rate*

| *Study* | *HIV-positive* | *HIV-negative* |
| --- | --- | --- |
| Study 22 (TBTC, unpublished data) | 90.4% (47/52) | 79.4% (682/865) |
| Zaire [43] | 92.9% (223/240) | 92.1% (164/178) |
| Sterling (time to culture conversion) [44] | 77 days | 72 days |

INH resistance

Mycobacterial culture and drug susceptibility testing can require up to 8 weeks. Therefore, patients having drug-resistant isolates are likely to be enrolled in this trial and treated for most of the intensive phase of therapy prior to the receipt of the drug-susceptibility test results. The most common form of drug resistance in the United States, Canada, and Uganda is INH-resistance, with rates of 5-10% among patients without prior tuberculosis treatment. INH-resistance does not appear to have a substantial impact on sputum culture conversion (Table 10). However, INH-resistance appears to be associated with slightly higher rates of treatment failure and relapse, raising the possibility that absence of difference in 2-month sputum culture conversion rates may not indicate equivalence. In addition, current TB treatment guidelines suggest use of rifampin, pyrazinamide, plus ethambutol for individuals with INH-resistant isolates. Therefore, patients with INH-resistant isolates will be taken off of their assigned study regimen and treated using an individualized regimen at the discretion of the principal investigator. These patients will not be included in the analysis of sputum culture conversion.

**Table 10.** Effect of initial resistance to INH on outcomes of TB treatment [45]

| Outcome | INH-resistance | Drug-susceptible |
| --- | --- | --- |
| 2-month sputum culture conversion | 88% (132 / 150) | 89% (1123 / 1262) |
| Treatment failure | 0.6% (1 / 172) | 0.1% (2 / 3177) |
| Relapse | 9.0% (13 / 149) | 4.0% (123 / 2954) |

# The effect of laboratory techniques on the yield of sputum culture

A variety of culture systems are used to grow *M. tuberculosis*, and TBTC and the Uganda sites do not have a standardized culture system. No single culture technique has 100% sensitivity. However, as shown in Table 11, the use of a broth culture medium increases the yield of sputum culture by about 7%. Furthermore, the yield of using broth and solid media is consistently higher than using any one of these techniques alone. In a study using sputum culture conversion as the primary endpoint, it is important to standardize sputum culture techniques as much as possible. In this trial, all sites will be required to use both a licensed broth culture system and solid media for sputum cultures after enrollment. Reporting of results from both broth and solid media is required for each sputum specimen.

**Table 11.** The effect of culture technique on the yield of sputum mycobacterial cultures

| *Study* | *BACTEC 460* | *MGIT* | *BACTEC 9000* | *MB/BacT* | *Solid media* | *Difference between BACTEC 460 and solid media* |
| --- | --- | --- | --- | --- | --- | --- |
| Stager (n = 143)[46] | 89% |  |  |  | 80% | 9% |
| Wilson (n = 230) [47] | 92% |  |  |  | 86% | 6% |
| Idigoras (n = 315) [48] | 93% |  |  |  | 80% | 13% |
| Hanna (n = 130) [49] | 90% | 77% |  |  | 79% | 11% |
| Manterola (n = 100) [50] | 90% |  |  | 98% | 94% | -4% |
| Somoskovi (n = 55) [51] | 93% | 96% |  |  | 82% | 7% |
| Brunello (n = 115) [52] | 100% |  |  | 99% | 98% | 2% |
| Pfyffer (n = 247) [53] | 92% |  | 84% |  | 85% | 7% |
| Average | 92.4% | 86.5% | 84% | 98.5% | 85.8% | 6.6% |

Approximately 3-5% of sputum cultures for mycobacteria have bacterial or fungal overgrowth (despite decontamination procedures), making it impossible to determine whether there may have been mycobacteria present as well. To maximize the proportion of patients who can be analyzed for the primary endpoint, 2-month culture conversion, 2 sputum specimens will be collected at the completion of the four-drug phase of therapy.

# Risk factors for treatment failure and relapse

Large clinical trials, including TBTC Study 22, have identified consistent risk factors for poor outcomes from tuberculosis therapy (treatment failure or relapse). The most potent risk factors include the presence of cavitation on chest radiograph and 2-month sputum culture-positivity[42, 54, 55]. Patients with both of these factors had a particularly high rate of treatment failure or relapse (22% in TBTC Study 22) [42]. The consistency of the effect of 2-month culture conversion on risk of relapse has led to the recommendation that therapy be changed for such patients (ATS/IDSA/CDC guidelines for treatment of tuberculosis). The optimal management of patients at high risk for treatment failure or relapse will require additional study. The most promising management strategy is the extension of therapy. In a randomized trial of the treatment of silicotuberculosis, the extension of therapy by an additional 2 months (from 6 to 8 months) decreased the relapse rate from 20% to 2%[56]. In this trial patients with cavitation and 2-month culture positivity will be treated as recommended by the ATS/IDSA/CDC guidelines – an extension of therapy by 3 months.

- 1. Study Drugs

# Moxifloxacin

Moxifloxacin is a fluoroquinolone antibacterial, which is distinguished by a methoxy group at the C-8 position, and an S,S-configured diazabicyclononyl ring moiety at the C-7 position. The mechanism of action against *M. tuberculosis* is thought to be by inhibition of the DNA gyrase enzyme involved in DNA replication.


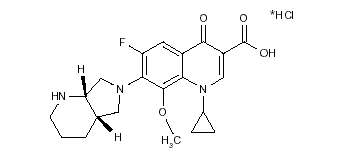


Moxifloxacin structure [Diagram obtained from package insert] [57]

##### Pharmacokinetics


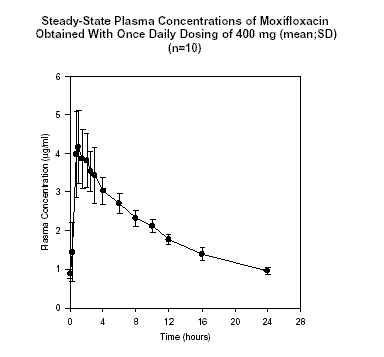
Moxifloxacin is well absorbed, with a bioavailability of approximately 90% [58]. Pharmacokinetics are linear in the range of 50-800 mg single dose and up to 600 mg once daily dosing over 10 days[59]. Steady state is reached within 3 days[60]. The mean (+SD) maximum concentration (C_max_ ) and AUC values at steady state with a 400 mg once-daily dosage regimen are 4.5+0.53 mcg/ml and 48+2.7 µg*h/ml, respectively[61]. T_max_ is approximately 1-3 hours. The mean (+SD) plasma half-life is 12.1 + 3.1 hours. Trough plasma concentration at steady state (400 mg once daily) is 0.95 + 0.10 mcg/L. Co-administration with food may slightly prolong T_max_, and may reduce the C_max_ by 16%; these effects are not believed to be clinically significant, and thus moxifloxacin can be administered with or without food. Administration with yogurt[62] or a high fat meal[63] did not significantly affect the AUC. However, concomitant administration with certain medications may substantially diminish the absorption of all fluoroquinolones (see below). Moxifloxacin is 50% bound to plasma proteins. It is widely distributed, with some tissue concentrations reported in excess of plasma levels[64].

Moxifloxacin is metabolized by glucuronide and sulfate conjugation. The cytochrome p450 enzyme system is not involved in the metabolism of moxifloxacin, nor is it affected by the drug. Specifically, moxifloxacin does not affect CYP3A4, CYP2D6, CYP2C9, CYP2C19, or CYP1A2. The sulfate conjugate (M1) accounts for 38% of the oral dose, and is excreted in feces; about 14% of an oral dose is converted to the glucuronide conjugate (M2), and is excreted in urine[65]. Peak plasma levels of M1 and M2 are <10% and about 40% those of the parent drug, respectively. Overall, about 45% of an oral dose is excreted as parent drug, and about 51% as known metabolites.

# Table 12. Moxifloxacin pharmacokinetics in special populations

| Population | Dose/Regimen | Findings |
| --- | --- | --- |
| Healthy elderly (16) [66] | Single 200-mg dose | AUC and Cmax similar in young and elderly males |
| Elderly (16) and young (18) males and females [66] | Elderly: single 200-mg dose  Young: single 400-mg dose | No gender differences when weight differences considered |
| Japanese males | Steady state | Similar to Caucasians |
| Renal insufficiency (24) [67] | Single dose | In patients with moderate and severe impairment, Cmax was reduced 21-22%; AUC increased 13%; AUC-M1 increased 1.7-fold; AUC-M2 increased 2.8-fold |
| Hepatic insufficiency (16: 6 Child Pugh Class A, & 10 Child Pugh Class B) | Single 400-mg dose | In class B patients, AUC of parent drug was unchanged; AUC-M1 increased 5.7-fold; AUC-M2 increased 1.5-fold; no data on Class C |
| Continuous ambulatory peritoneal dialysis (CAPD) or hemodialysis |  | Not studied |
| Pediatric |  | Not studied |

### Drug-drug interactions

Interaction studies have been performed with moxifloxacin and theophylline[68], warfarin, digoxin, probenecid[69], ranitidine, glyburide[70], iron[71], calcium supplements[72], sucralfate[73], and antacids[74]. Only a few clinically significant interactions have been described. All fluoroquinolones can bind to multivalent cations (esp. Fe^+3^, Al^+3^, and Mg^+2^, but not Ca^+2^], with resultant substantial (40-60%) decreases in absorption when taken concurrently. Moxifloxacin should not be administered at the same time as antacids containing magnesium and/or aluminum, sucralfate, antidiarrheals that contain kaolin, or iron and/or zinc containing medications or supplements.

Rifampin induces the activity of the phase II enzymes glucuronosyltransferase and sulphotransferase [75]. Interaction studies between rifampin and moxifloxacin have not been performed. However, because glucuronidation is a minor pathway in moxifloxacin metabolism (accounting for approximately 20% of total hepatic metabolism), a significant interaction between rifampin and moxifloxacin is unlikely.

Tolerability

As a class, fluoroquinolones are considered to be safe and relatively free of serious adverse effects[76]. The safety and efficacy of moxifloxacin for the treatment of community acquired bacterial infections has been extensively studied, and the drug is approved in the U.S. as a 10-day course of treatment for upper and lower respiratory tract infections.

In the usual daily doses of 400 mg/day, moxifloxacin is well tolerated. Expected minor adverse events have occurred with a frequency similar to that for other fluoroquinolones in similar populations. Gastrointestinal adverse effects are the most common, and they include nausea (7%), diarrhea (6%), dizziness (3%), abdominal pain (2%), vomiting (2%), dyspepsia (1%), and taste perversion (1%). There are no current reports of hepatitis, although abnormal liver function tests have been noted in 1% of patients.

Of note, fluoroquinolones are generally used short-term for acute conditions. However, fluoroquinolones have been found to be safe and effective in long term use for chronic infections such as osteomyelitis, prostatitis, or chronic urinary tract infection, or skin and skin structure infections[76, 77]. In addition, Valerio et al. found that the combination of moxifloxacin, isoniazid, and rifampin given for 6 months was well tolerated[32].

## Cardiotoxicity

The QT interval is the electrocardiographic measurement that describes the period between onset of ventricular depolarization and the end of the repolarization process. Prolongation of this interval is thought to be associated with an increased risk of ventricular tachy-arrhythmias. Moxifloxacin causes a mild prolongation of the corrected QT (QT_c_) interval[78]. The mean effect on QT_c_ interval in 787 patients in Phase 3 clinical trials was 6 + 26 milliseconds, but no morbidity or mortality was attributable to QT_c_ prolongation. In a review of over 6,000 patients treated with moxifloxacin, there were no reports of changes in the frequency of QT_c_ prolongation[79]. In over 10 million patients treated with moxifloxacin there has been no evidence for an increased incidence of ventricular arrhythmia when compared to the overall population. No ventricular arrhythmias attributable to QT_c_ prolongation have been reported to date. Finally, the incidence of sudden death and syncope, both markers for sudden cardiac events, is not increased when compared to the incidence on the general population. For these reasons, electrocardiographic monitoring is no longer recommended for clinical trials involving moxifloxacin.

In this study, moxifloxacin will not be used among patients with a known history of prolongation of the QT interval, patients with uncorrected hypokalemia and patients receiving class Ia (e.g. quinidine, procainamide) or class III (e.g. amiodarone, sotalol) antiarrythmic agents, due to the lack of clinical experience with the drug in these patient populations (see Exclusion criteria). Pharmacokinetic studies between moxifloxacin and other drugs that prolong the QT interval such as cisapride, erythromycin, antipsychotics and tricyclic antidepressants have not been performed. An additive effect of moxifloxacin and these drugs cannot be excluded, therefore moxifloxacin should be used with caution when given concurrently with these drugs.

Phototoxicity/Photosensitivity

Photosensitivity is defined as a non-immunological, light-activated irritation that occurs following exposure to a photoactive chemical. This is an infrequent adverse event of most fluoroquinolones. Moxifloxacin, has been found to have no negative phototoxic effects and in some patients was shown to be photoprotective at specific wavelengths.

## Tendinopathy/Tendinitis

The estimated incidence of fluoroquinolone-induced tendinopathy is 15 to 20 per 100,000[80]. Fluoroquinolone-induced tendinopathy is diagnosed by a sudden onset of swelling and tenderness concurrent with or shortly after fluoroquinolone therapy, which is accompanied by tendon rupture in about 33% of all cases[81]. The main site affected is the Achilles tendon, though tendinitis has been reported to involve the shoulder, knee, hand, and plantar aponeuroeses[82]. Achilles tendon ruptures have been noted even months after discontinuation[83]. Uncomplicated tendinitis is generally seen when the duration of therapy is less than 5 days, and tendon rupture more frequently when therapy lasts longer than 3 weeks[80, 81]. Concomitant use of corticosteroids is considered to be a risk factor for developing tendinopathy while taking fluoroquinolones[84]. Treatment involves discontinuation of the fluoroquinolone and resting the tendons. Physical therapy may be needed early in treatment and may be prolonged[81].

In over 10 million patients treated with moxifloxacin only 3 patients with tendon rupture have been reported. All had received concomitant corticosteroid treatment.

## Retinal toxicity

Ocular toxicity was not observed in 6-month repeat dose studies in rats and monkeys. In beagles, electroretinographic changes were observed in a 2-week study at doses of 60 and 90 mg/kg. Histopathological changes were observed in the retina from 1 of 4 dogs at 90 mg/kg, a dose associated with mortality in this study[85]. In summary, ocular toxicity in animals has only been observed in one species (dogs) at a dose that was associated with severe systemic toxicity.

Ocular toxicity has not been reported from clinical trials or post-marketing surveys involving moxifloxacin (or any other fluoroquinolone antibiotics). Monitoring for retinal toxicity will be performed among all patients in this clinical trial because of the inclusion of ethambutol, a drug with known retinal toxicity (see below). The visual testing done to detect ethambutol retinal toxicity should be adequate to screen for possible retinal toxicity from moxifloxacin.

# Ethambutol

Ethambutol is an ethylene-diimino derivative of butanol that interferes with cell wall synthesis in mycobacteria; other bacteria are uniformly resistant to ethambutol. In the treatment of human tuberculosis ethambutol is effective in preventing the emergence of drug resistant strains, although it has no sterilizing activity at clinically-tolerated doses[27].

# Pharmacokinetics

Ethambutol is well absorbed from the gastrointestinal tract, reaching peak serum concentrations of 3-5 mcg/ml in normal volunteers 2-4 hours after a dose. Food slows absorption and decreases the peak serum concentration by 10-20%, but has no effect on the total systemic exposure (AUC). Antacids decrease both the peak serum concentration and AUC, and so should not be administered at the same time. Ethambutol is primarily eliminated by the kidneys as unchanged drug; the serum half-life averages 4 hours[28]. Patients with renal insufficiency are prone to accumulation of the drug and the resultant toxicity.

### Drug-drug interactions

There are no known drug–drug interactions involving ethambutol

### Toxicity

Ethambutol is usually well-tolerated with low rates of skin rash, nausea, vomiting, or diarrhea. Fever, allergic reactions, abdominal pain, mental status changes, peripheral neuropathy, and increased liver function tests have rarely been associated with ethambutol. Adverse events occur in less than 2% of patients receiving ethambutol at the 15 mg/kg dose and include decreased visual acuity (0.8%), rash (0.5%) and asymptomatic hyperuricemia[86]. The most common serious side effect of ethambutol is retinal toxicity, often first perceived as a decrease in color perception. Patients receiving ethambutol should be instructed about symptoms of ocular toxicity. If stopped promptly, permanent visual loss is rare among patients with ethambutol-related retinal toxicity. Rates of retinal toxicity are very low when the drug is given for relatively short periods of time, as is the case in this study.

# Isoniazid

Isoniazid is the hydrazide of isonicotinic acid and is one of the primary drugs for tuberculosis treatment. The activity of isoniazid is limited to the mycobacteria of the *M. tuberculosis* complex; it is bactericidal for rapidly dividing organisms and bacteriostatic for “resting” bacilli . The probable mechanism of action is the inhibition of the biosynthesis of mycolic acids, a component of the mycobacterial cell wall. Its impact on the cell wall structures has therefore similarities with that of penicillin, and might have similar therapeutic interferences with other bactericidal drugs.

### Pharmacokinetics

Isoniazid is generally well absorbed; food and antacids decrease the rate, but not the extent of absorption. The peak blood levels of isoniazid, 3 to 5 mcg/ml, are obtained 30 minutes to 2 hours after ingestion of routine doses[87]. It diffuses into all body fluids and cells and penetrates into the caseous material of a tuberculoma or pulmonary cavity. In the liver, it is acetylated to inactive metabolites, and 75% to 95% of the dose is excreted as inactive metabolites in the urine within 24 hours.

Isoniazid clearance rates depend on two metabolic phenotypes, slow and fast acetylation, which are associated with race, but not gender[88]. The prevalence of slow acetylation in the United States is approximately 50%. The isoniazid AUC among persons who have fast acetylation is 30% to 50% of that among persons who have slow acetylation. Because isoniazid is well tolerated over a wide range of therapeutic doses, a single dose per body mass is recommended. Persons who have rapid acetylation achieve effective concentrations, while persons who have slow acetylation do not experience increased toxicity. Half-life (t_1/2_) may vary from 1 hour in fast acetylators (t_1/2_ < 90min) to 3 hours in slow acetylators (t_1/2_ >90min).

### Drug-drug interactions

Isoniazid decreases the clearance of some medications that are metabolized in the liver (particularly carbamazepine, phenytoin, diazepam)[89]. However in the context of multidrug therapy including rifampin, these potential drug-drug interaction are of little significance because the effect of isoniazid is counteracted by the more potent opposing effect of rifampin [90] (see below).

### Toxicity

The total incidence of all adverse effects from isoniazid is approximately 5%, many of which do not require discontinuation of the drug. Peripheral neurotoxicity is dose dependent and it is uncommon (<0.2%) at conventional doses. The risk of peripheral neuritis increases for persons who are malnourished or predisposed to neuritis by other illnesses. Concomitant administration of pyridoxine (vitamin B_6_) is recommended for these persons, and will be given to all patients in this trial. Other nervous system reactions are rare at normal doses, and they include convulsions, encephalopathy, optic neuritis, memory impairment, and psychosis. Gastrointestinal adverse effects include nausea, vomiting, and epigastric distress.

Asymptomatic elevation of aminotransferases is common and occurs in 10-20% of persons receiving isoniazid. However, idiosyncratic severe hepatic reactions are uncommon but are more likely in older persons (up to 2.3% hepatitis incidence in persons more than 50 years old), and may be life threatening. Daily consumption of alcohol increases the risk of isoniazid-associated hepatotoxicity by approximately four-fold. The risk of isoniazid-induced hepatotoxicity may also be increased in the postpartum period. The prodromal symptoms of hepatotoxicity are anorexia, nausea, vomiting, fatigue, malaise, and weakness; persons who take isoniazid and have these symptoms should stop therapy and be evaluated immediately.

# Rifampin

Rifampin is a semi-synthetic rifamycin B derivative that is highly active against mycobacteria, most gram-positive bacteria, and some gram-negative bacteria. It is bactericidal for both intracellular and extracellular microorganisms. By inhibiting prokaryotic DNA-dependent RNA polymerase, it suppresses the early elongation of the nucleotide chain in RNA synthesis.

### Pharmacokinetics

Rifampin is normally absorbed completely when taken orally, but food delays absorption. After 1.5 to 2 hours, a 600 mg dose yields a peak blood level of 8-20 mcg/ml. The half-life of rifampin varies from 2 to 5 hours, and it is shortened by approximately 20-40% after the first week of daily treatment because of the induction of hepatic microsomal enzymes. The half-life is unaffected by renal impairment but is increased by liver disease or biliary obstruction. Rifampin is deacetylated to an enterohepatically-recirculated active metabolite, and 50% to 60% is excreted in the feces. Up to 30% of a dose is excreted in the urine. Approximately 85% of circulating rifampin is bound to plasma proteins, and is widely distributed throughout the body.

### Drug-drug interactions

Rifampin is a potent inducer of a number of hepatic enzymes involved in the metabolism of drugs and some hormones[91]. This enzyme induction causes more rapid elimination (and potential loss of efficacy) of many drugs. For some medications, this loss in pharmacological activity effect is dramatic, and rifampin cannot be used if one of these medications is thought to be essential. Medications for which concomitant rifampin is contraindicated include: HIV-1 protease inhibitors (other than ritonavir), delavirdine, cyclosporine, tacrolimus, itraconazole, and ketoconazole. For many other medications, the dose can be increased to compensate for the effect of rifampin (see section on Concomitant Medications).

### Toxicity

In the usual daily doses of 10 mg/kg (maximum 600 mg), rifampin is well tolerated. It often causes harmless but disconcerting red-orange discoloration of tears, sweat, saliva, feces, and urine. Less than 4% of TB patients experience significant adverse reactions to rifampin. Gastrointestinal adverse effects are the most common, and they include epigastric distress, anorexia, nausea, vomiting, cramps, and diarrhea. Hepatitis rarely occurs in persons who have normal baseline hepatic function. The incidence of hepatitis may be increased for older persons and those who have chronic liver disease or alcoholism, but remains substantially lower than that for pyrazinamide or isoniazid. Rifampin can cause a flu-like syndrome of fever, chills, and myalgia, although this is uncommon using the 600 mg dose given daily or thrice-weekly. In a very small proportion of patients the flu-like syndrome is associated with interstitial nephritis, acute tubular necrosis, thrombocytopenia, hemolytic anemia, and shock. There may be changes in menstruation.

# Pyrazinamide

Pyrazinamide is an analog of nicotinamide and has unique activity against *M. tuberculosis*. In liquid culture media, pyrazinamide has little activity except when the culture is made acidic. However, in animal models and in the treatment of human tuberculosis pyrazinamide has unique sterilizing activity, allowing the duration of treatment to be decreased from 9 months to 6 months (assuming rifampin is used throughout). The mechanism of action of pyrazinamide remains unknown.

Pharmacokinetics

Pyrazinamide is well-absorbed from the gastrointestinal tract and widely distributed into all tissues, including the cerebrospinal fluid[92]. Usual doses are 15-30 mg/kg/d, up to 2 gm/d. Peak serum concentrations of about 45 mcg/ml are achieved approximately 2-3 hours after a dose. Food and antacids do not significantly affect the absorption of pyrazinamide. The half-life of pyrazinamide is approximately 9-10 hours[93], and is prolonged in the presence of hepatic insufficiency[94]. Pyrazinamide is metabolized to pyrazinoic acid by the hepatic microsomal enzyme pyrazinamide deamidase. Approximately 40% of a dose is recovered in the urine as pyrazinoic acid and an additional 4% is excreted in the urine as the unchanged parent drug[95]. The remaining drug is thought to be excreted in the bile.

### Drug-drug interactions

There are no known clinically significant drug-drug interactions involving pyrazinamide.

### Toxicity

The most frequent side effects are skin rash, gastrointestinal intolerance, hepatotoxicity (1.3%), arthralgias (1-7%), hyperuricemia due to blockade of urate excretion (up to 66%), and rarely acute gouty arthritis[86, 96]. These side effects are seldom dose-limiting. Asymptomatic elevations in serum uric acid are frequent, usually occur during the first or second month of treatment, and are self-limited and require no specific treatment[97]. Minor arthralgias also may occur during pyrazinamide treatment and can usually be treated with salicylates or non‑steroidal inflammatory agents such as indomethacin while continuing the drug. The most common serious side effect of pyrazinamide is hepatotoxicity. In two randomized clinical trials the addition of pyrazinamide to isoniazid and rifampin did not increase the rates of hepatotoxicity above that seen with the latter two drugs alone[97, 98]. However, three recent retrospective cohort studies suggest that the incidence of pyrazinamide-induced hepatitis during active TB treatment is higher than that for other first-line TB drugs, and higher than previously recognized[99-101]. Results from these latter studies underscore the importance of identification of new, less toxic yet effective, TB treatment regimens.

**Rifampin/Pyrazinamide toxicity**

In initial randomized clinical trials of a 2-month regimen of rifampin plus pyrazinamide (RZ) treatment for latent tuberculosis in HIV-infected persons, the incidence of severe liver injury was low and similar to that of daily isoniazid[8, 102, 103]. However, subsequent reports described severe liver injury in patients treated for latent tuberculosis infection with RZ, and on the basis of these reports, CDC collected data from cohorts of patients in the United States who received RZ for the treatment of LTBI during the period of January 2000 to June 2002. Of the 7,737 patients started on RZ, 77% received daily doses and 23% twice-weekly doses. A total of 204 patients (2.6 %) discontinued treatment with RZ because of AST concentrations higher than five times the upper limit of normal. An additional 146 patients (1.9%) discontinued using RZ because of symptoms of hepatitis. Of the 30 patients (0.4%) with severe liver injury, 23 recovered and 7 died (0.09%). As a consequence, ATS and CDC recommends against routine use of RZ for treatment of latent TB infection. Several additional latent TB treatment studies confirm a relatively high frequency of hepatitis among RZ recipients – combined data from five recent studies including 1,311 patients showed a rate of Grade 3 or 4 liver injury of 5.8%, which is higher than that reported previously with isoniazid therapy[104-108]. In a randomized trial of two short-course regimens to prevent TB in high risk contacts of active TB cases in Rio de Janeiro, Brazil, Grade 3 or 4 liver injury developed in 17 of 163 (10%) participants receiving twice weekly RZ and <1% of participants receiving isoniazid plus rifapentine (RE Chaisson, unpublished data). The mechanism of RZ-associated hepatotoxicity remains unknown. Similarly, it is unclear why rates of hepatotoxicity with RZ are higher than those observed among patients with active TB treated with isoniazid in addition to rifampin and pyrazinamide for 2 months. Possible explanations include impaired inflammatory response (resulting in impaired liver inflammation) in patients with active versus latent TB, or a mitigating effect of isoniazid on RZ-associated hepatotoxicity.

# METHODOLOGY

- 1. Study Design – 4-drug phase

This is a prospective randomized trial comparing two regimens for treatment of smear-positive pulmonary TB. Patients will be randomized to receive one of the two regimens listed below. All doses of study medications will be given as directly observed therapy.

**Table 13.** Intensive Phase Study Regimens

| **Study Regimen** | **Intensive phase (first 8 weeks of anti-TB therapy)** | |
| --- | --- | --- |
| Control regimen | HRZE daily* (+ moxifloxacin placebo)† |  |
| Moxifloxacin regimen | MRZE daily* (+ isoniazid placebo) † |  |

H – Isoniazid, R – rifampin, Z – pyrazinamide, E – ethambutol, M - moxifloxacin

* Daily therapy will be 5 days per week. However, at the discretion of the investigator, patients in either study arm may be given therapy 7 days per week during the first 2 weeks of the study (only 5 doses will be counted toward completion of the four-drug intensive phase of therapy).

† Vitamin B6 (50 mg) will be given with each dose of therapy

After completion of intensive phase therapy, patients in all study arms will then be treated for 18 weeks (30 weeks for those with cavitation on initial chest radiograph plus a positive sputum culture for *M. tuberculosis* at 2 months of therapy) with an ATS/IDSA/CDC-recommended continuation phase regimen.

Doses of study medications for the intensive phase are given below in Table 14. The duration of the intensive phase of therapy will be determined by the number of doses ingested, not by calendar time. Patients will complete the intensive phase when they have had 40 (5 days per week) directly-observed doses or 44 (7 days per week during the first two weeks then 5 days per week) directly-observed doses. To be eligible for inclusion in the analysis of culture-conversion, this therapy must have been completed within no less than 54 days and no more than 70 days.

# Table 14. Doses of study medications during the intensive phase

| **Drug** | **Dose for daily therapy** |
| --- | --- |
| Moxifloxacin | 400 mg |
| Rifampin |  |
| < 45 kg | 450 mg |
| > 45 kg | 600 mg |
| Isoniazid | 300 mg |
| Pyrazinamide |  |
| < 40 kg | 25 mg/kg rounded to nearest 500 mg* |
| 40-55 kg | 1000 mg |
| > 55 kg - 75 kg | 1500 mg |
| > 75 kg - 90 kg | 2000 mg |
| > 90 kg | 2000 mg |
| Ethambutol |  |
| < 40 kg | 15 mg/kg rounded to nearest 100 mg |
| 40-55 kg | 800 mg |
| > 55 kg - 75 kg | 1200 mg |
| > 75 kg - 90 kg | 1600 mg |
| > 90 kg | 1600 mg |

* for pyrazinamide dosing in patients < 40 kg, 1000 mg typically used instead of 500 mg

Ethambutol may be discontinued if the patient’s *M. tuberculosis* isolate is susceptible to isoniazid, rifampin, pyrazinamide, and fluoroquinolones, as demonstrated by laboratory studies.

- 1. Continuation Phase Therapy

After completing the intensive phase of therapy, patients in all study arms will then be treated with an ATS/IDSA/CDC-recommended continuation-phase regimen. Recommended continuation-phase regimens that may be used include

- isoniazid + rifampin given daily [5 days/wk], thrice-weekly, or twice-weekly
- isoniazid + rifabutin for patients on medications having unacceptable drug-drug interactions with rifampin
- isoniazid + rifapentine once-weekly for HIV-seronegative patients with non-cavitary pulmonary disease plus a negative sputum smear at 2 months
- rifampin + pyrazinamide + ethambutol for patients who are intolerant of isoniazid (note: patients with known isoniazid intolerance cannot be enrolled in the protocol, but patients may develop isoniazid intolerance during study therapy).

The total duration of therapy will be 26 weeks (6 months) except that patients who have cavitation plus a positive sputum culture at the end of the intensive phase of therapy will receive a total of 38 weeks (9 months) of therapy.

- 1. Primary Objective
- To compare the culture-conversion rate at the end of the intensive phase of therapy of the moxifloxacin regimen vs. that of the isoniazid regimen
  1. Secondary Objectives

### To compare the safety and tolerability of the moxifloxacin regimen to that of the isoniazid regimen

### To determine the time to culture-conversion of the moxifloxacin regimen and the isoniazid regimen using data from 2-, 4-, 6-, and 8-week cultures

- To compare the proportion of patients with any Grade 3 or 4 adverse reactions
- To compare adverse events and 2-month culture conversion rates among HIV-infected patients vs. HIV-uninfected patients
- To compare the rates of treatment failure of the moxifloxacin regimen and the isoniazid regimen
- To determine whether there is delayed toxicity attributable to moxifloxacin (toxicity that becomes evident after the 8 weeks of moxifloxacin therapy)
  1. Study Endpoints

**Sputum culture conversion** – sputum culture obtained at the end of the intensive phase of therapy has no growth of *M. tuberculosis*. The end of the intensive phase of therapy will be defined by completion of the required number of directly-observed doses (as defined in Section 3.1), rather than by a specific number of weeks of therapy. In order to assure that each subject will be evaluable for the primary endpoint, two sputum cultures will be obtained at the end of the intensive phase of therapy. These two cultures must have been obtained (a) 0-7 days after the completion of the intensive phase of therapy (preferably, one on the day of completion and one within 7 days of completion), and (b) prior to receiving more than one dose of continuation phase therapy. Sputum culture conversion will be based on the results of both sputum cultures performed at the end of the intensive phase of therapy.  **Both sputum cultures, if evaluable, should be negative for *M. tuberculosis* in order to conclude that sputum culture conversion was achieved.** Sputum cultures that are overgrown by bacteria and/or yeast will be considered unevaluable. Subjects who are unable to produce a specimen for sputum culture despite an attempt with sputum induction will be considered to have had a negative culture on that date.

**Safety and tolerability endpoints** – the primary endpoint for the analysis of safety and tolerability will be the proportion of patients who discontinue the assigned study regimen for any reason. Other aspects of safety and tolerability that will be assessed as secondary endpoints include mortality, the occurrence of Grade 3 and 4 toxicities and the rate and types of toxicity thought related to study drug by the investigator.

**Treatment failure** – a positive sputum culture after completion of 4 months of TB treatment. All treatment failure isolates will be compared to the initial isolate using DNA fingerprinting. A single positive culture having a different DNA fingerprint than the initial isolate will be considered a false-positive culture (due to cross-contamination) and will not be counted as a case of treatment failure.

- 1. Randomization

Eligible patients will be randomized in a 1:1 ratio to the arms. Randomization will be stratified by geographic continent and by the presence of cavitation on the chest radiograph used for screening purposes. Cavitation is defined as a gas-containing lucent space at least 1 cm in diameter within the lung parenchyma surrounded by an infiltrate or fibrotic wall greater than 1 mm thick seen on a standard chest radiograph (cavitation seen only on chest CT, if done, does not satisfy this definition).  Cavities must be distinguished from pulmonary cysts, which are usually thin walled, well-marginated lesions.

- 1. Sample Size Calculation

The culture conversion rate at 8 weeks among patients with initial sputum smear-positivity treated with HRZE who enrolled into TBTC Study 22 was 73.6% (419/574). We hypothesize that the effects of using a fluoroquinolone in place of isoniazid will improve the potency of the intensive phase of therapy by approximately that of the addition of pyrazinamide (mean of 12.7%, see Table 7). To detect an increase in culture conversion from 75% to 88% with a two-sided test at the 0.05 level with 80% power requires 154 patients per group. We will increase this by 25% to compensate for missing cultures, drug resistance, etc. Therefore there will be 205 patients in each of the two arms, 410 patients in the entire study. If, near the culmination of the intended enrollment of 410 patients to achieve 308 evaluable patients, it is determined that patient dropout, missing cultures, etc has exceeded the 25% estimate, then a proportionate number of additional patients will be recruited to retain statistical power.

- 1. Patient Selection

Study entry is open to male and female patients and patients of all ethnic backgrounds.  Historically, the majority of patients with tuberculosis at clinical sites are males.  The gender, ethnic and socioeconomic background of the subjects recruited for this study is expected to mirror that of the population the hospitals and clinics served, and that of the population most affected by tuberculosis. Pregnant or breast-feeding women and children will not be enrolled because of uncertainties about the safety of moxifloxacin in those groups of patients.  Subjects will be enrolled if they meet all of the following inclusion criteria and none of the exclusion criteria:

- - 1. Inclusion Criteria

1. Suspected pulmonary tuberculosis with acid-fast bacilli in a stained smear of expectorated or induced sputum. Patients whose sputum cultures do not grow *M. tuberculosis* and those having an *M. tuberculosis* isolate resistant to (one or more) isoniazid, rifampin, pyrazinamide, or fluoroquinolones will be discontinued from the study, but followed for 14 days to detect late toxicities from study therapy. Patients having extra-pulmonary manifestations of tuberculosis, in addition to smear-positive pulmonary disease, are eligible for enrollment. Sputum must be expectorated or induced; smear results from respiratory secretions obtained by bronchoalveolar lavage or bronchial wash may not be used for assessment of study eligibility.
2. Willingness to have HIV testing performed, if HIV serostatus is not known or if the last documented negative HIV test was more than 6 months prior to enrollment. HIV testing does not need to be repeated if there is written documentation of a positive test (positive ELISA and Western Blot or a plasma HIV-RNA level greater than 5000 copies/ml) at any time in the past
3. 7 (seven) or fewer days of multidrug therapy for tuberculosis disease in the 6 months preceding enrollment
4. 7 (seven) or fewer days of fluoroquinolone therapy in the 3 months preceding enrollment
5. Age > 18 years
6. Karnofsky score of at least 60 (requires occasional assistance but is able to care for most of his/her needs; see Appendix B)
7. Signed informed consent
8. Women with child-bearing potential must agree to practice an adequate (barrier) method of birth control or to abstain from heterosexual intercourse during study therapy.
9. Laboratory parameters done at, or ≤ 14 days prior to, screening:

- Serum amino aspartate transferase (AST) activity ≤ 3 times the upper limit of normal
- Serum total bilirubin level ≤ 2.5 times the upper limit of normal
- Serum creatinine level ≤ 2 times the upper limit of normal
- Complete blood count with hemoglobin level of at least 7.0 g/dL
- Complete blood count with platelet count of at least 50,000/mm^3^
- Serum potassium ≥ 3.5 meq/L
- Negative pregnancy test (women of childbearing potential)
  - 1. Exclusion Criteria

1. Breast-feeding
2. Known intolerance to any of the study drugs
3. Known allergy to any fluoroquinolone antibiotic
4. Concomitant disorders or conditions for which moxifloxacin (MXF), isoniazid (INH), rifampin (RIF), pyrazinamide (PZA), or ethambutol (EMB) are contraindicated. These include severe hepatic damage, acute liver disease of any cause, and acute uncontrolled gouty arthritis.
5. Current or planned therapy during the intensive phase of therapy using drugs having unacceptable interactions with rifampin (rifabutin can be substituted for rifampin during the continuation phase of therapy)
6. Current or planned antiretroviral therapy during the intensive phase of therapy
7. History of prolonged QT syndrome or current or planned therapy with quinidine, procainamide, amiodarone, sotalol, disopyramide, ziprasidone, or terfenadine during the intensive phase of therapy.
8. Pulmonary silicosis
9. Central nervous system TB
10. **STUDY PLAN**
    1. Study Procedures
       1. Screening Visit

After obtaining written informed consent, a sputum sample (expectorated or induced) will be obtained for smear and culture at the laboratory that will be used for the rest of the study (unless one was done in that laboratory within the previous 3 days or less). Patients will have heart rate, blood pressure, respiratory rate, temperature, height and weight measured, and blood will be drawn for a CBC and serum creatinine, AST, total bilirubin, and potassium (and pregnancy test, if patient is a woman of child-bearing potential), unless results from these tests within the previous 14 days or less are available. An HIV test, using any testing method approved by the U.S. Food and Drug Administration (or, for non U.S. study sites, approved by local regulatory agencies), should be obtained at this visit or as soon as possible afterwards. An HIV test does not need to be performed if the patient has HIV infection (documented by a positive ELISA and Western Blot or an HIV-RNA level greater than 5000 copies/mL) or has written documentation of a negative HIV test within the past 6 months or less. For patients with documented HIV infection at screening, a CD4 cell count obtained any time within 6 months prior to initiation of study therapy is also required. A chest radiograph will also be taken, unless a chest radiograph done within the previous 14 days or less is available for review. The chest radiograph must have been read prior to randomization. Tests for visual acuity and color vision testing, per standard site practice, will be performed. Patients will be asked questions related to demographics and level of education. They will also be asked about their current and past medical history, including past or present illnesses, any prior treatment for TB or latent tuberculosis infection, medications they are taking, alcohol, illicit drug use, and TB risk factors. They will be advised that they can decline to answer any question they consider too personal.

- - 1. Randomization

When the results of the blood tests and chest radiograph are available, and if the patient meets the enrollment criteria, the site must contact the TBTC Data and Coordinating Center at CDC, where staff will randomize each new patient by computer. Note, the result of HIV testing is not required for randomization; the requirement is that the patient has agreed to have HIV testing performed. Patients should start on assigned study therapy as soon as possible after randomization. All doses of study therapy will be given as directly observed therapy (DOT) by a health care worker or treatment supervisor who is aware of the study protocol. DOT may be given at the TB clinic or other health care facility, or, with the subject’s permission, at the subject’s residence, workplace, or other mutually agreed upon location convenient for the subject.

A log will be maintained of persons who are eligible but do not enroll.

- - 1. Study visits – intensive phase of therapy

Patients will have study visits 2, 4, 6, and 8 weeks after enrollment. At each of these study visits heart rate, blood pressure, respiratory rate, temperature, and weight will be measured and a sputum sample will be obtained. If the patient is unable to spontaneously expectorate a sputum sample at any of these times, an attempt will be made to induce sputum production by aerosol inhalation. Study sites will use their local procedure for sputum induction. A sputum sample will be defined as unobtainable (and negative for analytic purposes) if no sputum can be obtained after an attempt at sputum induction. Laboratory tests for toxicity monitoring will also be obtained at study visits during the intensive phase of therapy. These tests will be a complete blood count and serum AST, total bilirubin, and creatinine. Visual acuity and color vision testing, per standard site practice, will be repeated at the 4 week visit. Patients found to have HIV infection, per the result of testing done at screening, will have blood drawn for a CD4 cell count at the week 2 study visit (or at the first study visit after HIV infection is documented.) Patients with documented HIV infection at screening, for whom written results of CD4 testing done within 6 months prior to the start of study therapy cannot be obtained, will have blood drawn for these tests as part of the study at the week 2 study visit. Participants not in routine HIV care will be referred for such care, particularly if the CD4 count is low (i.e. < 200 cells/mm^3^). At each study visit during the intensive phase of therapy, patients will be asked about what medications they have been taking and if they have had any illnesses that would qualify as a new diagnosis or adverse event since the last visit.

End of intensive phase study visit –As above, the end of the intensive phase of therapy is defined by number of doses (40 to 44 doses, as defined in Section 3.1). When a patient has completed the assigned intensive phase, the monitoring blood tests will be obtained (complete blood count and serum AST, total bilirubin, and creatinine). Visual acuity and color vision testing, per standard site practice, will be repeated. In addition, two sputum cultures will be obtained for assessment of the sputum culture conversion, the primary endpoint of the trial. These specimens must be obtained on the day of completing the intensive phase of therapy or up to one week after completion, but before more than one dose of continuation therapy has been given. The two sputum specimens can be obtained on the same day.

- - 1. Study visits – continuation phase of therapy

Month 3 – Patients will have an assessment for delayed toxicity from the intensive phase of therapy. This will consist of a symptom-driven examination, visual acuity and color vision testing per standard site practice, and laboratory testing (complete blood count and serum AST, total bilirubin, and serum creatinine). A sputum specimen will be obtained for culture.

Months 4 through 6 (4 through 9 for those with extended therapy) - Patients will have study visits monthly during the continuation phase of therapy. Sputum cultures will be obtained at each study visit during the continuation phase of therapy until there have been 2 consecutive negative cultures. Laboratory testing, other than sputum cultures, will be at the discretion of the principal investigator. The study will end upon completion of 18 weeks (or 30 weeks for individuals with cavitation on initial chest radiograph plus positive sputum culture at 2 months of treatment) of an ATS/IDSA/CDC continuation phase therapy regimen and a related study visit. At each study visit during the continuation phase of therapy, patients will be asked about what medications they have been taking and if they have had any illnesses since the last visit.

- - 1. Scheduled unblinding of study treatment regimens

The subjects will all be unblinded at one time, under normal circumstances, when the data are complete. This is scheduled for four months after the last patient has completed continuation phase therapy. The protocol team will prepare information they wish site personnel to communicate to study subjects when their treatment assignment is discussed. The protocol statistician will prepare unblinding listings for each site that are sent along with the unblinding memo on the date the protocol team has specified. Section 5.9 describes the procedure for responding to individual patient requests for early unblinding.

- - 1. Compensation for study subjects

Study subjects will be compensated for their time and travel to and from study visits. The form of compensation will be in accordance with local IRB guidelines.

1. **CLINICAL MANAGEMENT ISSUES**
   1. Drug-Susceptibility Testing

All baseline isolates of *Mycobacterium tuberculosis* will undergo drug susceptibility testing at the site’s local laboratory. The baseline isolate will be defined as the first *M. tuberculosis* isolate obtained from a sputum culture; in some instances this sputum specimen may have been obtained prior to study screening. An *M. tuberculosis* isolate cultured in a laboratory other than a site’s study laboratory of record may be designated as the baseline isolate if the isolate is sent to the study laboratory of record. Drug susceptibility testing will include susceptibility to isoniazid, rifampin, and ethambutol. Initial susceptibility testing generally requires 4-8 weeks to complete (initial isolation followed by susceptibility testing). It is expected that patients will be enrolled in this protocol with drug-resistant isolates, most often resistance to isoniazid, with or without streptomycin resistance[109, 110]. Rifampin resistance and multidrug-resistance (resistance to isoniazid and rifampin) is uncommon among previously untreated patients in the United States[109], Uganda[110], Canada[111], and South Africa[112]. All isolates will be sent to the CDC Mycobacteriology Laboratory for confirmatory susceptibility testing; this testing will include all drugs currently used to treat tuberculosis, including pyrazinamide and fluoroquinolones. The confirmatory testing will take several additional months, and these results are unlikely to be available during the first 2 months of TB treatment. All study regimens should be highly active for isolates with susceptibility to isoniazid and rifampin.

- 1. Management of patients with baseline drug-resistant isolates

Isoniazid resistance – Isoniazid resistance does not appear to affect sputum culture conversion, but is associated with slightly higher rates of treatment failure and relapse (Introduction, “Factors other than therapeutic regimen known to affect 2-month culture-conversion”), and current guidelines suggest the use of rifampin, pyrazinamide, and ethambutol for patients with isoniazid-resistant isolates. Patients with isoniazid resistance will be taken off of their assigned study regimen and treated using an individualized regimen at the discretion of the principal investigator. These patients will not be included in the analysis of sputum culture conversion. Patients receiving study (intensive) phase therapy at the time of discontinuation of the study regimen will have a study visit at 2 weeks after discontinuation of the study regimen for assessment of delayed toxicity, and then will no longer be followed in the study. Patients receiving continuation phase therapy at the time of discontinuation will no longer be followed in the study.

Fluoroquinolone resistance – Rates of fluoroquinolone resistance are extremely low in the United States and Canada (0.3% among rifampin-susceptible isolates) [10]and likely to be very low in Africa. Patients having a fluoroquinolone resistant isolate will be taken off their assigned study regimen and treated using an individualized regimen at the discretion of the investigator. These patients will not be included in the analysis of sputum culture conversion. Patients receiving study (intensive) phase therapy at the time of discontinuation of the study regimen will have a study visit at 2 weeks after discontinuation of the study regimen for assessment of delayed toxicity, and then will no longer be followed in the study. Patients receiving continuation phase therapy at the time of discontinuation of the study regimen will no longer be followed in the study.

Pyrazinamide resistance – Isolated pyrazinamide resistance is extremely rare, except among *M. bovis* strains (*M. bovis* is characteristically resistant to pyrazinamide). Patients found to have confirmed pyrazinamide resistance or intolerance will be taken off their assigned study regimen and treated using an individualized regimen. Patients with pyrazinamide resistance or intolerance will not be included in the analysis of sputum culture conversion. Patients receiving study (intensive) phase therapy at the time of discontinuation of the study regimen will have a study visit at 2 weeks after discontinuation of the study regimen for assessment of delayed toxicity, and then will no longer be followed in the study. Patients receiving continuation phase therapy at the time of discontinuation will no longer be followed in the study.

Rifampin resistance, with or without resistance to other drugs – Patients having a rifampin-resistant isolate or rifampin intolerance will be taken off their assigned study regimen and treated using an individualized regimen. Patients with rifampin resistance or intolerance will not be included in the analysis of sputum culture conversion. Patients receiving study (intensive) phase therapy at the time of discontinuation of the study regimen will have a study visit at 2 weeks after discontinuation of the study regimen for assessment of delayed toxicity, and then will no longer be followed in the study. Patients receiving continuation phase therapy at the time of discontinuation will no longer be followed in the study.

Ethambutol resistance – Rates of ethambutol resistance are low in Uganda[110], Canada[111], and the United States[109]. The study regimen should be very active for a patient having an ethambutol-resistant isolate. These patients will be kept on their assigned study regimen and included in the analysis of sputum culture conversion.

Resistance to two or more drugs - Patients with resistance to any two or more of the study drugs, most likely isoniazid and ethambutol or pyrazinamide, will be taken off their assigned study regimen and treated with an individualized regimen at the discretion of the investigator. These patients will not be included in the analysis of sputum culture conversion. Patients receiving study (intensive) phase therapy at the time of discontinuation of the study regimen will have a study visit at 2 weeks after discontinuation of the study regimen for assessment of delayed toxicity, and then will no longer be followed in the study. Patients receiving continuation phase therapy at the time of discontinuation will no longer be followed in the study.

Resistance to a non-study drug, with or without isoniazid-resistance – Rates of resistance to streptomycin are relatively high (7-10%) and often associated with isoniazid-resistance. In that resistance to non-study drugs should not affect the activity of the study drugs, patients with isolates having resistance to non-study drugs will be kept on their assigned study regimen and included in the analysis of sputum culture conversion.

- 1. Study drug toxicity

Patients will be closely monitored for signs and symptoms of study drug toxicity. For all toxicities that are treatment-emergent and that require the study therapy to be temporarily or permanently discontinued, relevant clinical and laboratory tests will be obtained as clinically indicated and repeated as needed until final resolution or stabilization of the toxicity.

If a patient develops hepatic toxicity requiring study drug discontinuation, the following evaluation will be undertaken: assessment for history of injection or noninjection drug use, alcohol ingestion, use of other hepatotoxic drugs, and performance of serologic tests for viral hepatitis (IgM antibody for Hepatitis A, Hepatitis B surface antigen, IgM antibody to Hepatitis B core antigen, antibody to Hepatitis C).

All symptoms and laboratory findings will be graded according to severity using the modified National Cancer Institute Common Toxicity Criteria (Appendix E). For the purposes of this study, pregnancy will be reported on the Adverse Event Form. All pregnant patients will be taken off study therapy and treated using the ATS/IDSA/CDC guidelines for tuberculosis treatment during pregnancy. Women who become pregnant will continue to receive scheduled follow-up, not receive additional x-rays unless clinically indicated, and be classified as being on a non-study regimen. The outcome of the pregnancy will be reported on the Adverse Event Follow-up Form.

- 1. Adverse event reporting

An adverse experience is defined as any unintended or abnormal clinical observation that is not of benefit to the patient. The adverse experiences that must be reported on an Adverse Event Form include: pregnancy, new medical diagnosis, grade 3 or 4 toxicity (potentially medication-related or other causes), study drug discontinuation, medication side-effects that are grade 3 or 4, and serious adverse events (defined in next paragraph). At the time of enrollment, if the patient already has a medical diagnosis whose signs or symptoms worsen during the study to a Grade 3 or 4, this is an adverse event that must be reported.

A serious adverse event (SAE) is defined as any experience that is fatal or life-threatening, is persistently or significantly disabling (as determined by the principal investigator), requires inpatient hospitalization, or prolongation of hospitalization, is a congenital anomaly, or overdose of study drugs. Any Grade 4 toxicity is considered an SAE.

The study drugs are moxifloxacin, ethambutol, isoniazid, rifampin, and pyrazinamide. The most common adverse effects associated with the study drugs are specified in Section 2.2 - "Study Drugs". The investigator is responsible for monitoring all adverse events that are observed or reported during the study, regardless of whether they are related to study drugs.

The TB Trials Consortium (TBTC) adverse experience reporting system has been developed to ensure timely and accurate reporting of adverse experiences in order to monitor patient safety, to comply with Food and Drug Administration (FDA) regulations, and to disseminate information to investigators working with the study drugs. In accordance with the FDA’s Code of Federal Regulations, the sponsor of this clinical trial (CDC) and the participating investigators are responsible for reviewing all information relevant to the safety of the study drugs. Reporting and monitoring of SAEs is required to alert the FDA, sponsor, institutional review boards, and the clinical investigators of real and potential safety issues. **Any Serious Adverse Event (this includes any Grade 4 toxicity) must be reported to the CDC on an Adverse Event Report Form within 48 hours of the site's awareness of the event.**

The CDC DTBE will carefully review the Adverse Event Report and use this information to monitor the investigational drug's toxicity profile and patient safety. Those adverse events, which are serious, unexpected, and related to the study drug, must be reported by the CDC and to the FDA in the form of a written Safety Report. The responsible institutional review board should be notified as soon as possible of any serious or life-threatening adverse experiences.

- 1. Management of adverse events

In general, for grade 1 toxicities, the patient will be followed carefully and the study drugs will be continued. For grade 2 toxicities, the patient will be followed more carefully, with additional laboratory and/or clinic visits as necessary, and the study drugs temporarily held at the investigator's discretion. For any grade 3 toxicity that, in the principal investigator’s judgment is due to study drug(s), the causative study drug(s) should be held. The clinician should rule out other possible causes of the symptoms before discontinuing study medication. When possible, concomitant medications should be held first at the discretion of the principal investigator if he/she suspects they are contributing to the toxicity. Depending on the nature and severity of the toxicity, the degree to which it resolves, and/or the emergence of alternative explanations for the toxicity or the subject’s deterioration, the study drugs(s) may be restarted at the discretion of the investigator. For any recurring grade 3 or grade 4 toxicity, the study drugs should be temporarily held and may be permanently stopped at the discretion of the investigator.

Any patient with grade 4 renal, hepatic, cardiac or hematological toxicity will be immediately discontinued from study therapy. The laboratory test or clinical finding in question will be reassessed as soon as possible. The repeat test will guide management of the event as follows:

- If the repeat assessment shows toxicity of grade 3 or lower, and if the patient has continued to receive study drugs between the two testing dates, then the patient will be managed according to the appropriate toxicity level of the repeat test.
- If the repeat test shows toxicity of grade 3 or lower, and if the patient has not received study drugs between the two testing dates, then the patient will be managed at the discretion of the investigator with regard to the re-administration of study drugs, and otherwise according to the toxicity level of the repeat test.
- If the repeat test shows grade 4 toxicity, then the patient will be permanently discontinued from study medications. Further treatment of TB will be directed by the investigator on an individualized basis. The patient will continue to be followed for study monitoring purposes (as are other patients who make a permanent departure).

For other grade 4 toxicities, the study drugs will be temporarily held and may be restarted or permanently stopped at the discretion of the investigator. Toxicities graded 3 or 4 by the Common Toxicity Criteria and occurring during study therapy will be documented on the Adverse Event Form, according to the criteria described in the next three sections. The maximum level of toxicity reached will be clearly indicated.

- 1. Criteria for discontinuation of study drugs

Certain events or conditions may necessitate temporary or permanent discontinuation of the study medication. Patients who experience such events or conditions, however, will still be "on study" and will be followed until study completion. Any patient for whom the study medication is temporarily discontinued will be restarted on study medication as soon as possible. Study regimens will be discontinued and non-study regimens will be used with continued study followup for patients who fail to respond to study therapy either clinically and/or bacteriologically, for patients in whom treatment-emergent drug toxicity warrants discontinuation of study therapy, and for patients who become pregnant. If study drugs are permanently discontinued, further antituberculous therapy may be administered at the investigator's discretion. These patients will be followed in the study for a maximum of 6 months of TB treatment, according to the guidelines and time-points established in the protocol, starting from the date of initiation of Study 28 intensive phase treatment.

- 1. Criteria for temporary discontinuation of study therapy
- Development of a toxicity that, depending on its nature and severity, requires temporary discontinuation of the study medication until the toxicity resolves as indicated in the preceding toxicity management section.
- Development of another medical condition that makes the administration of the study drug inadvisable. The decision to temporarily discontinue the study medication in this situation will be at the investigator's discretion. The period during which the patient is off study medication will be as short as clinically possible.
  1. Criteria for permanent discontinuation of study therapy
- Development of a toxicity that warrants permanent discontinuation of any study drug (refer to section on “Management of Adverse Events”).
- The patient refuses further therapy.
- It is the investigator's judgment that it is no longer in the best interest of the patient to continue study therapy.
- Termination of the study.

If a patient refuses further therapy or withdraws consent for treatment under the protocol, the patient will be treated with a non-study regimen. If the patient has withdrawn consent, all follow-up will stop.

- 1. Unscheduled unblinding of study assignment

# The need to unblind a patient’s study assignment should be rare in this trial. Patients with serious adverse events that are thought due to study therapy will have reached a study endpoint and will be taken off blinded study medication and then treated at the discretion of the principal investigator. Three of the study medications – rifampin, pyrazinamide, and ethambutol - are open-label. Moxifloxacin is not a part of standard tuberculosis treatment, but there should rarely be a need to know if this drug caused a serious adverse event. Isoniazid can cause drug-induced hepatitis, so there may be a need to unblind study medications if a patient has had significant hepatotoxicity. An investigator who believes that knowledge of study assignment is important in developing a study regimen for a patient who has met the criteria for study drug discontinuation can request unblinding.

# A request for individual unblinding should be made in writing (including Email) by the principal or co-investigator to the Data and Coordinating Center. A team, consisting of the protocol chair and co-chairs, and the study project officer will review these requests within two working days of receipt. If unblinding is approved, only those persons at the local study site who need to know this information to develop a safe treatment regimen should be informed of the unblinding.

# A code-break card for each patient kit randomization number will be provided to the investigative sites with each patient kit of study medication. The code break card should be maintained by study personnel and should be kept in a secure location. The code-break card contains the identity of the randomization arm for its corresponding kit and can be opened for emergency purposes only. However, whenever possible, the investigator should contact the principal or co-investigator or the Data and Coordinating Center, as described above, prior to breaking the code. Investigators should note that the occurrence of a serious adverse event (SAE) should not routinely trigger unblinding. If the code is broken, the Data and Coordinating Center must be notified by telephone immediately. The date and reason for the code break must be documented on the adverse event form. Code-break cards will be inventoried and inspected for intactness at each twice-yearly site visit by the study monitors.

- 1. Concomitant medications during study phase

The use of all non-study drugs (including over-the-counter medications) from 14 days before starting study therapy through the end of study phase therapy will be monitored and recorded. In addition, if an AE or SAE occurs within 14 or fewer days after completion of study phase therapy, then the use of all non-study drugs (including over-the-counter medications) during the interval from study phase completion to AE/SAE onset will be recorded. Patients receiving study phase therapy at the time of early permanent departure, and who will no longer be followed, will have a study visit at 2 weeks after discontinuation of the study regimen for assessment of delayed toxicity. The use of all non-study drugs (including over-the-counter medications) will be recorded at this final study visit.

Concomitant antimicrobials with known antituberculous activity (rifabutin, rifapentine, amoxicillin/clavulanic acid, amikacin, kanamycin, streptomycin, ofloxacin, ciprofloxacin, levofloxacin, sparfloxacin, gatifloxacin, gemifloxacin, linezolid, capreomycin, cycloserine, ethionamide, para-aminosalicylate sodium) should not be used during the study phase therapy. Any patient who receives more than 7 consecutive days of any of the medications listed above will be classified as being on a non-study regimen. Antimicrobials with no known antituberculous activity may be prescribed for intercurrent infections at the discretion of the investigator.

Moxifloxacin does have the potential to prolong the QT interval, a class property of the fluoroquinolones. Quinidine, procainamide, sotalol, amiodarone, disopyramide, terfenadine, and ziprasidone are contraindicated for use with moxifloxacin. If a patient requires therapy with one of these agents, they will be taken off blinded study medication and alternate TB treatment will be prescribed by the principal investigator. Other drugs that may have an effect on QT interval should be used with caution during blinded study treatment: cisapride, erythromycin, clarithromycin, perphenazine, phenothiazines, and tricyclic antidepressants.

Isoniazid decreases the clearance of some medications that are metabolized in the liver. Patients who take isoniazid and require the following medications will have appropriate serum drug levels measured to prevent toxicity: carbamazepine, phenytoin, and theophylline. Antacids that contain aluminum and/or magnesium and antidiarrheals that contain kaolin can decrease isoniazid, ethambutol, and moxifloxacin absorption. Study patients requiring the use of antacids containing aluminum and/or magnesium, sucralfate, antidiarrheals that contain kaolin, or iron and/or zinc-containing medications or supplements will need to take their study drugs at least 4 hours before or 8 hours after ingesting these products to avoid impaired absorption of study drugs.

Rifampin induces a number of hepatic enzyme systems that are active in the metabolism of many drugs. The most common site of rifampin-related interactions is the cytochrome CYP450 enzyme system. Suggestions for the management of rifampin-related drug interactions are given in Appendix C. A prominent drug interaction of rifampin is that involving hormonal contraceptives. Women of childbearing potential (i.e., not surgically sterilized or postmenopausal for less than 1 year) will be advised to use one or more of the following contraceptive methods: intrauterine device; barrier methods including the diaphragm or cervical cap (each used with spermicidal foam or jelly), condom, and sponge; or abstinence.

Ethanol can exacerbate the potential hepatotoxicity of isoniazid, rifampin, and pyrazinamide. Participants will be urged to abstain from alcohol while on study phase therapy.

- 1. HIV management

HIV testing will be done by trained personnel who use approved procedures for pre-test and post-test counseling. HIV test results will be given only to the patient (and to those to whom it may be required by law) and kept with other study records in a secure place. Patients found to have HIV infection, per the result of testing done at screening, will have blood drawn for a CD4 cell count at the week 2 study visit (or at the first study visit after HIV infection is documented). Patients with documented HIV infection at screening, for whom written results of CD4 testing done within 6 months prior to the start of study therapy cannot be documented, will have blood drawn for this test as part of the study at the week 2 study visit. These tests will be performed at experienced laboratories and the results will be given to patients as soon as they are available (generally within two weeks). HIV-positive patients will be referred to local sources of HIV care, as appropriate.

The protocol does not allow the use of antiretroviral therapy during the intensive phase of therapy (first 2 months) because of overlapping side effect profiles of antituberculosis and antiretroviral drugs, the adherence challenge of starting so many drugs in a short period of time, the complex drug-drug interactions between rifamycins and many antiretroviral drugs (most HIV-1 protease inhibitors and non-nucleoside reverse-transcriptase inhibitors)[113], and the possible occurrence of severe immune reconstitution syndromes when antiretroviral therapy is started early in the course of tuberculosis treatment[114]. Patients whose clinical condition is thought to require antiretroviral therapy during the first two months of TB treatment should not be enrolled (see Exclusion criteria).

- 1. Management of patients with tuberculosis treatment failure

Patients with treatment failure (see section 3.5) will be managed with an individualized treatment regimen at the discretion of the investigator. For each patient with treatment failure, drug susceptibility testing at the CDC will be performed for one *M. tuberculosis* isolate from sputum obtained after completion of 4 months of therapy. This drug susceptibility test result will be compared with that of the patient’s initial isolate. Patients with treatment failure will be followed in the study for a maximum of 6 months of TB treatment, according to the guidelines and time-points established in the protocol, starting from the date of initiation of Study 28 intensive phase treatment.

1. **EVALUATION**
   1. Data analysis

Comparison of baseline characteristics of treatment and control groups using chi-square test for categorical variables and t-test for continuous variables will be undertaken to evaluate for potential patient differences. Analysis of primary and secondary endpoints will be analyzed primarily as proportions and compared using chi-square or Fisher’s exact test, as appropriate. Multivariate proportional hazards regression will be used to identify independent risk factors for sputum culture conversion, and to adjust for any differences in baseline characteristics.

Since a percentage of subjects may drop out or be lost to follow-up in our clinical trial, imbalances may result that "undo" the effects of randomization. If there were no dropouts or subjects lost to follow-up, the effects of randomization would tend to make the experimental and control arms comparable with respect to possibly important predictors of the endpoint of interest. However, if there are drop-outs or subjects lost to follow-up, the two treatment groups may not be directly comparable at the end of the follow-up period, particularly if dropping out or being lost to follow-up are associated with the study regimen assignment or other potentially important predictors of the endpoint. Therefore, we plan to look at these subjects for potential differences in comparison to those who remained in the study.

Sputum culture conversion within 70 days is the measure of the sterilizing activity of the study regimen in this trial. The following patients will be removed from the analysis of sputum culture conversion (following the analytic procedures of the British Medical Research Council studies of 2-month culture-conversion): 1) patients who take non-study therapy for more than 14 days during the intensive phase of therapy, 2) patients who require more than 70 days to complete the intensive phase, 3) patients who die during the intensive phase of therapy, 4) patients with no analyzable sputum cultures (e.g. overgrown with bacteria or yeast), and 5) patients whose baseline isolate is resistant to isoniazid, rifampin, pyrazinamide, moxifloxacin, or any two study drugs. Culture conversion will be analyzed in two different ways. The primary analysis will compare proportions of patients with negative cultures (patients with negative cultures/all patients eligible) using the chi-square or Fisher’s exact test, as appropriate. In a secondary analysis, the time to sputum culture conversion will be compared with non-parametric methods, using the results of sputum cultures at 2, 4, 6, and 8 weeks of therapy.

All patients randomized will be included in the analyses of toxicity.

The TBTC Data and Safety Monitoring Board (DSMB) will review the study protocol and oversee progress of the trial once a year. Special meetings of the DSMB may be called if unusual adverse events are noted. The DSMB will be comprised of a TB expert, an HIV/AIDS expert, and a statistician who are not otherwise involved in the study. Lan and Demets methodology to construct discrete sequential boundaries will be used to assess the sputum conversion rates at the time of each DSMB analysis; this approach has been adopted in many studies for maintaining the overall significance level when repeated looks are made to assess significance[115]. We will use an O’Brien-Fleming “spending function” approach to decrease the probability of erroneously concluding that the experimental regimen has statistically significant efficacy[116].

The DSMB will be given data grouped by study group (A or B), but will have access to unblinded data if requested.

1. **ADMINISTRATIVE ISSUES**
   1. Supply of study drugs

Moxifloxacin and matching placebo will be provided by Bayer Corporation. Moxifloxacin will be formulated either as an encapsulated 400 mg tablet, or as a bioequivalent nonencapsulated 400 mg tablet, with appropriate matching placebo. An individual patient will receive the same formulation throughout their study phase treatment Isoniazid ( 3 scored 100 mg tablets) and matching placebo tablets will be obtained by CDC. Moxifloxacin, isoniazid, and matching placebo tablets will be distributed by a contract research organization; study sites will supply ethambutol, rifampin, pyrazinamide, and vitamin B6. Sites will have a supply of moxifloxacin, isoniazid, and matching placebos because study therapy should be started as soon as possible after randomization.

Ethambutol, rifampin, and pyrazinamide used at North America sites will be from batches approved by the U.S. Food and Drug Administration. Ethambutol, rifampicin and pyrazinamide that will be used at study sites in Spain, South Africa, Uganda, and Brazil will be purchased from distributors/manufacturers using Good Manufacturing Practice Regulations.

- 1. Drug accountability and record keeping

The investigator will acknowledge receipt of and keep an inventory of moxifloxacin, isoniazid, and matching placebos. Study staff has the responsibility to assure that study drugs are dispensed to patients in compliance with the protocol.

1. **TBTC STUDY SITES**

The TBTC conducts programmatically relevant research on TB treatment. At present, the Consortium has sites in the United States, Canada, Brazil, Spain, South Africa, and Uganda. All sites have close connections with the local TB control program; some sites are based in the TB control program. All sites work with experienced mycobacterial laboratories, and the CDC’s Mycobacteriology Laboratory serves as central laboratory for confirmation of drug-susceptibility testing, DNA fingerprinting, and further characterization of drug-resistant isolates.

Since its inception in 1994 over 4500 patients have been enrolled in TBTC clinical trials, including several done under Investigational New Drug applications.

1. **HUMAN SUBJECTS PROTECTION**
   1. Ethical issues in doing this trial in developing and developed countries

The vast majority of the global tuberculosis burden occurs in developing countries[2]. New treatment regimens that substantially improve tuberculosis treatment (shorten and/or simplify therapy) are critically needed in these settings. Therefore, it is appropriate to evaluate a new drug for tuberculosis treatment in a developing country setting.

This is a Phase 2 trial involving a new drug for tuberculosis treatment. If moxifloxacin proves to be safe and to increase 2-month culture-conversion rate, its role in tuberculosis treatment would still need to be evaluated in one or more larger Phase 3 trials. Therefore, this trial, by itself, is not expected to change recommendations for tuberculosis treatment in either developed or developing countries. However, there are a number of initiatives in place to assure that moxifloxacin, or a comparably active fluoroquinolone, would be available in developing countries should it substantially improve tuberculosis treatment (e.g., allow shortening of therapy).

Centers for Disease Control and Prevention (CDC) is a signatory to the *Cape Town Declaration* [117] and a major partner of the Global Alliance for TB Drug Development[118]. As such, CDC has pledged the resources of the TBTC as a public good in pursuit of the mission of the Global Alliance for TB Drug Development, including equitable access to new and improved TB treatments. The Global Alliance for TB Drug Development has played a facilitating role in the CDC collaboration with Bayer Pharmaceuticals for this study. CDC is also actively engaged with the Stop TB Partnership, having a seat on its Board of Directors[119]. The Partnership’s Global TB Drug Facility, **a mechanism to expand access to, and availability of, high-quality TB drugs to facilitate global expansion of directly observed therapy-short course (DOTS) programs, would provide fluoroquinolones should they prove to be effective for treatment of drug-susceptible tuberculosis. CDC is a member of the Stop TB DOTS-Plus Working Group and sits on its “Green Light Committee” that provides low-cost, high-quality second-line TB drugs (including fluoroquinolones) to programs implementing MDR TB treatment under the expanded DOTS strategy[120]. Finally, the Global Fund to Fight, AIDS, Tuberculosis, and Malaria is providing direct financial support to enable National Tuberculosis Control Programs in low-income countries to purchase high-quality TB drugs[121]. All these factors suggest that future access to affordable new TB drugs will be a reality once they are developed and their role in TB treatment established.**

- 1. Ethical issues involving persons who are incarcerated

This trial will not enroll persons who are incarcerated. However, it is possible that a participant will be incarcerated after enrollment. If this occurs during the first two months of treatment, the study medications will be stopped and the participant will be treated for active tuberculosis according to the standards of the institution in which they are incarcerated. However, in the interest of describing the toxicity of the study medications as fully as possible the study will try to obtain follow-up safety data on participants who are incarcerated. Whenever possible, participants who are incarcerated during intensive and/or continuation phase will continue to be followed by study personnel via contact with the appropriate authorities.

- 1. Institutional review board involvement

The protocol and the informed consent document to be used in this study must be submitted to the investigator’s local ethics review committee/IRB and the CDC IRB for approval. Written documentation of approval of both the protocol and the informed consent must be provided to CDC before starting the study.

The investigator will ensure that the purpose of the study is explained to the patient and that written consent is obtained prior to participation in the study. The patient, investigator, and others as required by local regulatory guidelines will sign the consent prior to entry into the study. The patient will receive a copy of his/her signed consent form. The investigator will retain a copy of the signed consent forms, which may be inspected at the monitor’s or auditor’s request. Obtaining informed consent from vulnerable populations (e.g., persons who do not read) will follow guidelines established by local and CDC ethics committees/IRBs.

The investigator will promptly report to the Ethics Committee/IRB of all changes in the research activity and all unanticipated problems involving risks to human subjects or others, and will not make changes in the research without Ethics Committee/IRB approval, except where necessary to eliminate apparent immediate hazards to human subjects.

1. **REFERENCES**
2. **Appendix A** – Sample informed consent (Flesch-Kincaid Reading Grade Level 7.3)

# Evaluation of a Moxifloxacin-Based, Isoniazid-Sparing Regimen for Tuberculosis Treatment

Consent for Research

**Introduction.**

You are being asked to be in this research study because your doctors believe you have tuberculosis (TB). The *(name of study site)* and the Centers for Disease Control and Prevention (CDC) are working together on this study.

**Why is this study being done?**

The purpose of this study is to find out whether the medicine called moxifloxacin is more active against TB than another medicine called isoniazid (INH) during the first 2 months of TB treatment. The study will also see what side effects are caused by taking moxifloxacin for 2 months.

Treatment for TB usually includes 4 medicines, isoniazid (INH), rifampin, pyrazinamide (PZA), and ethambutol. In this study, we will study a new medicine called moxifloxacin for TB treatment. Moxifloxacin is safe and effective for treatment of some bacterial infections. Because of this, it was approved by the Food and Drug Administration (FDA). Moxifloxacin has not been tested for treatment lasting longer than 2 weeks and is not a part of standard TB treatment.

**How many people will take part in this study?**

About 410 people will be in this study. Enrollment into the study will take about 2 years.

**How long will i be in the study?**

- 6 months of TB treatment

*OR*

- 9 months of TB treatment if your chest x-ray at the time you are diagnosed with TB shows lung damage called “cavitation” and culture of your sputum (material that you cough up from your lungs) still has TB in it after 2 months of treatment
- We will take you out of the study if:
- your first sputum cultures for TB are negative (do not grow TB) or
- your TB is resistant to some of the TB medicines.

**What is involved in the study?**

If you agree to be in this study, it will change your TB care in the following ways:

- TB treatment starts with 4 different medicines. Everyone in this study will get three of the standard medicines for TB treatment – rifampin, PZA, and ethambutol. Half of the people in this study will get the 4^th^ standard medicine, called INH. The other half will get the new medicine, called moxifloxacin.
- You will have a test for HIV (Human Immunodeficiency Virus, the virus that causes AIDS), using about 1 teaspoon (5 cc) of blood drawn from a vein. This is a recommended test for everyone with TB and is required for this study. If you have had a negative HIV test within 6 months or less (and we can get the written result), you do not need another test. If you are HIV positive, we need a copy of your HIV-positive test results for this study. We will keep the test results private as much as the law allows.
- You will have a pregnancy test, if you can get pregnant.
- You will have blood tests (using about 2 teaspoons (10 cc) of blood drawn from a vein) to check your liver, kidneys, and blood count. You will have the tests before starting the study, unless we can get the results from tests done within 14 days or less. You will also have the tests every 2 weeks during the first 2 months of the study.
- You will be asked questions about your health, such as whether you have had past treatment for TB, and what medicines you are taking. We will ask about any other illnesses and if you drink alcohol or take drugs. We will ask about other risk factors for TB. Some of these questions are personal and you do not have to answer them if you do not want to.
- Your weight and height will be checked.
- You will have your vision tested.
- You will have sputum tests before starting the study and every 2 weeks during the first 2 months of the study. Sputum is material coughed up from the lungs and windpipe. Sputum tests are important in this study. They will tell us if the new medicine is working to treat TB. If you are not able to cough up anything, we will have you breathe in a mist of moist air with sterile salt water in it. This may help loosen any phlegm in your lungs so you can cough it up. This is not painful. It takes about 15 minutes.
- An x-ray of your lungs will be done unless we have the results of one done within 14 days.
- In this study, you cannot take certain medicines during the first 2 months of TB treatment. This includes medicines to treat HIV infection.

**Study Treatment.**

You will be assigned to one of 2 study treatment groups. Neither you nor the research team will choose which group you are in. The treatment group for you will be chosen by chance like flipping a coin. The study treatment groups to which you might be assigned are:

- **8 weeks of daily** **standard TB medicines** (INH, rifampin, PZA, ethambutol) plus moxifloxacin placebo (harmless pill – like a sugar pill)

*OR*

- **8 weeks of daily moxifloxacin plus 3 standard TB medicines** (rifampin, PZA, and ethambutol) and isoniazid placebo (harmless pill – like a sugar pill)

In the first 14 days of treatment, you will get the medicines for 5 to 7 days a week. After that, you will get the medicines 5 days per week (Monday through Friday). Everyone in the study will get Vitamin B6 with each dose of TB medicines. This decreases the chance of numbness and/or tingling in the arms and legs when taking TB medicines. You should take your TB medicines at least 4 hours before or 8 hours after taking antacids, medicine for diarrhea, or medicine with iron or zinc because these other medicines may keep the TB medicines from working. Examples of medicine with iron or zinc are iron tablets and multivitamins with minerals.

A health care worker or treatment supervisor will give you each dose of the study treatment medicines. The medicines may be given at the TB clinic or other health facility. Or, if you agree, the medicines may be given at your home, workplace, or other convenient location. During the 8 weeks you are taking the study treatment, you will:

- Meet with a study doctor or nurse at 2, 4, 6, and 8 weeks after you start the study. These study visits will take about 30 minutes
- Have blood tests to check your liver, kidneys, and blood count. We will draw about 2 teaspoons (10 cc) of blood from a vein for these tests.
- Have your sputum tested for TB. At the 8 week study visit, you will have 2 sputum tests.
- Be asked if you have taken any other medicines or had any illnesses since we last met.
- Have your vision tested at weeks 4 and 8.
- (If you are HIV positive), have about 3 teaspoons (15 cc) of blood drawn for a CD4 (T-cell) count. This will be done at the week 2 study visit (or at the first study visit after we receive positive HIV test results). We will not do the CD4 count test if we can get a written copy of this test result from anytime in the 6 months before you started the study medicines. We will give you the result at the first study visit that we have it.

If you are in prison or jail during this part of the study, we will stop the study medicines but you will continue to be followed as part of the study. You will be treated with standard TB medicines by the doctor who takes care of you while you are in prison or jail.

**Follow-up.**

This part of the study starts when you finish taking the study medicines (the first 8 weeks with 4 medicines plus a placebo). Your TB treatment will continue until you have had a total of 26 weeks (6 months) or 38 weeks (9 months) of treatment. Your doctor or health department will decide your TB treatment in the follow-up part of the study. Your part in the study ends when you finish TB treatment.

During the follow-up part of the study, you will meet with a study doctor or nurse once each month while you are still on TB treatment. These study visits will take about 20 minutes. At the first follow-up visit, you will:

- Have blood tests to check your liver, kidneys, and blood count. We will draw about 2 teaspoons (10 cc) of blood from a vein for these tests.
- Have a test of your sputum to see if the TB is responding to treatment.
- Have your vision tested
- Be asked if you had taken any medicines or had any illnesses since we last met.

At the other follow-up visits, you will:

- Have a test of your sputum to see if the TB is responding to treatment.
- Be asked if you had taken any medicines or had any illnesses since we last met.

Study Schedule

| Procedures | Screening | At 2 weeks | At 4  weeks | At 6  weeks | At 8  weeks | At 3  months | At 4  months | At 5  months | At 6  months * |
| --- | --- | --- | --- | --- | --- | --- | --- | --- | --- |
| Medical history | X | X | X | X | X | X | X | X | X |
| Vision test | X |  | X |  | X | X |  |  |  |
| Blood tests | X | X | X | X | X | X |  |  |  |
| Sputum test | X | X | X | X | X | X | X | X | X |
| Length of visit (minutes) | 45 | 30 | 30 | 30 | 30 | 20 | 20 | 20 | 20 |

*If you take medicines for 9 months, you will also have study visits at 7, 8, and 9 months. These visits will be like the visit at 4 months.

If you move away while you are in the study, we will still continue to follow you. If we cannot see you in person, we will contact your new doctor or clinic, or the health department in your area, for help in carrying out study visits. The study visits will be the same as described in this consent form. Dr. *(name of PI at your site)* will continue to be in charge of the study work in your new location.

If you are in prison or jail while you are in the follow-up phase of the study, we will still continue to follow you. If we cannot see you in person, we will contact the medical staff at the facility where you might be, for help in carrying out study visits. The study visits will be the same as described in this consent form. Whether you decide to stay on the study while in jail or prison will not affect your living conditions in jail or prison, or your chance for parole. Dr. *(name of PI at your site)* will continue to be in charge of the study work even if you are in prison or jail during the follow-up phase of the study.

**What are the risks of the study?**

All medicines have possible side-effects. Most people who take the study medicines do not get these problems. Some medicines should not be taken with TB medicines. You should tell the study nurse or doctor about all the medicine you are taking. You should talk with them before you start any new medicine during your TB treatment. This includes even over-the-counter medicine such as antacids, Tylenol, or any other over-the-counter medicines.

***Possible side effects from the new medicine, moxifloxacin***

*Common side effects (1-7%)*

- Upset stomach, nausea, vomiting, diarrhea, dizziness, changes in taste

*Rare, but serious side effects (less than 1%)*

- Tendon swelling, pain, or rupture
- Allergic reaction – rash and fever

***Possible side effects from standard TB medicines***

*Common side effects (5-10%)*

- Upset stomach, poor appetite, nausea, vomiting, diarrhea
- Joint pains
- Mild, itchy skin rash
- Rifampin can make your tears, sweat, saliva, feces, and urine turn orange. Contact lenses may be stained forever due to the orange color of your tears.

*Rare, but serious side effects (less than 1%)*

- Loss of vision. Loss of vision may last for a short time or be permanent. We will check your vision before you start the study and 3 times in the first 3 months of the study.
- Inflammation of the liver (hepatitis) – you might notice yellow eyes, very poor appetite, nausea, vomiting, or pain in the right side of your abdomen. If the study medicine is stopped because of hepatitis, we will do a blood test for viral infections that can cause liver inflammation.
- INH can cause seizures or trouble thinking.
- Allergic reaction (rash and fever) to any of the standard medicines. Rarely, an allergic reaction can cause low blood cell counts or kidney damage.

There may be other side effects from these medicines that we don’t know about yet.

If you have side effects you must tell your study doctor or nurse right away. If that happens you will be evaluated and told what to do. For serious side effects you might have some blood tests. You may be taken off the study medicine. Your TB doctor will then decide what treatment is best for you.

**Risks from drawing blood**

There are a few small risks from having blood drawn. These include brief pain from the needle stick, bruising, bleeding, lightheadedness, and rarely, infection where the needle enters the vein.

**Risks from an HIV test**

HIV testing includes counseling before and after the test. You will be told the test result. You will be told the meaning of the test result, whether it is positive or negative. Some people who have an HIV test might feel anxious. If you feel this way, you can talk about it with (*name of PI at your site*) by calling (*telephone number*). Positive HIV results are reported to (*institution; insert other site specific information about reporting HIV results.)* We will keep HIV test results private as much as the law allows, but there is a small risk of loss of confidentiality about HIV test results.

**Information for Women.**

We do not know enough about the safety of PZA or moxifloxacin for pregnant women or women who are breastfeeding their child. Therefore, you will not be allowed to take part in this study if you:

- are now pregnant,
- plan to become pregnant during TB treatment, or
- are breastfeeding.

If you can get pregnant, you will get a pregnancy test before starting this study. The test must be negative before you can start this study.

Rifampin can make birth control pills, hormone injections, and implants not work for the prevention of pregnancy. Therefore, while you are in the study, you must use some other kind of birth control such as a diaphragm or cervical cap (each used with spermicidal foam or jelly), condom, birth control sponge, or intrauterine device). You need to use one of these kinds of birth control instead of, or along with, birth control pills, hormone injections, or hormonal implants. Not having sex is also a way to avoid pregnancy while in the study.

If you become pregnant during the study, you must tell the study doctor or nurse right away. The study treatment will then be stopped. Your doctor will decide what TB treatment is best for you. No x-rays for the study will be done during pregnancy.

**Benefits.** There are no direct benefits for you for taking part in this study. Your taking part in this research will help us find out if moxifloxacin can improve TB treatment.

**Confidentiality.** We will check your medical records to get information for the study. We will not use your name in any speech or paper about the study. We will not send your name to the study’s data center (CDC). The FDA and study monitors from CDC may also check your records. We will keep all information from your medical records private as much as the law allows.

**Costs And Payment For Being in The Study.** There is no cost to you for being in the study. You will not have to pay for any medicine or tests that are part of this study. *(Individual site compensation information should be included here.)*

**In Case Of Injury.** (*Insert wording required by the site.)* If you are hurt because you are taking part in this study, treatment *will/will not* be provided by ______ *(institution)*. CDC/ _______ *(institution)* does not normally pay for harm done to you as a result of being in a research study. You (or your insurance company, Medicare, or Medicaid) will be billed for this care. By signing this consent form and agreeing to be in this study, you are not giving up any of your rights. If you believe that you have been harmed, please contact _________ (*name and institution*) by calling ______ (*telephone number*) for information on your rights and advice on how to proceed.

**Right To Refuse And Reasons for Withdrawal.** Taking part in this study is voluntary. If you choose not to take part, it will not change your regular medical care. If you choose to be in the study, you may quit at any time without changing your regular medical care. If you quit the study, you will still be treated for TB. If important new information is found that might change your decision to be in the study, we will tell you.

We may stop the study medicine if:

- you have bad side effects,
- the study doctor decides it is best for you to stop the medicine,
- the study medicine is not working against your TB,
- you do not take the medicines like you should, or
- the study ends.

Even if the study medicines are stopped, we will still follow you in the study for a total time of 6 months.

**Alternative treatment.** If you do not take part in this study, you will be treated for TB with the standard medicines.

**Persons To Contact.**  If you:

- Have questions about this research study, contact ______ (*name and institution*) by calling ______ (*telephone number*).
- Have questions about your rights as a research subject, contact _______ (*name and institution*) by calling ______ (*telephone number*).
- Think you may be having a problem from any of the medicines used in this study or feel that you have been harmed by this study, contact ______ (*name and institution*) by calling ______ (*telephone number*).

**Consent Statement.**  “My signature below indicates that I agree to be in this study. I was given a chance to ask questions. I feel that my questions have been answered. I know that being in this study is my choice. I know that after choosing to be in this study, I may withdraw at any time. I have been told that I will receive a signed copy of this consent.”

Signature of subject: __________________________________ Date: ___________

Signature of person obtaining consent: ____________________ Date: ___________

Signature of principal investigator: _______________________ Date: ___________

1. **Appendix B** – Karnofsky performance scale

| Able to carry on normal activity | 100 | Normal; no complaints; no evidence of disease |
| --- | --- | --- |
|  | 90 | Able to carry on normal activity; minor signs or symptoms of disease |
|  | 80 | Normal activity with effort; some signs or symptoms of disease |
| Unable to work; able to live at home and care for most personal needs; a varying amount of assistance is needed | 70 | Cares for self; unable to carry on normal activity or to do active work |
|  | 60 | Requires occasional assistance but is able to care for most of his/her needs |
|  | 50 | Requires considerable assistance and frequent medical care |
| Unable to care for self; requires equivalent of institutional or hospital care; disease may be progressing rapidly | 40 | Disabled; requires special care and assistance |
|  | 30 | Severely disabled; hospitalization is indicated although death is not imminent |
|  | 20 | Very sick; hospitalization necessary; active supportive treatment is necessary |
|  | 10 | Moribund; fatal process progressing rapidly |
|  | 0 | Dead |

1. **Appendix C** – Clinically significant drug-drug interactions involving rifamycins

| **Drug class** | **Drugs affected by the rifamycins** | **Comments** |
| --- | --- | --- |
| Anti-infectives | HIV-1 protease inhibitors | Only ritonavir can be given with rifampin |
|  | Delavirdine, efavirenz | Delavirdine should not be used with rifampin; the dose of efavirenz should be increased to 800 mg |
|  | Macrolide antibiotics (clarithromycin, erythromycin) | Azithromycin has no significant interaction with rifamycins |
|  | Doxycycline | May require use of an alternate drug or drug combination |
|  | Azole antifungal agents | Fluconazole can be used with rifampin; itraconazole and ketoconazole should not be used |
|  | Atovaquone | Consider alternate form of *Pneumocystis carinii* treatment or prophylaxis |
|  | Chloramphenicol | Consider an alternative antibiotic |
|  | Mefloquine | Consider alternate form of malaria treatment or prophylaxis |
| Hormone therapy | Ethinylestradiol, norethindrone | Women of reproductive potential on oral contraceptives should be advised to add a barrier method of contraception when on rifampin |
|  | Tamoxifen | May require alternate therapy |
|  | Levothyroxine | Monitoring of serum TSH recommended, may require increased dose of levothyroxine |
| Narcotics | Methadone | Rifampin use may require methadone dose increase |
| Anticoagulants | Warfarin | Monitor prothrombin time, may require 2-3 fold dose increase |
| Immuno-suppressive agents | Cyclosporine, tacrolimus | These drugs should not be used with rifampin |
|  | Corticosteroids | Monitor clinically, may require 2-3 fold dose increase |
| Anticonvulsants | Phenytoin, lamotrigine | Therapeutic drug monitoring recommended; may require dose increase |
| Cardiovascular agents | Verapamil, nifedipine, diltiazem * | Clinical monitoring recommended, may require change to an alternate drug |
|  | Propranolol, metoprolol | Clinical monitoring recommended, may require dose increase or change to an alternate drug |
|  | Enalapril, losartan | Monitor clinically, may require a dose increase or use of an alternate drug |
|  | Digoxin (among patients with renal insufficiency), digitoxin | Therapeutic drug monitoring recommended; may require dose increase |
|  | Quinidine | Therapeutic drug monitoring recommended; may require dose increase |
|  | Mexiletine, tocainide | Clinical monitoring recommended, may require change to an alternate drug |
| Theophylline | Theophylline | Therapeutic drug monitoring recommended; may require dose increase |
| Sulfonylurea hypoglycemics | For example - glyburide | Monitor blood glucose, may require dose increase or change to an alternate drug |
| Hypolipidemics | Simvastatin, fluvastatin | Monitor hypolipidemic effect, may require use of an alternate drug |
| Psychotropic drugs | Nortriptyline | Therapeutic drug monitoring recommended; may require dose increase or change to alternate drug |
|  | Haloperidol, quetiapine | Monitor clinically, may require a dose increase or use of an alternate drug |
|  | Benzodiazepines, zolpidem, buspirone | Monitor clinically, may require a dose increase or use of an alternate drug |

1. **Appendix D –** Time events schedule

|  |  |  | Week | | | | Month | | | |  | | |  |
| --- | --- | --- | --- | --- | --- | --- | --- | --- | --- | --- | --- | --- | --- | --- |
| Event | Screen-ing | Enroll-ment | 2 | 4 | 6 | 8 | 3 | 4 | 5 | 6 | 7 ^9^ | 8 ^9^ | 9 ^9^ | |
| Clinical |  |  |  |  |  |  |  |  |  |  |  |  |  | |
| Informed consent | X |  |  |  |  |  |  |  |  |  |  |  |  | |
| Medical history | X |  |  |  |  |  |  |  |  |  |  |  |  | |
| Height^10^, weight, vital signs | X | X | X | X | X | X |  |  |  |  |  |  |  | |
| Concomitant medications | X | X | X | X | X | X | X | X | X | X | X | X | X | |
| Adverse events |  | X | X | X | X | X | X | X | X | X | X | X | X | |
| Visual Acuity & Color Vision testing ^1^ | X |  |  | X |  | X | X |  |  |  |  |  |  | |
|  |  |  |  |  |  |  |  |  |  |  |  |  |  | |
| Laboratory |  |  |  |  |  |  |  |  |  |  |  |  |  | |
| Sputum smear and culture | X ^2^ |  | X | X | X | X ^7^ | X | X ^8^ | X ^8^ | X ^8^ | X ^8^ | X ^8^ | X ^8^ | |
| Pregnancy test (urine or serum) ^3^ | X |  |  |  |  |  |  |  |  |  |  |  |  | |
| HIV test ^4^ | X |  |  |  |  |  |  |  |  |  |  |  |  | |
| Complete blood count | X ^5^ |  | X | X | X | X | X |  |  |  |  |  |  | |
| Creatinine, bilirubin, AST | X ^5^ |  | X | X | X | X | X |  |  |  |  |  |  | |
| Serum potassium | X ^5^ |  |  |  |  |  |  |  |  |  |  |  |  | |
| Chest radiograph | X ^5^ |  |  |  |  |  |  |  |  |  |  |  |  | |
| CD4 cell count, if HIV positive ^6^ |  |  | X |  |  |  |  |  |  |  |  |  |  | |

1. Visual acuity and color vision testing will be done according to standard site practice.
2. Must be sent to lab that will be used in the study (unless sent to that lab within 3 days or less).
3. For women of child-bearing potential.
4. An HIV test need not be performed if the patient has documented HIV infection or has written documentation of a negative test within 6 months or less.
5. Unless results within 14 days or less are available.
6. Patients found to have HIV infection, per the result of testing done at screening, will have blood drawn for a CD4 at the week 2 study visit (or at the first study visit after HIV infection is documented). Patients with documented HIV infection at screening, for whom written results of CD4 testing done within 6 months prior to the start of study therapy cannot be documented, will have blood drawn for this test as part of the study at the week 2 study visit.
7. 2 sputum specimens will be obtained at the end of intensive phase therapy. These two specimens must be obtained 0 to 7 days after the completion of the intensive phase therapy (preferably, one on the day of completion and one within 7 days of completion) and prior to the patient receiving more than one dose of continuation phase therapy.
8. Sputum specimens should be obtained until there have been 2 consecutive culture-negative specimens.
9. Study subjects receiving TB treatment for 9 months will have study visits at 7, 8, and 9 months.
10. Height should be measured only once, at the time of screening.

**Appendix E -** National Cancer Institute Common Toxicitiy Criteria (CTC)

# CTC Version 2.0 Publish Date: April 30, 1999

Cancer Therapy Evaluation Program 1 Revised March 23, 1998

Common Toxicity Criteria, Version 2.0

DCTD, NCI, NIH, DHHS March 1998

| ALLERGY/IMMUNOLOGY | | | | | |
| --- | --- | --- | --- | --- | --- |
| Grade | | | | | |
| Adverse Event | 0 | 1 | 2 | 3 | 4 |
| Allergic reaction/  hypersensitivity  (including drug fever) | none | transient rash,  drug fever  <38°C  (<100.4°F) | urticaria, drug  fever <38°  <100.4°F),  and/or  asymptomatic  bronchospasm | symptomatic  bronchospasm,  requiring  parenteral  medication(s),  with or without  urticaria;  allergy related  edema/  angioedema | anaphylaxis |
| Note: Isolated urticaria, in the absence of other manifestations of an allergic or hypersensitivity reaction, is graded  in the DERMATOLOGY/SKIN category. | | | | | |
| Allergic rhinitis  (including  sneezing, nasal  stuffiness,  postnasal drip) | none | mild, not  requiring  treatment | moderate,  requiring  treatment | - | - |
| Autoimmune reaction | none | serologic or other  evidence of  autoimmune  reaction  but patient is  asymptomatic  (e.g.,vitiligo), all  organ function is  normal  and no treatment  is required | evidence of  autoimmune  reaction  involving non-  essential organ  or function  (e.g.,  hypothyroidis),  requiring  treatment other  than immuno  suppressive  drugs | reversible  autoimmune  reaction  involving  function of  major  organ or other  adverse event  (e.g., transient  colitis anemia),  requiring short  term immuno  suppressive  treatment | autoimmune  reaction  causing  major grade 4  organ  dysfunction;  progressive and  irreversible  reaction long-term  administration of  high dose  immuno  suppressive  therapy  required |

| Also consider Hypothyroidism, Colitis, Hemoglobin, Hemolysis | | | | | |
| --- | --- | --- | --- | --- | --- |
| Serum sickness | None | - | - | Present | - |
| Urticaria is graded in the DERMATOLOGY/SKIN category if it occurs as an isolated symptom. If it occurs with  other manifestations of allergic Or hypersensitivity reaction, grade as Allergic reaction/hypersensitivity above. | | | | | |
| Vasculitis | none | mild, not  requiring  treatment | symptomatic,  requiring  medication | requiring steroids | ischemic changes  or requiring  amputation |
| Allergy/  Immunology  Other  (Specify,  __________) | none | mild | moderate | severe | life-threatening or  disabling |
| **AUDITORY/HEARING** | | | | | |
| Conductive hearing loss is graded as Middle ear/hearing in the AUDITORY/HEARING category. | | | | | |
| Earache is graded in the PAIN category. | | | | | |
| External auditory canal | normal | external otitis  with erythema or  dry desquamation | external otitis  with moist  desquamation | external otitis  with discharge,  mastoiditis | necrosis of the  canal soft tissue  or bone |
| Note: Changes associated with radiation to external ear (pinnae) are graded under Radiation dermatitis in the DERMATOLOGY/SKIN category. | | | | | |
| Inner ear/hearing | normal | hearing loss on  audiometry only | tinnitus or  hearing loss,  not requiring  hearing aid or  treatment | tinnitus or  hearing loss,  correctable with  hearing aid or  treatment | severe unilateral  or bilateral  hearing loss  (deafness), not  correctable |
| Middle ear/hearing | normal | serous otitis  without subjective  decrease in  hearing | serous otitis or  infection  requiring  medical  ntervention; subjective  decrease in  hearing;  rupture of  tympanic  membrane with  discharge | otitis with  discharge,  mastoiditis or  conductive  hearing loss | necrosis of the  canal soft tissue  or bone |

| Auditory/Hearing  Other  (Specify,__________) | normal | mild | moderate | severe | severe life  threatening or  disabling |
| --- | --- | --- | --- | --- | --- |
| **BLOOD/BONE MARROW** | | | | | |
| Bone marrow cellularity | normal for age | mildly  hypocellular or  25% reduction  from normal  cellularity for  age | moderately  hypocellular  or >25 - <50%  reduction from  normal  cellularity for age or >2  but <4 weeks to  recovery of  normal  bone marrow cellularity | severely  hypocellular or  >50 - <75%  reduction  in cellularity for  age or 4 - 6  weeks to  recovery  of normal bone marrow cellularity | aplasia or >6  weeks to  recovery of  normal  bone marrow  cellularity |
| CD4 count | WNL | <LLN - 500/mm _3_ | 200- <500/mm _3_ | 50 - <200/mm _3_ | <50/mm _3_ |
| Hemoglobin (Hgb) | WNL | <LLN - 10.0 g/dL  <LLN - 100 g/L  <LLN - 6.2 mmol/L | 8.0-<10.0 g/dl  80 - <100 g/L  4.9 - <6.2 mmol/L | 6.5 - <8.0 g/dL  65 - <80 g/L  4.0 - <4.9 mmol/L | <6.5 g/dL  <65 g/L  <4.0 mmol/L |
| Leukocytes (total WBC) | WNL | <LLN- 3.0x10 _9_ /L  <LLN- 3000/mm_3_ | >2-<3.0x10 _9_ /L  >2000 -  <3000/mm _3_ | >1.0 - <2.0 x  10_9_ /L  >1000 -  <2000/mm _3_ | <1.0 x 10 _9_ /L  <1000/mm _3_ |
| Neutrophils/  granulocytes  (ANC/AGC) | WNL | >1.5 - <2.0 x 10/L  >1500  <2000/mm _3_ | >1.0 - <1.5 x  10_9_ /L  >1000<  1500/mm _3_ | >0.5 - <1.0 x  10_9_ /L  >500  <1000/mm _3_ | <0.5 x 10 _9_ /L  <500/mm _3_ |
| Platelets | WNL | <LLN - 75.0 x  10 _9_ /L  <LLN  75,000/mm _3_ | >50.0 - <75.0 x  10 _9_ /L  >50,000 -  <75,000/mm _3_ | >10.0 - <50.0 x  10 _9_ /L  >10,000 -  <50,000/mm _3_ | <10.0 x 10 _9_ /L  <10,000/mm _3_ |
| Blood/Bone  Marrow - Other  (Specify,_____  ____) | none | mild | moderate | severe | life-threatening or  disabling |

| CARDIOVASCULAR (ARRHYTHMIA) | | | | | |
| --- | --- | --- | --- | --- | --- |
| Conduction  abnormality/  Atrioventricular heart block | none | asymptomatic, not  requiring  treatment  (e.g., Mobitz type  I second-degree  AV block,  Wenckebach) | symptomatic,  but not  requiring  treatment | symptomatic  And requiring  treatment  (e.g., Mobitz type II,second-degree AV  block, third  degree AV  block) | life-threatening  (e.g., arrhythmia  associated  with CHF,  hypotension,  syncope, shock) |
| Nodal/junctional  arrhythmia/  dysrhythmia | none | asymptomatic, not  requiring  treatment | symptomatic,  but not  requiring treatment | symptomatic  and requiring  treatment | life-threatening  (e.g.,arrhythmia  associated  with CHF, hypotension,  syncope, shock) |
| Palpitations  Note: Grade  palpitations only  in the absence of  a documented  arrhythmia. | none | present | - | - | - |
| Prolonged QTc  interval  (QTc >0.48 seconds) | none | asymptomatic, not  requiring  treatment | symptomatic,  but not  requiring  treatment | symptomatic  and requiring  treatment | life-threatening  (e.g.,arrhythmia  associated with  CHF,  hypotension,  syncope, shock) |
| Sinus bradycardia | none | asymptomatic, not  requiring  treatment | symptomatic,  but not  requiring  treatment | symptomatic  and requiring  treatment | life-threatening  (e.g.,arrhythmia\  associated  with CHF,  hypotension,  syncope, shock) |
| Sinus tachycardia | none | asymptomatic, not  requiring  treatment | symptomatic,  but not  requiring treatment | symptomatic  and requiring  treatment of  underlying  cause | - |

| Supraventricular  arrhythmias  (SVT/atrial  fibrillation/  flutter) | none | asymptomatic, not  requiring  treatment | symptomatic,  but not  requiring  treatment | symptomatic  and requiring  treatment | life-threatening  (e.g., arrhythmia  associated  with CHF, hypotension,  syncope, shock) |
| --- | --- | --- | --- | --- | --- |
| Syncope (fainting) is graded in the NEUROLOGY category. | | | | | |
| Vasovagal episode | none | - | present without loss of  consciousness | present with loss of  consciousness | - |
| Ventricular arrhythmia  (PVCs/bigeminy/trigeminy/  ventricular tachycardia) | none | asymptomatic, not  requiring  treatment | symptomatic,  but not  requiring  treatment | symptomatic  and requiring  treatment | life-threatening  (e.g.,arrhythmia  associated  with CHF,  hypotension,  syncope, shock) |
| Cardiovascular/  Arrhythmia  Other (Specify,  ___________) | none | asymptomatic, not  requiring  treatment | symptomatic,  but not  requiring  treatment | symptomatic,  and requiring  treatment of  underlying  cause | life-threatening  (e.g.,arrhythmia  associated with  CHF,hypotension,  syncope, shock) |
| CARDIOVASCULAR (GENERAL) | | | | | |
| Acute vascular leak  syndrome | absent | - | symptomatic,  but not  requiring fluid  support | respiratory  compromise  or requiring  fluids | life-threatening;  requiring pressor  support and/or  ventilatory support |
| Cardiac  ischemia/  infarction | none | non-specific T  wave flattening or  changes | asymptomatic,  ST - and T  wave changes  suggesting  ischemia | angina without  evidence of  infarction | acute myocardial  infarction |

| Cardiac left ventricular  function | normal | asymptomatic  decline of resting  ejection fraction  of .>10% but  <20% of baseline  value;shortening  fraction >24% but  <30% | asymptomatic\  but resting  ejection fraction  below LLN for  laboratory or  decline of  resting ejection  fraction >20%  of baseline  value;<24%  shortening  fraction | CHF responsive  to treatment | severe or  refractory  CHF or requiring  intubation |
| --- | --- | --- | --- | --- | --- |
| CNS cerebrovascular ischemia is graded in the NEUROLOGY category. | | | | | |
| Edema | none | asymptomatic, not  requiring therapy | symptomatic,  requiring  therapy | symptomatic  edema limiting  function and  unresponsive to  therapy or  requiring drug  discontinuation | anasarca (severe  generalized  edema) |
| Hypertension | none | asymptomatic,  transient increase  by >20 mmHg  (diastolic) or to  >150/100* if  previously WNL;  not requiring  treatment | recurrent or  persistent  or symptomatic  increase by >20  mmHg  (diastolic) or to  >150/100* if  previously  WNL; not  requiring  treatment | requiring  therapy or  more intensive  therapy than  previously | hypertensive  crisis |
| Hypotension | none | changes, but not  requiring therapy  (including transient  orthostatic hypotension) | requiring brief  fluid  replacement or  other therapy  but not  hospitalization  no physiologic  consequences | Requiring  therapy and  sustained  medical  attention, but  resolves  without  persisting  physiologic  consequences | shock (associated  with acidemia and  impairing vital  organ functiondue  to tissue  hypoperfusion) |
| Notes: Angina or MI is graded as Cardiac-ischemia/infarction in the CARDIOVASCULAR (GENERAL) category. | | | | | |

| **CONSTITUTIONAL SYMPTOMS** | | | | | |
| --- | --- | --- | --- | --- | --- |
| Fatigue  (lethargy,malaise  asthenia) | none | increased fatigue  over baseline, but  not altering  normal activities | moderate (e.g.,  decrease in  performance  status  by 1 ECOG  level or 20%  Karnofsky or  *Lansk*y) or  causing  difficulty  performing  some activities | severe (e.g.,  decrease in  performance  status by  >2 ECOG  levels or 40%  Karnofsky or  *Lansk*y) or  loss of ability to  perform some  activities | bedridden or  disabling |
| Fever (in the absence of  neutropenia, where  neutropenia is defined as AGC <1.0 x 10 9 /L) | none | 38.0 - 39.0°C (100.4 -102.2°  F) | 39.1 - 40.0°C (102.3-104.0°  F ) | >40.0°C (>104.0°F ) for  <24hrs | >40.0°C (>104.0°F ) for  >24hrs |
| Also consider Allergic reaction/hypersensitivity.  Note: The temperature measurements listed above are oral or tympanic. | | | | | |
| Hot flashes/flushes are graded in the ENDOCRINE category. | | | | | |
| Rigors, chills | none | mild, requiring  symptomatic treatment  (e.g., blanket) or non-narcotic medication | severe and/or  prolonged, requiring  narcotic  medication | not responsive to narcotic medication | - |
| Sweating  (diaphoresis) | normal | mild and occasional | frequent or drenching | - | - |
| Weight gain | <5% | 5 - <10% | 10 - <20% | >20% | - |
| Also consider Ascites, Edema, Pleural effusion (non-malignant). | | | | | |
| Weight loss | <5% | 5 - <10% | 10 - <20% | >20% | - |
| Also consider Vomiting, Dehydration, Diarrhea. | | | | | |
| Constitutional Symptoms Other  (Specify,___  ____) | none | mild | moderate | severe | life-threatening or  disabling |

| DERMATOLOGY/SKIN | | | | | |
| --- | --- | --- | --- | --- | --- |
| Alopecia | normal | mild hair loss | pronounced hair loss | - | - |
| Bruising  in absence of grade 3 or 4  thrombo-  cytopenia | none | localized or in  dependent area | generalized | - | - |
| Note: Bruising resulting from grade 3 or 4 thrombocytopenia is graded as Petechiae/purpura and Hemorrhage/bleeding with grade 3 or 4 thrombocytopenia in the  HEMORRHAGE category, not in the DERMATOLOGY/SKIN category. | | | | | |
| Dry skin | normal | controlled with  emollients | not controlled  with emollients | - |  |
| Erythema multiforme (e.g.,  Stevens-Johnson  syndrome,  toxic epidermal necrolysis) | absent | _ | scattered, but not generalized eruption | severe or requiring IV  fluids (e.g.  generalized  rash or painful  stomatitis) | life-threatening (e.g.,exfoliative or ulcerating dermatitis or requiring enteral or parenteral  nutritional  support) |
| Flushing | absent | present | - | - | - |
| Hand-foot skin reaction | none | skin changes or  dermatitis without pain (e.g., erythema, peeling) | skin changes with pain,  not interfering  with function | skin changes with pain,  interfering with  function | - |
| Injection site reaction | none | pain or itching or  erythema | pain or swelling, with  inflammation or  phlebitis | ulceration or necrosis  that is severe or  prolonged, or  requiring  surgery | - |
| Nail changes | normal | discoloration or ridging (koilonychia) or pitting | partial or complete loss  of nail(s) or pain in nailbeds | - | - |
| Petechiae is graded in the HEMORRHAGE category. | | | | | |
| Photosensitivity | none | painless erythema | painful erythema | erythema with  desquamation | - |
| none | generalized  pigmentation  changes | - | - |  | |
| Pruritus | none | mild or localized,  relieved  spontaneously  or by local  measures | intense or widespread,  relieved  spontaneously  or by systemic  measures | intense or widespread  and poorly  controlled  despite  treatment | - |
| Purpura is graded in the HEMORRHAGE category. | | | | | |
| Rash/  desquamation | none | macular or papular  eruption or erythema  without associated  symptoms | macular or papular  eruption or erythema  with pruritus or  other associated  symptoms  covering <50%  of body  surface or localized  desquamation  or other lesions  covering <50%  of body surface  area | symptomatic  generalized  erythroderma or  macular,papular  or vesicular  eruption or  desquamation  covering  >50% of body  surface area | generalized  exfoliative  dermatitis or  ulcerative dermatitis |
| Also consider Allergic reaction/hypersensitivity. multiforme in the DERMATOLOGY/SKIN category.  Note: Stevens-Johnson syndrome is graded separately as Erythema | | | | | |
| Urticaria  (hives, welts, wheals) | none | requiring no medication | requiring PO or topical  treatment or IV  medication or  steroids for <24  hours | requiring IV medication  or steroids for  >24 hours | - |
| Wound-infectious | none | cellulitis | superficial infection | infection requiring IV  antibiotics | necrotizing fasciitis |
| Wound-non-infectious | none | incisional separation | incisional hernia | fascial disruption  without  evisceration | fascial disruption with evisceration |
| Dermatology/  Skin - Other  (Specify, ________) | none | mild | moderate | severe | life-threatening or  disabling |
| ENDOCRINE | | | | | |
| Cushingoid appearance (e.g.,  moon face, buffalo hump,  centripetal obesity,  cutaneous striae)  Also consider Hyperglycemia, Hypokalemia. | absent | - | present | - | - |
| Feminization of  male | absent | - | - | present | - |
| Gynecomastia | none | mild | pronounced or painful | pronounced or painful and requiring  surgery | - |
| Hot flashes/flushes | none | mild or no more than 1 per day | moderate and greater than 1 per day | - | - |
| Hypothyroidism | absent | asymptomatic,  TSH elevated, no therapy given | symptomatic or thyroid replacement treatment given | patient hospitalized for  manifestations  of  hypothyroidism | myxedema coma |
| Masculinization of female | absent | - | - | present | - |
| SIADH (syndrome of  Inappropriate  antidiuretic  hormone) | absent | - | - | present | - |
| Endocrine - Other (Specify, __________) | none | mild | moderate | severe | life-threatening or  disabling |
| GASTROINTESTINAL | | | | | |
| Amylase is graded in the METABOLIC/LABORATORY category. | | | | | |
| Anorexia | none | loss of appetite | oral intake significantly  decreased | requiring IV fluids | requiring feeding tube or parenteral nutrition |
| Ascites (non-malignant) | none | asymptomatic | symptomatic, requiring diuretics | symptomatic, requiring  therapeutic  paracentesis | life-threatening  physiologic  consequences |
| Colitis | none | - | abdominal pain with mucus and/or blood in  stool | abdominal pain, fever,change in bowel habits  with ileus or peritoneal  signs, and radiographic  or biopsy  documentation | perforation or requiring surgery or toxic megacolon |
| Also consider Hemorrhage/bleeding with grade 3 or 4 thrombocytopenia, Hemorrhage/bleeding without grade 3 or 4 thrombocytopenia, Melena/GI bleeding, Rectal bleeding/hematochezia, Hypotension. | | | | | |
| Constipation | none | requiring stool softener or dietary modification | requiring  laxatives | obstipation requiring manual evacuation or  enema | obstruction or toxic megacolon |
| Dehydration | none | dry mucous membranes  and/or diminished  skin turgor | requiring IV fluid  replacement (brief) | requiring IV fluid replacement (sustained) | physiologic  consequences requiring  intensive care;  hemodynamic collapse |
| Also consider Diarrhea, Vomiting, Stomatitis/pharyngitis (oral/pharyngeal mucositis), Hypotension. | | | | | |
| Diarrhea  patients without  colostomy: | none | increase of <4  stools/day over  pre-treatment | increase of 4-6  stools/day, or  nocturnal stools | increase of >7  stools/day or  incontinence; or  need for  parenteral  support  for dehydration | physiologic  consequences  requiring  intensive care; or  hemodynamic  collapse |
| patients with a colostomy | none | mild increase in loose, watery colostomy output compared with  pretreatment | moderate increase in  loose, watery  colostomy  output  compared with  pretreatment,  but not  interfering with  normal activity | severe increase in loose,watery colostomy  output  compared with  pretreatment,  interfering  with normal  activity | physiologic  consequences,  requiring  intensive care; or  hemodynamic  collapse |

| Duodenal ulcer  (requires  radiographic or  endoscopic  documentation) | none | - | requiring medical  management or non-surgical treatment | uncontrolled by  outpatient  medical  management;  requiring  hospitalization | perforation or  bleeding,  requiring  emergency  surgery |
| --- | --- | --- | --- | --- | --- |
| Dyspepsia/  heartburn | none | mild | moderate | severe | - |
| Dysphagia,  esophagitis,  odynophagia  (painful  swallowing) | none | mild dysphagia, but can eat regular diet | dysphagia, requiring  predominantly  pureed, soft, or  liquid diet | dysphagia,  requiring IV  hydration | complete  obstruction  (cannot swallow  saliva) requiring  enteral or  parenteral  nutritional  support, or  perforation |
| Fistula  pharyngeal | none | - | - | present | requiring surgery |
| Fistula  rectal/anal | none | - | - | present | requiring surgery |
| Flatulence | none | mild | moderate | - | - |
| Gastric ulcer  (requires  radiographic or  endoscopic  documentation) | none | - | requiring medical management or non-surgical treatment | bleeding without  perforation,  uncontrolled by  outpatient  medical  management;  requiring  hospitalization  or surgery | perforation or bleeding, requiring  emergency  surgery |
| Also consider Hemorrhage/bleeding with grade 3 or 4 thrombocytopenia, Hemorrhage/bleeding without grade 3 or 4  thrombocytopenia. | | | | | |
| Gastritis | none | - | requiring medical  management or non-surgical treatment | uncontrolled by out-patient medical  management;  requiring  hospitalizatioor  surgery | life-threatening  bleeding,requiring  emergency  surgery |
| Also consider Hemorrhage/bleeding with grade 3 or 4 thrombocytopenia, Hemorrhage/bleeding without grade 3 or 4  thrombocytopenia.  Hematemesis is graded in the HEMORRHAGE category.  Hematochezia is graded in the HEMORRHAGE category as Rectal bleeding/hematochezia. | | | | | |

| Ileus (or  Neuro  constipation) | none | - | intermittent, not  requiring intervention | requiring non-surgical  intervention | requiring surgery |
| --- | --- | --- | --- | --- | --- |
| Mouth dryness | normal | mild | moderate | - | - |
| Mucositis  Notes: Mucositis not due to radiation is graded in the GASTROINTESTINAL category for specific sites: Colitis, Esophagitis, Gastritis, Stomatitis/pharyngitis  (oral/pharyngeal mucositis), and Typhlitis; or the RENAL/GENITOURINARY category for Vaginitis.  Radiation-related mucositis is graded as Mucositis due to radiation. | | | | | |
| Nausea | none | able to eat | oral intake significantly  decreased | no significant intake,requiring IV fluids | - |
| Pancreatitis | none | - | - | abdominal pain with pancreatic enzyme elevation | complicated by  shock (acute  circulatory  failure) |
| Also consider Hypotension.  Note: Amylase is graded in the METABOLIC/LABORATORY category.  Pharyngitis is graded in the GASTROINTESTINAL category as Stomatitis/pharyngitis (oral/pharyngeal mucositis) | | | | | |
| Vomiting | none | 1 episode in 24 hours over pretreatment | 2-5 episodes in 24 hours over pretreatment | >6 episodes in  24 hours  over  pretreatment; or  need for IV  fluids | requiring  parenteral  nutrition; or  physiologic  consequences  requiring  intensive care;  hemodynamic  collapse |
| Also consider Dehydration.  Weight gain is graded in the CONSTITUTIONAL SYMPTOMS category.  Weight loss is graded in the CONSTITUTIONAL SYMPTOMS category. | | | | | |

| HEMORRHAGE | | | | | |
| --- | --- | --- | --- | --- | --- |
| Notes: Transfusion in this section refers to pRBC infusion.  For any bleeding with grade 3 or 4 platelets (<50,000), always grade Hemorrhage/bleeding with grade 3 or 4  thrombocytopenia. Also consider Platelets,Transfusion: pRBCs, and Transfusion: platelets in addition to grading  severity by grading the site or type of bleeding.  If the site or type of Hemorrhage/bleeding is listed, also use the grading that incorporates the site of bleeding: CNS Hemorrhage/bleeding, Hematuria, Hematemesis, Hemoptysis, Hemorrhage/bleeding with surgery, Melena/lower GI bleeding, Petechiae/purpura (Hemorrhage/bleeding into skin), Rectal bleeding/hematochezia, Vaginal bleeding.  If the platelet count is>50,000 and the site or type of bleeding is listed, grade the specific site. If the site or type is not listed and the platelet count is >50,000, grade Hemorrhage/bleeding without grade 3 or 4 thrombocytopenia and specify the site or type in the OTHER category. | | | | | |
| Hemorrhage/  bleeding with  grade 3 or 4  thrombocyto-  penia | none | mild without  transfusion |  | requiring transfusion | catastrophic bleeding,  requiring major non-elective intervention |
| Also consider Platelets, Hemoglobin, Transfusion: platelets, Transfusion: pRBCs, site or type of bleeding. If the site  is not listed, grade as Hemorrhage-Other (Specify site, ___________).  Note: This adverse event must be graded for any bleeding with grade 3 or 4 thrombocytopenia. | | | | | |
| Hemorrhage/  Bleeding without  grade 3 or 4  thrombocyto-  penia | none | mild without  transfusion |  | requiring transfusion | catastrophic bleeding  requiring major  non-elective  intervention |
| Also consider Platelets, Hemoglobin, Transfusion: platelets, Transfusion: pRBCs, Hemorrhage - Other (Specify site, ___________).  Note: Bleeding in the absence of grade 3 or 4 thrombocytopenia is graded here only if the specific site or type of bleeding is not listed elsewhere in the  HEMORRHAGE category. Also grade as Other in the HEMORRHAGE category. | | | | | |
| CNS  hemorrhage/  bleeding | none | - | - | bleeding noted  on CT or other  scan with no  clinical  consequences | hemorrhagic  stroke or  hemorrhagic  vascular  event (CVA) with  neurologic signs  and symptoms |
| Epistaxis | none | mild without  transfusion | - | requiring transfusion | catastrophic bleeding,  requiring major  non-elective  intervention |
| Hematemesis | none | mild without  transfusion | - | requiring transfusion | catastrophic bleeding,  requiring major non-  elective intervention |
| Hematuria  (in the absence of  vaginal bleeding) | none | microscopic only | Intermittent  Gross bleeding,  no clots | persistent gross  bleeding or  clots; may  require  catheterization  or  instrumentatio  or transfusion | open surgery or  necrosis or deep  bladder ulceration |
| Hemoptysis | none | mild without  transfusion | - | requiring transfusion | catastrophic bleeding,  requiring major non-elective intervention |
| Hemorrhage/  bleeding  associated with  surgery | none | mild without  transfusion | - | requiring transfusion | catastrophic bleeding,  requiring major  non-elective  intervention |
| Note: Expected blood loss at the time of surgery is not graded as an adverse event. | | | | | |
| Melena/GI bleeding | none | mild without  transfusion | - | requiring transfusion | catastrophic bleeding,  requiring major  non-elective  intervention |
| Petechiae/  purpura  (hemorrhage/  bleeding into  skin or mucosa) | none | rare petechiae of  skin | petechiae or  purpura in  dependent areas  of skin | generalized  petechiae or  purpura of skin  or petechiae of  any mucosal  site | - |
| Rectal bleeding/  hematochezia | none | mild without  transfusion or  medication | persistent,  requiring  medication  (e.g., steroid  suppositories)  and/or  break from radiation  treatment | requiring transfusion | catastrophic  bleeding,  requiring major  non-elective  intervention |

| Vaginal bleeding | none | spotting, requiring  <2 pads per day | requiring >2  pads per  day, but not  requiring  transfusion | requiring transfusion | catastrophic  bleeding,  requiring major  non-elective  intervention |
| --- | --- | --- | --- | --- | --- |
| Hemorrhage  Other  (Specify site, ___________) | none | mild without  transfusion | - | requiring transfusion | catastrophic bleeding,  requiring major  non-elective  intervention |
| HEPATIC | | | | | |
| Alkaline  phosphatase | WNL | >ULN - 2.5 x  ULN | >2.5 - 5.0 x ULN | >5.0 - 20.0 x ULN | >20.0 x ULN |
| Bilirubin | WNL | >ULN - 1.5 x  ULN | >1.5 - 3.0 x ULN | >3.0 - 10.0 x ULN | >10.0 x ULN |
| GGT   -( Glutamyl  transpeptidase) | WNL | >ULN - 2.5 x  ULN | >2.5 - 5.0 x ULN | >5.0 - 20.0 x ULN | >20.0 x ULN |
| Hepatic  enlargement | absent | - | - | present | - |
| Note: Grade Hepatic enlargement only for treatment related adverse event including Veno-Occlusive Disease. | | | | | |
| Hypo-  albuminemia | WNL | <LLN - 3 g/dL | >2 - <3 g/dL | <2 g/dL | - |
| Liver  dysfunction/  failure  (clinical) | Normal | - | - | asterixis | encephalopathy or coma |
| Portal vein flow | Normal | - | decreased portal  vein flow | reversal/retro-  grade portal vein  flow | - |
| **SGOT (AST)** (serum glutamic  oxaloacetic  transaminase) | WNL | >ULN - 2.5 x ULN | >2.5 - 5.0 x ULN | >5.0 - 20.0 x ULN | >20.0 x ULN  SGPT (ALT) |
| (serum glutamic  pyruvic  transaminase) | WNL | >ULN - 2.5 x ULN | >2.5 - 5.0 x ULN | >5.0 - 20.0 x ULN | >20.0 x ULN |
| Hepatic -  Other (Specify,  __________) | None | mild | moderate | severe | life-threatening or  disabling |

| INFECTION/FEBRILE NEUTROPENIA | | | | | |
| --- | --- | --- | --- | --- | --- |
| Catheter-related  infection | None | mild, no active  treatment | moderate,  localized  infection,  requiring  local or oral  treatment | severe, systemic  infection, requiring IV  antibiotic or antifungal  treatment or  hospitalization | life-threatening sepsis  (e.g., septic shock). |
| Febrile  neutropenia  (fever of  unknown origin  without clinically  or  microbiologically  documented  infection)  (ANC <1.0 x 10 ^9^ /L, fever  >38.5°C) | None | - | - | Present | life-threatening  sepsis (e.g., septic  shock) |
| Also consider Neutrophils.  Note: Hypothermia instead of fever may be associated with neutropenia and is graded here. | | | | | |
| Infection  Documented  clinically or  microbiologically  with grade 3 or 4  neutropenia  (ANC <1.0 x  10 _9_/L) Also  consider  Neutrophils. | none | - | - | present | life-threatening  sepsis (e.g., septic  shock) |
| Notes: Hypothermia instead of fever may be associated with neutropenia and is graded here.  In the absence of documented infection grade 3 or 4 neutropenia with fever is graded as Febrile neutropenia. | | | | | |
| Infection with  Unknown ANC | none | - | - | present | life-threatening  sepsis (e.g., septic  shock) |
| Note: This adverse event criterion is used in the rare case when ANC is unknown. | | | | | |

| Infection without  Neutropenia | none | mild, no active  treatment | moderate,  localized  infection,  requiring  local or oral treatment | severe, systemic  infection,  requiring IV  antibiotic or  antifungal  treatment, or  hospitalization | life-threatening sepsis  (e.g., septic shock) |
| --- | --- | --- | --- | --- | --- |
| Also consider Neutrophils.  Wound-infectious is graded in the DERMATOLOGY/SKIN category. | | | | | |
| Infection/Febrile  Neutropenia - Other  (Specify,_____  ____) | none | mild | moderate | severe | life-threatening or  disabling |
| LYMPHATICS | | | | | |
| Lymphatics | normal | mild lymphedema | moderate  lymphedema  requiring  compression;  lymphocyst | severe  lymphedema  limiting  function;  lymphocyst  requiring  surgery | severe  lymphedema  limiting function  with ulceration |
| Lymphatics -  Other (Specify,  __________) | none | mild | moderate | severe | life-threatening or  disabling |
| METABOLIC/LABORATORY | | | | | |
| Acidosis  (metabolic or  respiratory) | normal | pH<normal, but  >7.3 | - | pH <7.3 | pH <7.3 with life-  threatening physiologic  consequences |
| Alkalosis  (metabolic or  respiratory) | normal | pH >normal, but <7.5 | - | pH >7.5 pH | >7.5 with life-  threatening  physiologic  consequences |
| Amylase | WNL | >ULN - 1.5 x ULN | >1.5 - 2.0 x ULN | >2.0 - 5.0 x ULN | >5.0 x ULN |
| Bicarbonate | WNL | <LLN - 16  mEq/dL | 11 - 15 mEq/dL | 8 - 10 mEq/dL | <8 mEq/dL |
| CPK  (creatine  phosphokinase) | WNL | >ULN - 2.5 x  ULN | >2.5 - 5 x ULN | >5 - 10 x ULN | >10 x ULN |
| Hypercalcemia | WNL | >ULN - 11.5  mg/dL  >ULN - 2.9  mmol/L | >11.5 - 12.5  mg/dL  >2.9 - 3.1  mmol/L | >12.5 - 13.5  mg/dL  >3.1 - 3.4  mmol/L | >13.5 mg/dL  >3.4 mmol/L |
| Hyper-  Cholesterolemia | WNL | >ULN - 300  mg/dL  >ULN - 7.75  mmol/L | >300 - 400  mg/dL  >7.75 - 10.34  mmol/L | >400 - 500  mg/dL  >10.34 - 12.92  mmol/L | >500 mg/dL  >12.92 mmol/L |
| Hyperglycemia | WNL | >ULN - 160  mg/dL  >ULN - 8.9  mmol/L | >160 - 250  mg/dL  >8.9 - 13.9  mmol/L | >250 - 500  mg/dL  >13.9 - 27.8  mmol/L | >500 mg/dL  >27.8 mmol/L or  acidosis |
| Hyperkalemia | WNL | >ULN - 5.5  mmol/L | >5.5 - 6.0 mmol/L | >6.0 - 7.0 mmol/L | >7.0 mmol/L |
| Hyper-  Magnesemia | WNL | >ULN - 3.0  mg/dL  >ULN - 1.23  mmol/L | - | >3.0 - 8.0mg/dL  >1.23 - 3.30  mmol/L | >8.0 mg/dL  >3.30 mmol/L |
| Hypernatremia | WNL | >ULN - 150  mmol/L | >150 - 155 mmol/L | >155 - 160 mmol/L | >160 mmol/L |
| Hyper-  Triglyceridemia | WNL | >ULN - 2.5 x  ULN | >2.5 - 5.0 x ULN | >5.0 - 10 x ULN | >10 x ULN |
| Hyperuricemia | WNL | >ULN - <10  mg/dL  <0.59 mmol/L  without  physiologic  consequences | - | >ULN - <10 mg/dL  <0.59 mmol/L  with  physiologic  consequences | >10 mg/dL  >0.59 mmol/L |
| Also consider Tumor lysis syndrome, Renal failure, Creatinine, Hyperkalemia. | | | | | |
| Hypocalcemia | WNL | <LLN - 8.0  mg/dL  <LLN - 2.0  mmol/L | 7.0 - <8.0  mg/dL  1.75 - <2.0  mmol/L | 6.0 - <7.0  mg/dL  1.5 - <1.75  mmol/L | <6.0 mg/dL  <1.5 mmol/L |
| Hypoglycemia | WNL | <LLN - 55 mg/dL  <LLN - 3.0  mmol/L | 40 - <55 mg/dL  2.2 - <3.0  mmol/L | 30 - <40 mg/dL  1.7 - <2.2 mmol/L | <30 mg/dL  <1.7 mmol/L |
| Hypokalemia | WNL | <LLN - 3.0  mmol/L | - | 2.5 - <3.0 mmol/L | <2.5 mmol/L |
| Hypo-  Magnesemia | WNL | <LLN - 1.2  mg/dL  <LLN - 0.5  mmol/L | 0.9 - <1.2  mg/dL  0.4 - <0.5  mmol/L | 0.7 - <0.9  mg/dL  0.3 - <0.4  mmol/L | <0.7 mg/dL  <0.3 mmol/L |
| Hyponatremia | WNL | <LLN - 130  mmol/L | - | 120 - <130 mmol/L | <120 mmol/L |
| Hypo-  Phosphatemia | WNL | <LLN -2.5 mg/dL  <LLN - 0.8  mmol/L | >2.0 - <2.5  mg/dL  >0.6 - <0.8  mmol/L | >1.0 - <2.0  mg/dL  >0.3 - <0.6  mmol/L | <1.0 mg/dL  <0.3 mmol/L |
| Hypothyroidism is graded in the ENDOCRINE category. | | | | | |
| Lipase | WNL | >ULN- 1.5 x  ULN | >1.5 - 2.0 x ULN | >2.0 - 5.0 x ULN | >5.0 x ULN |
| Metabolic/  Laboratory -  Other (Specify,  __________) | none | mild | moderate | severe | life-threatening or  disabling |
| MUSCULOSKELETAL | | | | | |
| Arthralgia is graded in the PAIN category. | | | | | |
| Arthritis | none | mild pain with  inflammation,  erythema  or joint swelling  but not interfering  with function | moderate pain  with  inflammation,  erythema, or  joint swelling  interfering  with function,  but not  interfering with  activities of  daily living | severe pain with  inflammation,  erythema, or  joint swelling  and interfering  with activities  of daily living | disabling |
| Muscle weakness  (not due to  neuropathy) | normal | asymptomatic  with weakness on  physical exam | symptomatic  and interfering  with function, but not  interfering with  activities of  daily living | symptomatic and  interfering with  activities of daily living | bedridden or  disabling |
| Myalgia [tenderness or pain in muscles] is graded in the PAIN category. | | | | | |
| Myositis  (inflammation/  damage of  muscle) | none | mild pain, not  interfering with  function | pain interfering  with function  but not  interfering with  activities of  daily living | pain interfering  with function  and interfering  with activities  of daily living | bedridden or disabling |
| Also consider CPK.  Note: Myositis implies muscle damage (i.e., elevated CPK). | | | | | |

| Osteonecrosis  (avascular  necrosis) | none | asymptomatic and  detected by  imaging only | symptomatic  and interfering  with function, but not  interfering with  activities of  daily living | symptomatic  and interfering  with activities  of daily living | symptomatic; or  disabling |
| --- | --- | --- | --- | --- | --- |
| Musculoskeletal  - Other  (Specify,  __________) | none | mild | moderate | severe | life-threatening or  disabling |
| **NEUROLOGY** | | | | | |
| Aphasia receptive and/or expressive, is graded under Speech impairment in the NEUROLOGY category. | | | | | |
| Arachnoiditis/  meningismus/  radiculitis | absent | mild pain not  interfering  with function | moderate pain  interfering with  function, but  not interfering  with activities of daily living | severe pain  interfering  with activities  of daily living | unable to function  or perform  activities of  daily living;  bedridden;  paraplegia |
| Also consider Headache, Vomiting, Fever. | | | | | |
| Ataxia  (incoordination) | normal | asymptomatic  but abnormal  on physical  exam, and not  interfering with  function | mild symptoms  interfering with  function, but not  interfering with  activities of  daily living | moderate  symptoms  interfering with  activities of  daily living | bedridden or  disabling |
| CNS  Cerebrovascular  Ischemia | none | - | - | transient  ischemic event  or attack (TIA) | permanent event  (e.g., cerebral  vascular  accident) |
| CNS hemorrhage/bleeding is graded in the HEMORRHAGE category. | | | | | |
| Confusion | normal | confusion or  disorientation or  attention deficit  of brief duration;  resolves  spontaneously  with no sequelae | confusion or  disorientation or  attention deficit  of brief  duration; resolves  spontaneously  with no  sequelae | confusion or  disorientation or  attention deficit  interfering with  function, but not  interfering with  activities of  daily living | confusion or  delirium  interfering with  activities of  daily living  harmful to  others or  self; requiring  hospitalization |
| Cranial neuropathy is graded in the NEUROLOGY category as Neuropathy-cranial. | | | | | |
| Delusions | normal | - | - | present | toxic psychosis |
| Depressed level of  Consciousness | normal | somnolence or  sedation  not interfering  with function | somnolence or  sedation  interfering with  function, but not  interfering with  activities of  daily living | obtundation or  stupor; difficult  to arouse;  interfering with  activities of  daily living | coma |
| Note: Syncope (fainting) is graded in the NEUROLOGY category. | | | | | |
| Dizziness/light-headedness | none | not interfering with function | interfering with  function, but not  interfering with  activities of  daily living | interfering with  activities of  daily living | bedridden or  disabling |
| Dysphasia, receptive and/or expressive, is graded under Speech impairment in the NEUROLOGY category. | | | | | |
| Extrapyramidal/  Involuntary  movement/  restlessness | none | mild involuntary  movements not  interfering with  function | moderate  involuntary  movements  interfering  with function,  but not  interfering with  activities of  daily living | severe  involuntary  movements or  torticollis  interfering with  activities of  daily living | bedridden or  disabling |
| Hallucinations | normal | - | - | present | toxic psychosis |
| Headache is graded in the PAIN category. | | | | | |
| Insomnia | normal | occasional  difficulty  sleeping not  interfering  with function | difficulty  sleeping  interfering with  function, but not  interfering with  activities of daily living | frequent  difficulty  sleeping,  interfering  with activities of  daily  living | - |
| Note: This adverse event is graded when insomnia is related to treatment. If pain or other symptoms interfere with sleep do NOT grade as insomnia. | | | | | |
| Memory loss | normal | memory loss not  interfering with  function | memory loss  interfering  with function,  but not  interfering with  activities of daily living | memory loss  interfering  with activities of  daily  living | amnesia |
| Mood alteration  anxiety,  agitation | normal | mild mood  alteration  not interfering  with function | moderate mood  alteration  interfering  with function,  but not  interfering with  activities of  daily living | severe mood  alteration  interfering with  activities of  daily living | suicidal ideation  or danger to self |
| Mood alteration  Depression | normal | mild mood  alteration  not interfering  with function | moderate mood  alteration  interfering  with function,  but not  interfering with  activities of  daily living | severe mood  alteration  interfering with  activities of  daily living | suicidal ideation  or danger to self |
| Mood alteration-euphoria | normal | mild mood  alteration  not interfering  with function | moderate mood  alteration  interfering  with function,  but not  interfering with  activities of  daily living | severe mood  alteration  interfering with  activities of  daily living | danger to self |
| Neuropathic pain is graded in the PAIN category. | | | | | |
| Neuropathy-cranial | absent | - | present, not  interfering  with activities of  daily living | present,  interfering with  activities of  daily living | life-threatening,  disabling |
| Neuropathy-motor | normal | subjective  weakness but  no objective  findings | mild objective  weakness  interfering  with function,  but not  interfering with  activities of  daily living | Objective  weakness  interfering with  activities of  daily living | paralysis |
| Neuropathy-sensory | normal | loss of deep  tendon reflexes  or paresthesia  (including  tingling) but  not interfering  with function | objective  sensory loss  or paresthesia  (including  tingling),  interfering with  function, but not  interfering with  activities of  daily living | sensory loss or  paresthesia  interfering  with activities of  daily living | permanent  sensory loss  that interferes  with function |
| Nystagmus  Also Consider  Vision-double  vision. | absent | present | - | - | - |
| Personality/  Behavioral | normal | change, but not  disruptive to  patient or  family | disruptive to  patient or  family | disruptive to  patient and  family; requiring  mental health  intervention | harmful to  others or  self; requiring  hospitalization |
| Pyramidal tract  Dysfunction  (e.g.,  tone, hyperreflexia,  positive  Babinski, ( fine  motor  coordination) | normal | asymptomatic with  abnormality on physical  examination | symptomatic or  interfering with  function but not  interfering with  activities of daily living | interfering with  activities of daily living | bedridden or disabling;  paralysis |
| Seizure(s) | none | - | seizure(s) self  limited and  consciousness is  preserved | seizure(s) in  which  consciousness is  altered | seizures of any  type which are  prolonged,  repetitive, or  difficult to  control (e.g.,  status  epilepticus,  intractable  epilepsy) |
| Speech  Impairment  (e.g., dysphasia  or aphasia) | normal | - | awareness of  receptive  or expressive  dysphasia,  not impairing  ability to  communicate | receptive or  expressive  dysphasia,  impairing  ability to  communicate | inability to  communicate |
| Syncope  (fainting) | absent | - | - | present | - |
| Also consider CARDIOVASCULAR (ARRHYTHMIA), Vasovagal episode, CNS cerebrovascular ischemia | | | | | |
| Tremor | none | mild and brief or  intermittent but  not interfering  with function | moderate tremor  interfering with  function, but not  interfering with  activities of  daily living | severe tremor  interfering with  activities of  daily living | - |
| Vertigo | none | not interfering  with function | interfering with  function, but not  interfering with  activities of  daily living | interfering with  activities of  daily living | bedridden or  disabling |
| Neurology -  Other  (Specify,  __________) | none | mild | moderate | severe | life-threatening  or disabling |
| OCULAR/VISUAL | | | | | |
| Cataract | none | asymptomatic | symptomatic,  partial visual  loss | symptomatic,  visual loss  requiring  treatment  or interfering  with function | - |
| Conjunctivitis | none | abnormal  ophthalmologic  changes, but  asymptomatic or  symptomatic  without visual  impairment (i.e.,  pain and  irritation) | symptomatic and  interfering with  function, but not  interfering with  activities of  daily living | symptomatic and  interfering with  activities of  daily living | - |
| Dry eye | normal | mild, not  requiring  treatment | moderate or  requiring  artificial tears | - | - |
| Glaucoma | none | increase in  intraocular  pressure but no  visualloss | increase in  intraocular  pressure with  retinal changes | visual  impairment | unilateral or  bilateral  loss of  vision  (blindness) |
| Keratitis  (corneal  inflammation/  corneal  ulceration) | none | abnormal  ophthalmologic  changes but  asymptomatic or  symptomatic  without visual  impairment (i.e.,  pain and  irritation) | symptomatic and  interfering with  function, but not  interfering with  activities of  daily living | symptomatic and  interfering with  activities of daily living | unilateral or  bilateral  loss of vision  (blindness) |
| Tearing (watery  eyes) | none | mild: not  interfering  with function | moderate:  interfering  with function,  but not  interfering with  activities of  daily living | interfering with  activities of  daily living | - |
| Vision-blurred  Vision | normal | - | symptomatic and  interfering with  function, but not  interfering with  activities of  daily living | symptomatic and  interfering with  activities of  daily living | - |
| Vision-  double vision  (diplopia) | normal | - | symptomatic and  interfering with  function, but not  interfering with  activities of  daily living | symptomatic and  interfering with  activities of  daily living | - |
| Vision-flashing  lights/floaters | normal | mild, not  interfering  with function | symptomatic and  interfering with  function, but not  interfering with  activities of  daily living | symptomatic and  interfering with  activities of  daily living | - |
| Vision-night blindness  (nyctalopia) | normal | Abnormal  Electro  retinography  but  asymptomatic | symptomatic and  interfering with  function, but not  interfering with  activities of  daily living | symptomatic and  interfering with  activities of  daily living | - |
| Vision  Photophobia | normal | - | symptomatic and  interfering with  function, but not  interfering with  activities of  daily living | symptomatic and  interfering with  activities of  daily living | - |
| Ocular/Visual -  Other (Specify,  __________) | normal | mild | moderate | severe | unilateral or  bilateral loss of  vision  (blindness) |
| PAIN | | | | | |
| Abdominal pain or cramping | none | mild pain not  interfering  with function | moderate pain:  pain or  analgesics  interfering  with function,  but not  interfering with  activities of  daily living | severe pain: pain  or analgesics  severely  interfering with  activities of  daily living | disabling |
| Arthralgia  (joint pain) | none | mild pain not  interfering  with function | moderate pain:  pain or  analgesics  interfering  with function,  but not  interfering with  activities of  daily living | severe pain: pain  or analgesics  severely  interfering with  activities of  daily living | disabling |
| Arthritis (joint pain with clinical signs of inflammation) is graded in the MUSCULOSKELETAL category. | | | | | |
| Bone pain | none | mild pain not  interfering  with function | moderate pain:  pain or  analgesics  interfering  with function,  but not  interfering with  activities of  daily living | severe pain: pain  or analgesics  severely  interfering with  activities of  daily living | disabling |
| Chest pain  (non-cardiac and  non-pleuritic) | none | mild pain not  interfering  with function | moderate pain:  pain or  analgesics  interfering  with function,  but not  interfering with  activities of  daily living | severe pain: pain  or analgesics  severely  interfering with  activities of  daily living | disabling |
| Dysmenorrhea | none | mild pain not  interfering  with function | Moderate  pain:pain or  analgesics  interfering  with function,  but not  interfering with  activities of  daily living | severe pain: pain  or analgesics  severely  interfering with  activities of  daily living | disabling |
| Dyspareunia | none | mild pain not  interfering  with function | moderate pain  interfering with  sexual activity | severe pain  sexual activity | - |
| Dysuria is graded in the RENAL/GENITOURINARY category. | | | | | |
| Earache (otalgia) | none | mild pain not  interfering  with function | moderate pain:  pain or  analgesics  interfering  with function,  but not  interfering with  activities of  daily living | severe pain: pain  or analgesics  severely  interfering with  activities of  daily living | disabling |
| Headache | none | mild pain not  interfering  with function | moderate pain: pain or  analgesics  interfering  with function,  but not  interfering with  activities of  daily living | severe pain: pain  or analgesics  severely  interfering with  activities of  daily living | disabling. |
| Hepatic pain | none | mild pain not  interfering  with function | moderate pain:  pain or  analgesics  interfering  with function,  but not  interfering with  activities of  daily living | severe pain: pain  or analgesics  severely  interfering with  activities of  daily living | disabling |
| Myalgia  (muscle pain) | none | mild pain not  interfering  with function | moderate pain:  pain or  analgesics  interfering  with function,  but not  interfering with  activities of  daily living | severe pain: pain  or analgesics  severely  interfering with  activities of  daily living | disabling |
| Neuropathic  Pain  (e.g., jaw pain,  neurologic  pain, phantom  limb pain,  post-infectious  neuralgia, or  painful  neuropathies) | none | mild pain not  interfering  with function | moderate pain:  pain or  analgesics  interfering  with function,  but not  interfering with  activities of  daily living | severe pain: pain  or analgesics  severely  interfering with  activities of  daily living |  |
| Pelvic pain | none | mild pain not  interfering  with function | moderate pain:  pain or  analgesics  interfering  with function,  but not  interfering with  activities of  daily living | severe pain: pain  or  analgesics  severely  interfering with  activities of  daily living | disabling |
| Pleuritic pain | none | mild pain not  interfering  with function | moderate pain:  pain or  analgesics  interfering  with function,  but not  interfering with  activities of  daily living | severe pain: pain  or analgesics  severely  interfering with  activities of  daily living | disabling |
| Rectal or  perirectal pain  (proctalgia) | none | mild pain not  interfering  with function | moderate pain:  pain or  analgesics  interfering  with function,  but not  interfering with  activities of  daily living | severe pain: pain  or analgesics  severely  interfering with  activities of  daily living | disabling |
| Tumor pain  (onset or  exacerbation of  tumor pain due  to treatment) | none | mild pain not  interfering  with function | moderate pain:  pain or  analgesics  interfering  with function,  but not  interfering with  activities of  daily living | severe pain: pain  or analgesics  severely  interfering with  activities of  daily living | disabling |
| Tumor flare is graded in the SYNDROME category. | | | | | |
| Pain - Other  (Specify,  __________) | none | mild | moderate | severe | disabling |
| PULMONARY | | | | | |
| Adult  Respiratory  Distress  Syndrome  (ARDS) | absent | - | - | - | present |
| Apnea | none | - | - | - | present requiring  intubation. |
| Carbon  Monoxide  Diffusion  capacity (DLCO) | >90% of  pretreatment or  normal value | >75 - <90% of  pretreatment or  normal value | <50 - <75% of  pretreatment or  normal value | <25 - <50% of  pretreatment or  normal value | <25% of  pretreatment  or normal value |
| Cough | absent | mild, relieved by  non-  prescription  medication | Requiring  narcotic  antitussive | severe cough or  coughing  spasms,poorly  controlled or  unresponsive to  treatment | - |
| Dyspnea  (shortness of  breath) | normal | - | dyspnea on  exertion | dyspnea at  normal level  of activity | dyspnea at rest  or requiring  ventilator  support |
| FEV1 | >90% of  pretreatment or  normal value | >75 - <90% of  pretreatment or  normal value | >50 - <75% of  pretreatment or  normal value | >25 - <50% of  pretreatment or  normal value | <25% of  pretreatment  or normal value |
| Hiccoughs  (hiccups,  singultus) | none | mild, not  requiring  treatment | moderate,  requiring  treatment | severe,  prolonged, and  refractory to  treatment | - |
| Hypoxia | normal | - | decreased O2  saturation  with exercise | decreased O2  saturation  at rest, requiring  supplemental  oxygen | decreased O2  saturation,  requiring  pressure  support (CPAP)  or assisted  ventilation |
| Pleural effusion  (non-malignant) | none | asymptomatic  and not  requiring  treatment | symptomatic,  requiring  diuretics | symptomatic,  requiring  O2 or therapeutic  thoracentesis | life-threatening  (e.g.,requiring  intubation) |
| Pleuritic pain is graded in the PAIN category. | | | | | |
| Pneumonitis/  Pulmonary  Infiltrates | none | radiographic  changes  but  asymptomatic or  symptoms not  requiring  steroids | radiographic  changes  and requiring  steroids or  diuretics | radiographic  changes  and requiring  oxygen | radiographic  changes  and requiring  assisted  ventilation |
| Pneumothorax | none | no  Intervention  required | chest tube  required | sclerosis or  surgery required | life-threatening |
| Pulmonary embolism is graded as Thrombosis/embolism in the CARDIOVASCULAR (GENERAL) category. | | | | | |
| Pulmonary  Fibrosis | none | radiographic  changes,  but  asymptomatic or  symptoms not  requiring  steroids | requiring  steroids or  diuretics | requiring oxygen | requiring  assisted  ventilation |
| Note: Radiation-related pulmonary fibrosis is graded in the RTOG/EORTC Late Radiation Morbidity Scoring Scheme-Lung. (See Appendix IV) | | | | | |
| Voice  changes/  stridor/larynx  (e.g., hoarseness,  loss of voice,  laryngitis) | normal | mild or  intermittent  hoarseness | persistent  hoarseness,  but able to  vocalize;  may have mild  to moderate  edema | whispered  speech, not  able to vocalize;  may have  marked edema | marked  dyspnea/stridor  requiring  tracheostomy  or intubation |
| Notes: Cough from radiation is graded as cough in the PULMONARY category.  Radiation-related hemoptysis from larynx/pharynx is graded as Grade 4 Mucositis due to radiation in the GASTROINTESTINAL category. Radiation-related hemoptysis from the thoracic cavity is graded as Grade 4 Hemoptysis in the HEMORRHAGE category. | | | | | |
| Pulmonary -  Other  (Specify,  __________) | none | mild | moderate | severe | life-threatening  or disabling |
| RENAL/GENITOURINARY | | | | | |
| Bladder spasms | absent | mild symptoms,  not requiring  intervention | symptoms  requiring  antispasmodic | severe  symptoms  requiring  narcotic | - |
| Creatinine | WNL | >ULN - 1.5 x  ULN | >1.5 - 3.0 x  ULN | >3.0 - 6.0 x  ULN | >6.0 x ULN |
| Note: Adjust to age-appropriate levels for pediatric patients. | | | | | |
| Dysuria  (painful  urination) | none | mild symptoms  requiring no  intervention | symptoms  relieved with  therapy | symptoms not  relieved despite  therapy | - |
| Fistula or GU  Fistula  (e.g., vaginal,  vesicovaginal) | none | - | - | Requiring  intervention | Requiring  surgery |
| Hemoglobinuria | - | present | - | - | - |
| Hematuria (in the absence of vaginal bleeding) is graded in the HEMORRHAGE category. | | | | | |
| Incontinence | none | with coughing,  sneezing, etc. | spontaneous,  some control | no control (in  the absence of  fistula) | - |
| Operative injury  to bladder and/or  ureter | none | - | injury of bladder  with primary  repair | sepsis, fistula, or  obstruction  requiring  secondary  surgery; loss  of one kidney;  injury requiring  anastomosis  or  reimplantation | septic  obstruction of  both kidneys or  vesicovaginal  fistula  requiring  diversion |
| Proteinuria | normal or <0.15  g/24 hours | 1+ or 0.15 - 1.0  g/24 hours | 2+ to 3+ or 1.0 -  3.5 g/24 hours | 4+ or >3.5 g/24  hours | nephrotic  syndrome |
| Renal failure | none | - | - | requiring  dialysis, but  reversible | requiring  dialysis and  irreversible |
| Ureteral  Obstruction | none | unilateral, not  requiring  surgery | - | bilateral, not requiring  surgery | stent,  nephrostomy  tube, or surgery |
| Urinary  Lectrolyte  Wasting  (e.g., Fanconi’s  syndrome,  renal tubular  acidosis) | none | asymptomatic,  not requiring  treatment | mild, reversible  and manageable  with oral  replacement | reversible but  requiring  IV replacement | irreversible,  requiring  continued  replacement |
| Also consider Acidosis, Bicarbonate, Hypocalcemia, Hypophosphatemia. | | | | | |
| Urinary  Requency  /urgency | normal | increase in  frequency or  nocturia up to 2x  normal | increase >2 x  normal but  <hourly | hourly or more  with urgency, or  requiring  catheter | - |
| Urinary  Retention | normal | hesitancy or  dribbling,  but no  significant  residual urine;  retention  occurring during  the immediate  postoperative  period | hesitancy  requiring  medication or  occasional in/out  catheterization  (<4 x per week),  or operative  bladder atony  requiring  indwelling  catheter  beyond  immediate  postoperative  period but  for <6 weeks | requiring  frequent  in/out  catheterization  4 x per week)or  urological  intervention  (e.g., TURP,  suprapubic  tube,  urethrotomy) | bladder rupture |
| Urine color  Change  (not related to  other dietary  or physiologic  cause e.g.,  bilirubin,  concentrated  urine,  hematuria) | normal | asymptomatic,  change  in urine color | - | - | - |
| Vaginitis  (not due to  infection) | none | mild, not  requiring  treatment | moderate,  relieved with  treatment | severe, not  relieved  with treatment,  or ulceration not  surgery  requiring | ulceration  requiring  surgery |
| Renal/  Genitourinary  Other  (Specify, | none | mild | moderate | severe | life-threatening  or disabling |
| SECONDARY MALIGNANCY | | | | | |
| Secondary  initial primary  Malignancy -  Other  (Specify type,  __________)  excludes  metastasis from  initial primary | none | - | - | - | present |
| SEXUAL/REPRODUCTIVE FUNCTION Dyspareunia is graded in the PAIN category.  Dysmenorrhea is graded in the PAIN category. | | | | | |
| Erectile  Impotence | normal | mild (erections  impaired but  satisfactory) | moderate  (erections  impaired,  unsatisfactory  for intercourse) | no erections | - |
| Female sterility | normal | - | - | sterile | - |
| Irregular menses  (change from baseline) | normal | occasionally  irregular or  lengthened  interval, but  continuing  menstrual  cycles | very irregular,  but continuing  menstrual  cycles | persistent  amenorrhea | - |
| Libido | normal | decrease in  interest | severe loss of  interest | - | - |
| Male infertility | - | - | oligospermia  (low sperm  count) | azoospermia  (no sperm) | - |
| Vaginal dryness | normal | mild | requiring  treatment  and/or  interfering with  sexual function,  dyspareunia | - | - |
| Sexual/  Reproductive  Function - Other  (Specify,_____  ____) | none | mild | moderate | severe | disabling |
